# Mutation Clusters from Cancer Exome

**DOI:** 10.3390/genes8080201

**Published:** 2017-08-15

**Authors:** Zura Kakushadze, Willie Yu

**Affiliations:** 1 Quantigic^®^ Solutions LLC, 1127 High Ridge Road #135, Stamford, CT 06905, USA; 2 Business School & School of Physics 240, Free University of Tbilisi, David Agmashenebeli Alley, 0159 Tbilisi, Georgia; 3 Centre for Computational Biology, Duke-NUS Medical School, 8 College Road, Singapore 169857; willie.yu@duke-nus.edu.sg

**Keywords:** clustering, K-means, nonnegative matrix factorization, somatic mutation, cancer signatures, genome, exome, DNA, eRank, correlation, covariance, machine learning, sample, matrix, source code, quantitative finance, statistical risk model, industry classification

## Abstract

We apply our statistically deterministic machine learning/clustering algorithm *K-means (recently developed in https://ssrn.com/abstract=2908286) to 10,656 published exome samples for 32 cancer types. A majority of cancer types exhibit a mutation clustering structure. Our results are in-sample stable. They are also out-of-sample stable when applied to 1389 published genome samples across 14 cancer types. In contrast, we find in- and out-of-sample instabilities in cancer signatures extracted from exome samples via nonnegative matrix factorization (NMF), a computationally-costly and non-deterministic method. Extracting stable mutation structures from exome data could have important implications for speed and cost, which are critical for early-stage cancer diagnostics, such as novel blood-test methods currently in development.

## 1. Introduction and Summary

Unless humanity finds a cure, about a billion people alive today will die of cancer. Unlike other diseases, cancer occurs at the DNA level via somatic alterations in the genome. A common type of such mutations found in cancer is due to alterations to single bases in the genome (single nucleotide variations (SNVs)). These alterations are accumulated throughout the lifespan of an individual via various mutational processes, such as imperfect DNA replication during cell division or spontaneous cytosine deamination [1,2], or due to exposures to chemical insults or ultraviolet radiation [3,4], etc. The footprint left by these mutations in the cancer genome is characterized by distinctive alteration patterns known as cancer signatures.

Identifying all cancer signatures would greatly facilitate progress in understanding the origins of cancer and its development. Therapeutically, if there are common underlying structures across different cancer types, then treatment for one cancer type might be applicable to other cancer types, which would be great news. From a diagnostic viewpoint, the identification of all underlying cancer signatures would aid cancer detection and identification methodologies, including vital early detection [5]—according to American Cancer Society, late stage metastatic cancers of unknown origin represent about 2% of all cancers [6] and can make treatment almost impossible. Another practical application is prevention by pairing the signatures extracted from cancer samples with those caused by known carcinogens (e.g., tobacco, aflatoxin, UV radiation, etc.). At the end of the day, it all boils down to the question of usefulness: is there a small enough number of cancer signatures underlying all (100+) known cancer types, or is this number too large to be meaningful/useful? Thus, if we focus on 96 mutation categories of SNVs [7], we cannot have more than 96 signatures [8]. Even if the number of true underlying signatures is, say, of order 50, it is unclear whether they would be useful, especially within practical applications. On the other hand, if there are only about a dozen underlying cancer signatures, then the hope for an order of magnitude simplification may well be warranted.

The commonly-used method for extracting cancer signatures [9] is based on nonnegative matrix factorization (NMF) [10,11]. Thus, one analyzes SNV patterns in a cohort of DNA sequenced whole cancer genomes and organizes the data into a matrix $G_{i\mu }$, where the rows correspond to the N=96 mutation categories, the columns correspond to *d* samples and each element is a nonnegative occurrence count of a given mutation category in a given sample. Under NMF, the matrix *G* is then approximated via G≈WH, where $W_{iA}$ is an N×K matrix, $H_{A\mu }$ is a K×d matrix and both *W* and *H* are nonnegative. The appeal of NMF is its biologic interpretation, whereby the *K* columns of the matrix *W* are interpreted as the weights with which the *K* cancer signatures contribute to the N=96 mutation categories, and the columns of the matrix *H* are interpreted as the exposures to these *K* signatures in each sample. The price to pay for this is that NMF, which is an iterative procedure, is computationally costly, and depending on the number of samples *d*, it can take days or even weeks to run it. Furthermore, NMF does not fix the number of signatures *K*, which must be either guessed or obtained via trial and error, thereby further adding to the computational cost. Perhaps most importantly, NMF is a nondeterministic algorithm and produces a different matrix *W* in each run. (Each *W* corresponds to one in myriad local minima of the NMF objective function.) This is dealt with by averaging over many such *W* matrices obtained via multiple NMF runs (or samplings). However, each run generally produces a weights matrix $W_{iA}$ with columns (i.e., signatures) not aligned with those in other runs. Aligning or matching the signatures across different runs (before averaging over them) is typically achieved via nondeterministic clustering such as k-means. Therefore, the result, even after averaging, generally is both noisy [12] and nondeterministic, i.e., if this computationally-costly procedure (which includes averaging) is run again and again on the same data, generally it will yield different looking cancer signatures every time. Simply put, the NMF-based method for extracting cancer signatures is not designed to be even in-sample stable. Under these circumstances, out-of-sample stability cannot even be feasible (i.e., cancer signatures obtained from non-overlapping sets of samples can be dramatically different, and out-of-sample stability is crucial for practical usefulness, e.g., diagnostically).

Without in- and out-of-sample stability, practical therapeutic and diagnostic applications of cancer signatures would be challenging. For instance, suppose one sequences genome (or exome; see below) data from a patient sample (be it via a liquid biopsy, a blood test or some other (potentially novel) method). Let us focus on SNVs. We have a vector of occurrence counts for 96 mutation categories. We need a quick computational test to determine with a high enough confidence level whether (i) there is a cancer signature present in this data and (ii) which cancer type this cancer signature corresponds to (i.e., in which organ the cancer originated). If cancer signatures are not even in-sample stable, then we cannot trust them. They could simply be noise. Indeed, there is always somatic mutational noise present in such data, and this must be factored out of the data before extracting cancer signatures. A simple way to understand somatic mutational noise is to note that mutations (i) are already present in humans unaffected by cancer and (ii) such mutations, which are unrelated to cancer, are further exacerbated when cancer occurs, as it disrupts the normal operation of various processes (including repair) in the DNA. At the level of the data matrix *G*, in [13], we discussed a key component of the somatic mutational noise and gave a prescription for removing it [14]. However, there likely exist other, deeper sources of somatic mutational noise, which must be further identified and carefully factored out. Simply put, somatic mutational noise unequivocally is a substantial source of systematic error in cancer signatures.

However, then there is also the statistical error, which is large and due to the nondeterministic nature of NMF discussed above. This statistical error is exacerbated by the somatic mutational noise, but would be present even if this noise were somehow completely factored out. Therefore, the in-sample instability must somehow be addressed. We emphasize that, a priori, this does not automatically address out-of-sample stability, without which any therapeutic or diagnostic applications would still be farfetched. However, without in-sample stability, nothing is clear.

The problem at hand is nontrivial and requires a step-by-step approach, including identification of various sources of in-sample instability. One simple observation of [13] is that, if we work directly with occurrence counts $G_{i\mu }$ for individual samples, (i) the data are very noisy and (ii) the number of signatures is bound to be too large to be meaningful/useful if the number of samples is large. A simple way to deal with this is to aggregate samples by cancer types. In doing so, we have a matrix $G_{is}$, where *s* now labels cancer types, which is (i) less noisy and (ii) much smaller (96×n, where *n* is the number of cancer types), so the number of resultant signatures is much more reasonable [15]. Thus, such aggregation is helpful.

Still, even with aggregation, we must address nondeterminism (of NMF). To circumvent this, in [16], we proposed an alternative approach that bypasses NMF altogether. As we argue in [16], NMF is, at least to a certain degree, clustering in disguise, e.g., many COSMIC cancer signatures [17] obtained via NMF (augmented with additional heuristics based on biologic intuition and empirical observations) exhibit clustering substructure, i.e., in many of these signatures, there are mutation categories with high weights (“peaks” or “tall mountain landscapes”) with other mutation categories having small weights likely well within statistical and systematic errors. For all practical purposes, such low weights could be set to zero. Then, many cancer signatures would start looking like clusters, albeit some clusters could be overlapping between different signatures. Considering that various signatures may be somatic mutational noise artifacts in the first instance and statistical error bars are large, it is natural to wonder whether there are some robust underlying clustering structures present in the data, with the understanding that such structures may not be present for all cancer types. However, even if they are present for a substantial number of cancer types, unveiling them would amount to a major step forward in understanding cancer signature structure.

To address this question, in [16], we proposed a new clustering algorithm termed *K-means. Its basic building block is the vanilla k-means algorithm, which computationally is very inexpensive. However, it is also nondeterministic. *K-means uses two machine learning levels on top of k-means to achieve statistical determinism (see Section 2 for details) [18], without any initialization of the centers [19]. Once *K-means fixes the clustering, it turns out that the weights and exposures can be computed using (normalized) regressions [16], thereby altogether bypassing computationally-costly NMF. In [16], we applied this method to cancer genome data corresponding to 1389 published samples for 14 cancer types. We found that clustering works well for 10 out the 14 cancer types; the metrics include within-cluster correlations and overall fit quality. This suggests that there is indeed a clustering substructure present in the underlying cancer genome data, at least for most cancer types [20]. This is encouraging.

In this paper, we apply the method of [16] to exome data consisting of 10,656 published samples (sample IDs with sources are in Appendix A) aggregated by 32 cancer types. *K-means produces a robustly-stable clustering (11 clusters) from these data. One motivation for using exome data is that the exome is a small subset (∼1%) of the full genome containing only protein-coding regions of the genome [21]. The exome is much less expensive and less time consuming to sequence, which can be especially important for early-stage diagnostics, than the whole genome, yet it encodes important information about cancer signatures. As we discuss in the subsequent sections, our method appears to work well on exome data for most cancer types. In fact, overall, it appears to work better than COSMIC signatures, including out-of-sample, when applying clusters derived from our exome data to genome data.

## 2. *K-means

In [16], by extending a prior work [22] in quantitative finance on building statistical industry classifications using clustering algorithms, we developed a clustering method termed *K-means (“star K-means”) and applied it to the extraction of cancer signatures from genome data. *K-means is anchored on the standard k-means algorithm (see [23,24,25,26,27,28,29]) as its basic building block. However, k-means is not deterministic. *K-means is statistically deterministic, without specifying initial centers. This is achieved via two machine learning levels sitting on top of k-means. At the first level, we aggregate a large number *M* of k-means clusterings with randomly initialized centers (and the number of target clusters fixed using eRank) via a nontrivial aggregation procedure; see [16] for details. This aggregation is based on clustering (again, using k-means) the centers produced in the *M* clusterings, so the resultant aggregated clustering is nondeterministic. However, it is a lot less nondeterministic than vanilla k-means clusterings as aggregation dramatically reduces the degree of nondeterminism. At the second level, we take a large number *P* of such aggregated clusterings and determine the “ultimate” clustering with the maximum occurrence count (among the *P* aggregations). For sufficiently large *M* and *P*, the “ultimate” clustering is stable, i.e., if we run the algorithm over and over again, we will get the same “ultimate” clustering every time, even though the occurrence counts within different *P* aggregations are going to be different for various aggregations. What is important here is that the most frequently-occurring (“ultimate”) aggregation remains the same run after run. We emphasize that *K-means is a universal algorithm, and its application is not limited to the cancer genome or exome. We discuss how the input data (i.e., matrices of somatic mutation counts for cancer exome) are used in the context of *K-means in Section 3.2 (see [16] for technical details of *K-means).

## 3. Empirical Results

### 3.1. Data Summary

In this paper, we apply *K-means to exome data. (In [16], we applied it to published genome data. In this work, apart from applying *K-means to exome data, we also perform out-of-sample stability analysis of our results here (see Section 4).) We use data consisting of 10,656 published exome samples aggregated by 32 cancer types listed in Table 1, which summarizes total occurrence counts, numbers of samples and data sources. Appendix A provides sample IDs together with references for the data sources. Occurrence counts for the 96 mutation categories for each cancer type are given in Table A1, Table A2, Table A3 and Table A4. For Tables and Figures labeled A⋆, see Appendix A.

#### 3.1.1. Structure of the Data

The underlying data consist of matrices ${\left[G\left(s\right)\right]}_{i\mu (s)}$ whose elements are occurrence counts of mutation categories labeled by i=1,⋯,N=96 in samples labeled by μ(s)=1,⋯,d(s). Here, s=1,⋯,n labels *n* different cancer types (in our case n=32). We can choose to work with individual matrices ${\left[G\left(s\right)\right]}_{i\mu (s)}$ or with the $N\times d_{tot}$ “big matrix” Γ obtained by appending (i.e., bootstrapping) the matrices ${\left[G\left(s\right)\right]}_{i\mu (s)}$ together column-wise (so $d_{tot}=\sum_{s=1}^{n}d\left(s\right)$). Alternatively, we can aggregate samples by cancer types and work with the so-aggregated matrix:

(1) $$G_{is}=\sum_{\mu (s)=1}^{d(s)}{\left[G\left(s\right)\right]}_{i\mu (s)}$$

Generally, individual matrices ${\left[G\left(s\right)\right]}_{i\mu (s)}$ and, thereby, the “big matrix” Γ contain much noise. For some cancer types, we can have a relatively small number of samples. We can also have “sparsely-populated” data, i.e., with many zeros for some mutation categories. In fact, different samples are not even necessarily uniformly normalized. To mitigate the aforementioned issues, following [13], here, we work with the N×n matrix $G_{is}$ with samples aggregated by cancer types. Below, we apply *K-means to $G_{is}$.

### 3.2. Exome Data Results

The 96×32 matrix $G_{is}$ given in Table A1, Table A2, Table A3 and Table A4 is what we pass into the function bio.cl.sigs() in Appendix A of [16] as the input matrix x. We use: iter.max = 100 (this is the maximum number of iterations used in the built-in R function kmeans(); we note that there was not a single instance in our 30 million runs of kmeans() where more iterations were required – the R function kmeans() produces a warning if it does not converge within iter.max); num.try = 1000 (this is the number of individual k-means samplings we aggregate every time); and num.runs = 30,000 (which is the number of aggregated clusterings we use to determine the “ultimate”, that is the most frequently occurring, clustering). More precisely, we ran three batches with num.runs = 10,000 as a sanity check, to make sure that the final result based on 30,000 aggregated clusterings was consistent with the results based on smaller batches, i.e., that it was stable from batch to batch [30]. Based on Table A5, we identify Clustering-E1 as the “ultimate” clustering (see Section 2). Also, it is evident that the top-10 clusterings in Table A5 essentially are variations of each other.

**Figure 1:**
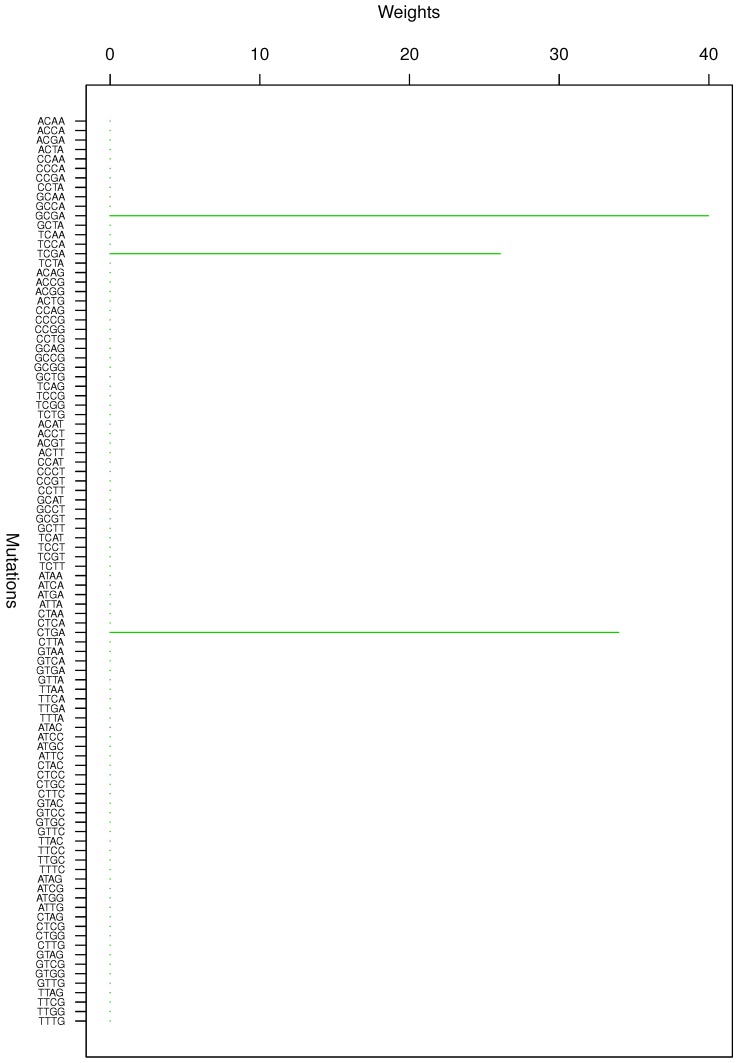
Cluster Cl-1 in Clustering-E1 with weights based on normalized regressions with arithmetic means.

**Figure 2:**
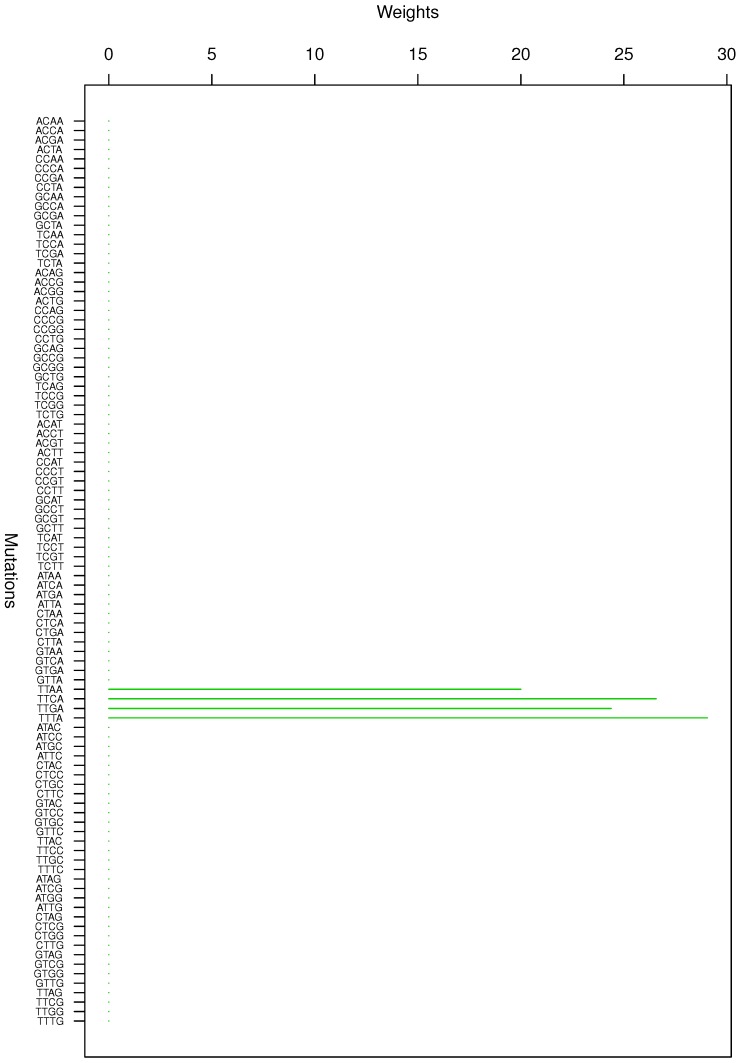
Cluster Cl-2 in Clustering-E1 with weights based on normalized regressions with arithmetic means.

**Figure 3:**
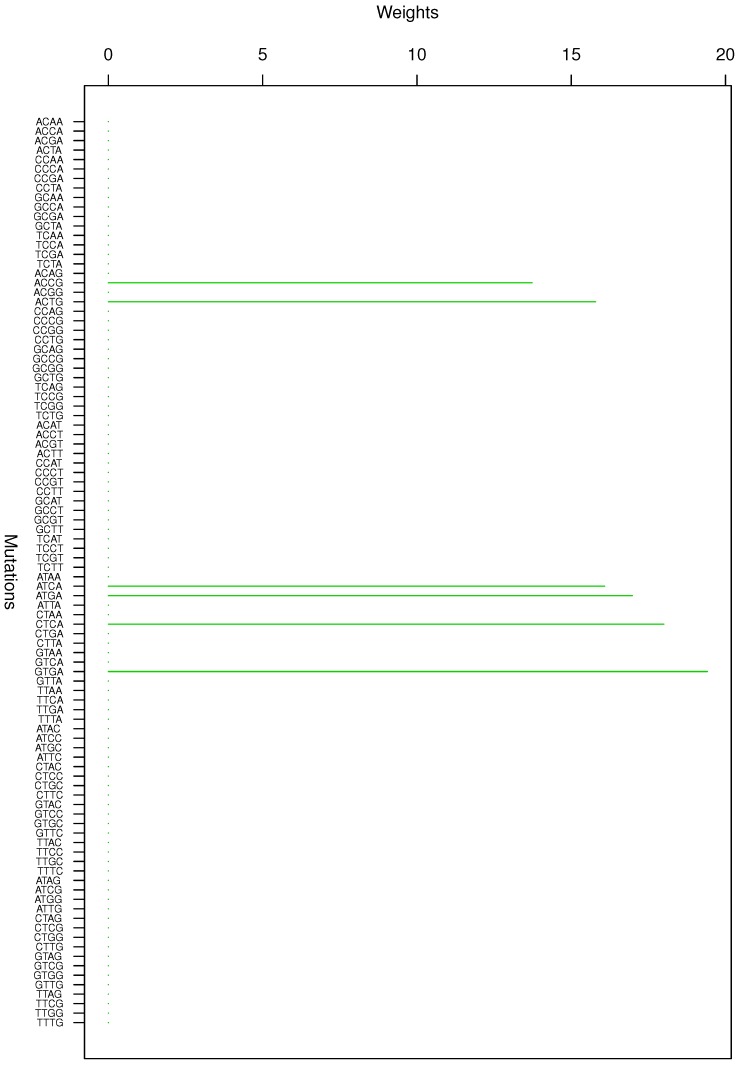
Cluster Cl-3 in Clustering-E1 with weights based on normalized regressions with arithmetic means.

**Figure 4:**
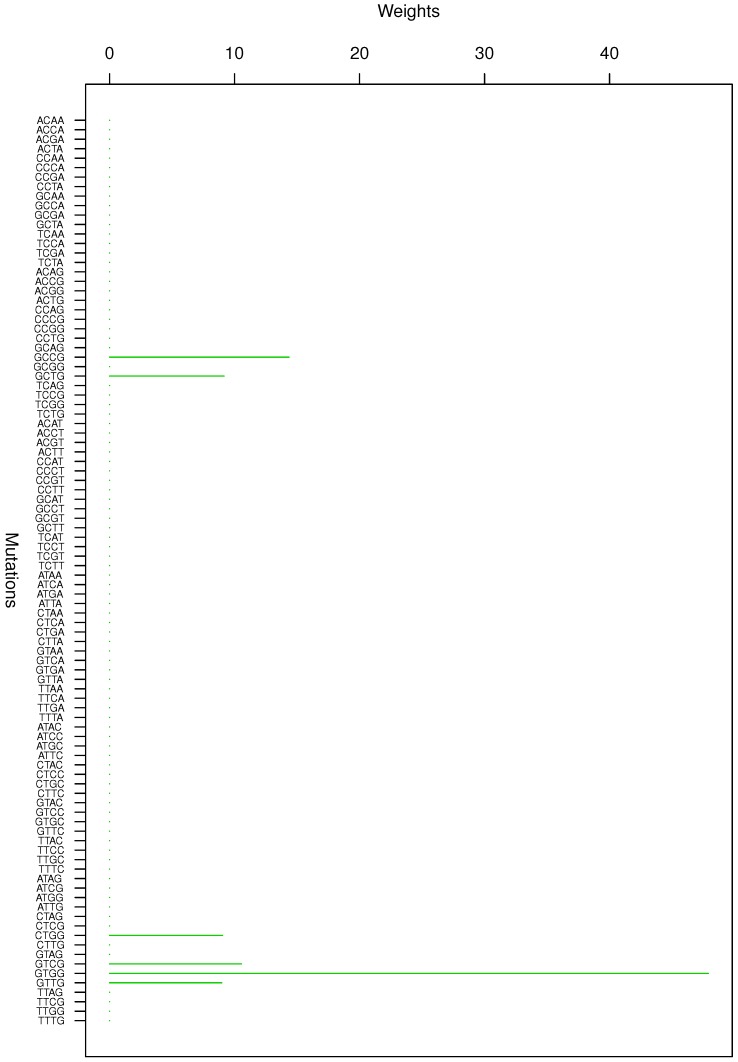
Cluster Cl-4 in Clustering-E1 with weights based on normalized regressions with arithmetic means.

**Figure 5:**
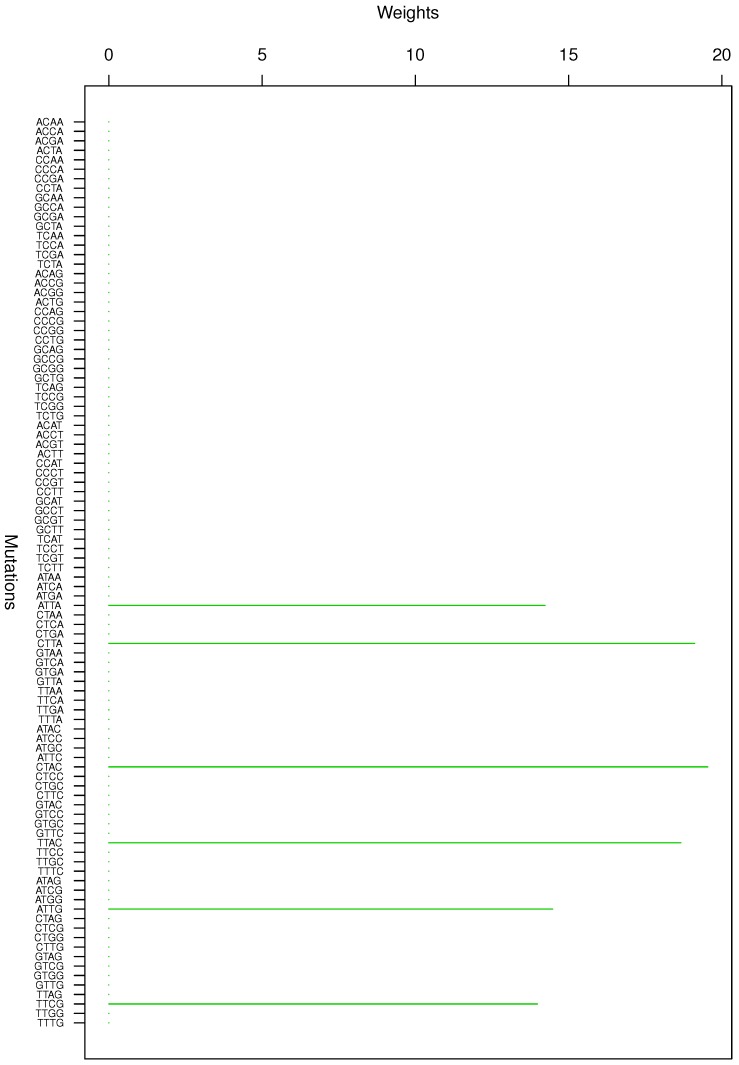
Cluster Cl-5 in Clustering-E1 with weights based on normalized regressions with arithmetic means.

**Figure 6:**
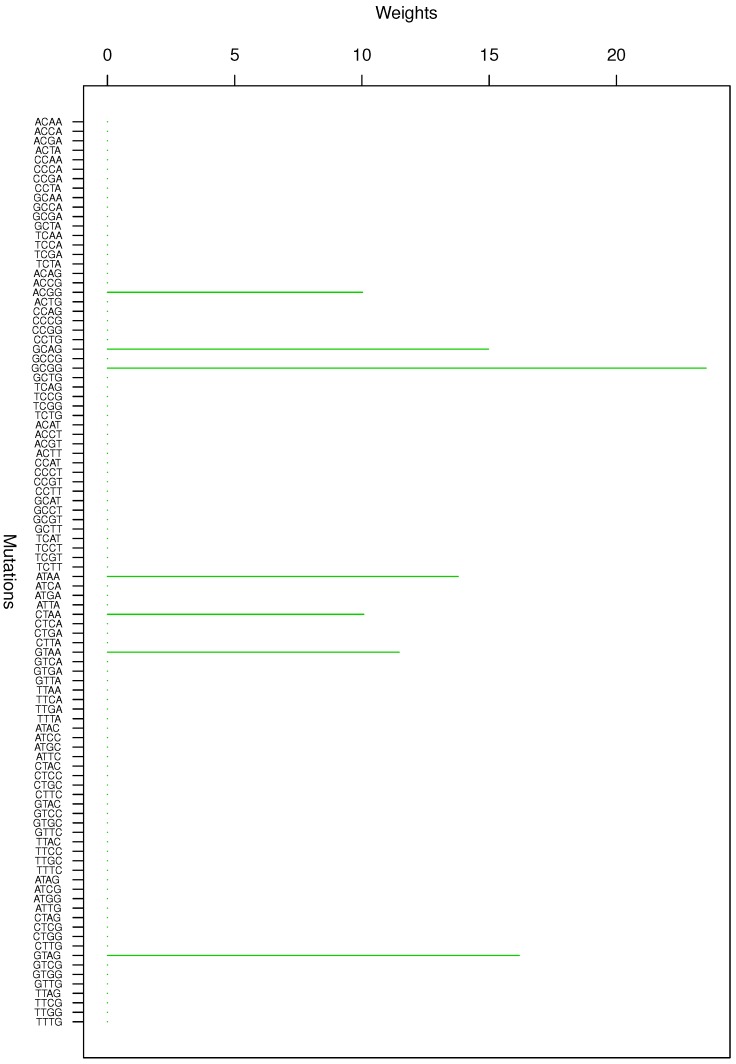
Cluster Cl-6 in Clustering-E1 with weights based on normalized regressions with arithmetic means.

**Figure 7:**
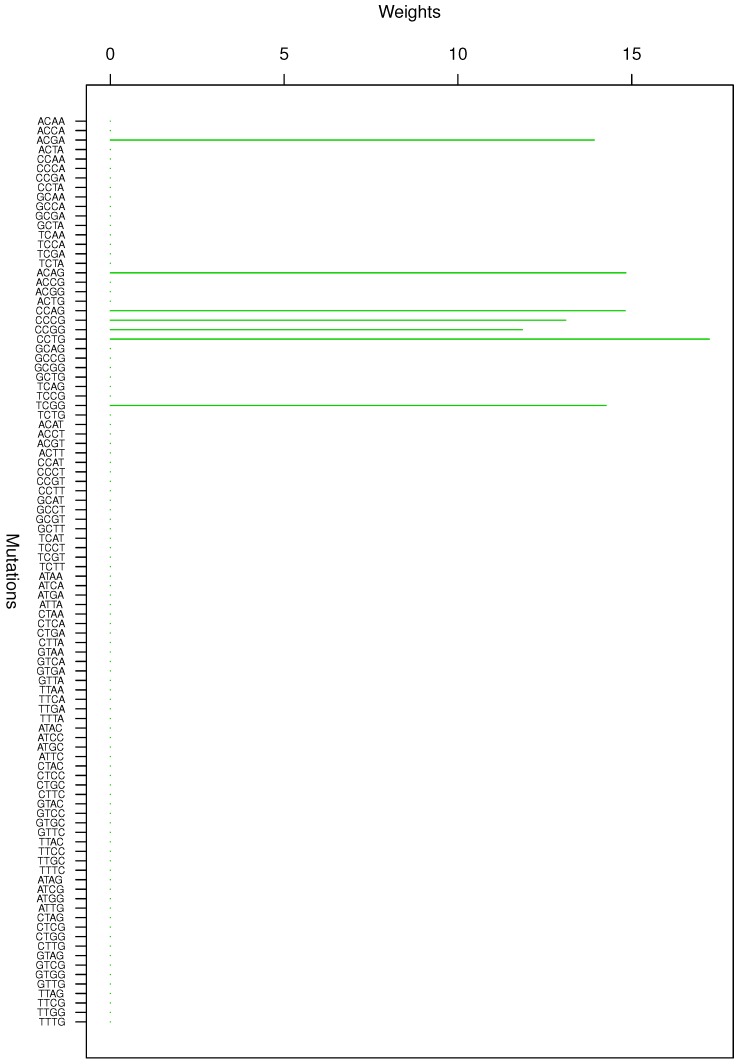
Cluster Cl-7 in Clustering-E1 with weights based on normalized regressions with arithmetic means.

**Figure 8:**
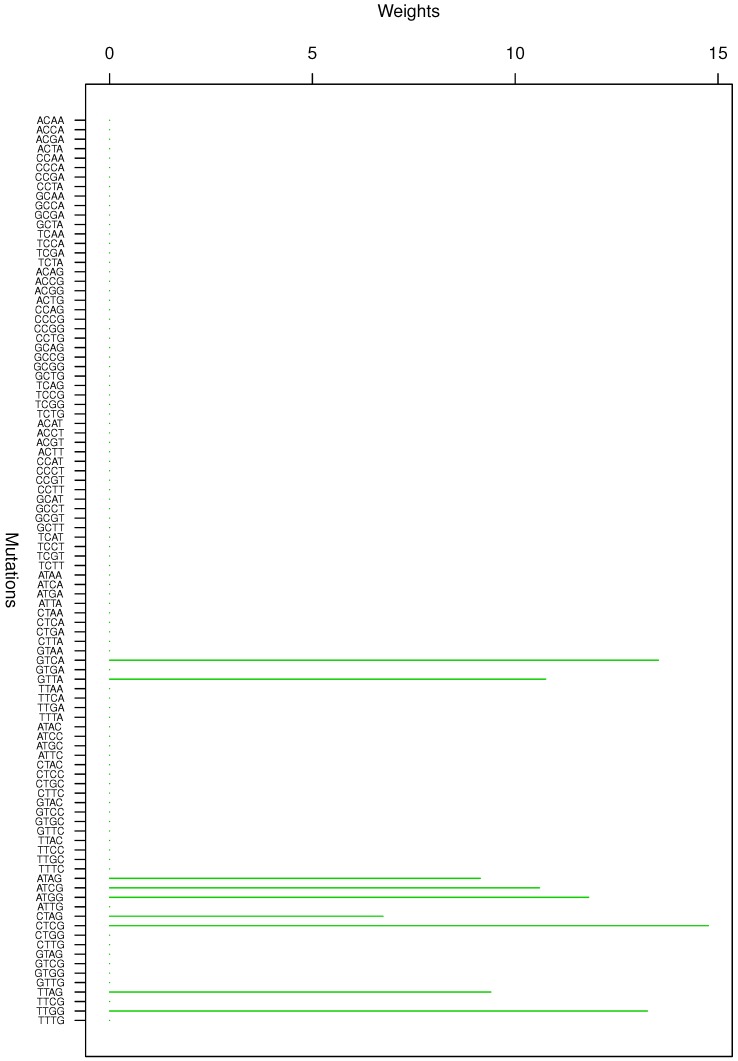
Cluster Cl-8 in Clustering-E1 with weights based on normalized regressions with arithmetic means.

**Figure 9:**
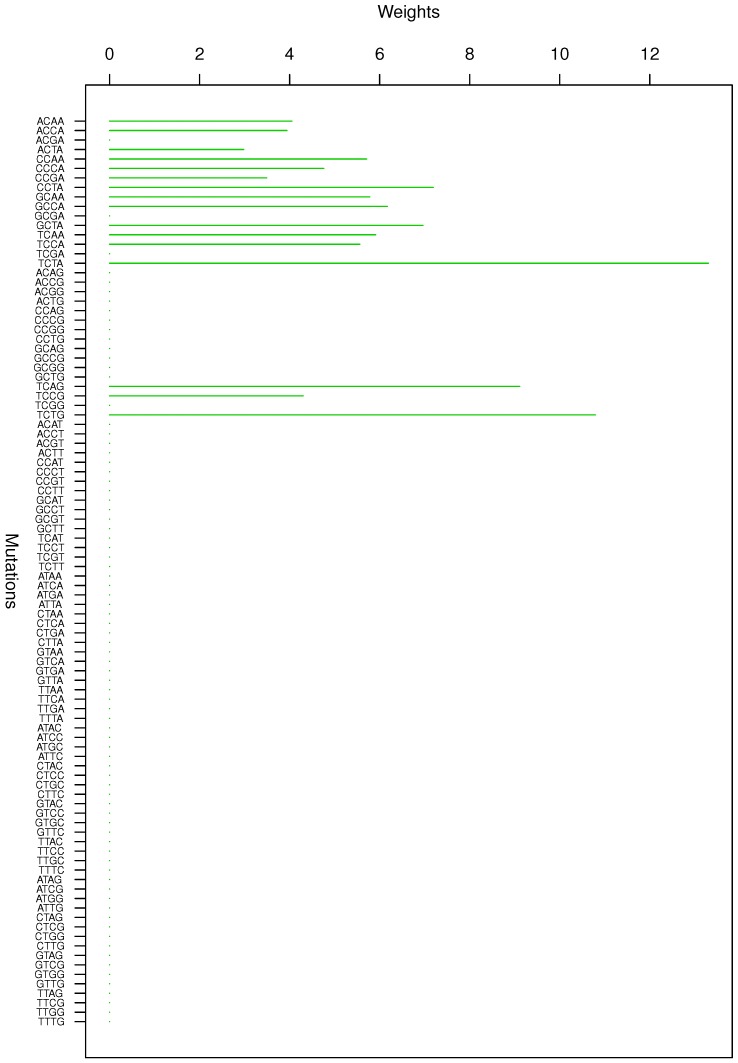
Cluster Cl-9 in Clustering-E1 with weights based on normalized regressions with arithmetic means.

**Figure 10:**
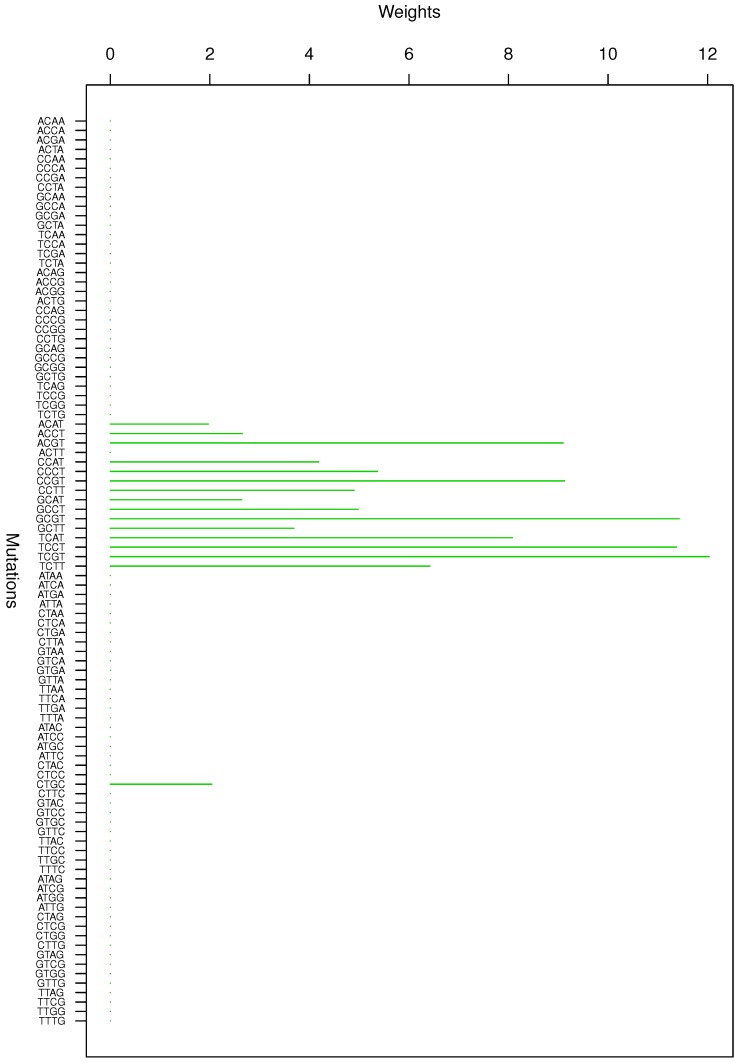
Cluster Cl-10 in Clustering-E1 with weights based on normalized regressions with arithmetic means.

**Figure 11:**
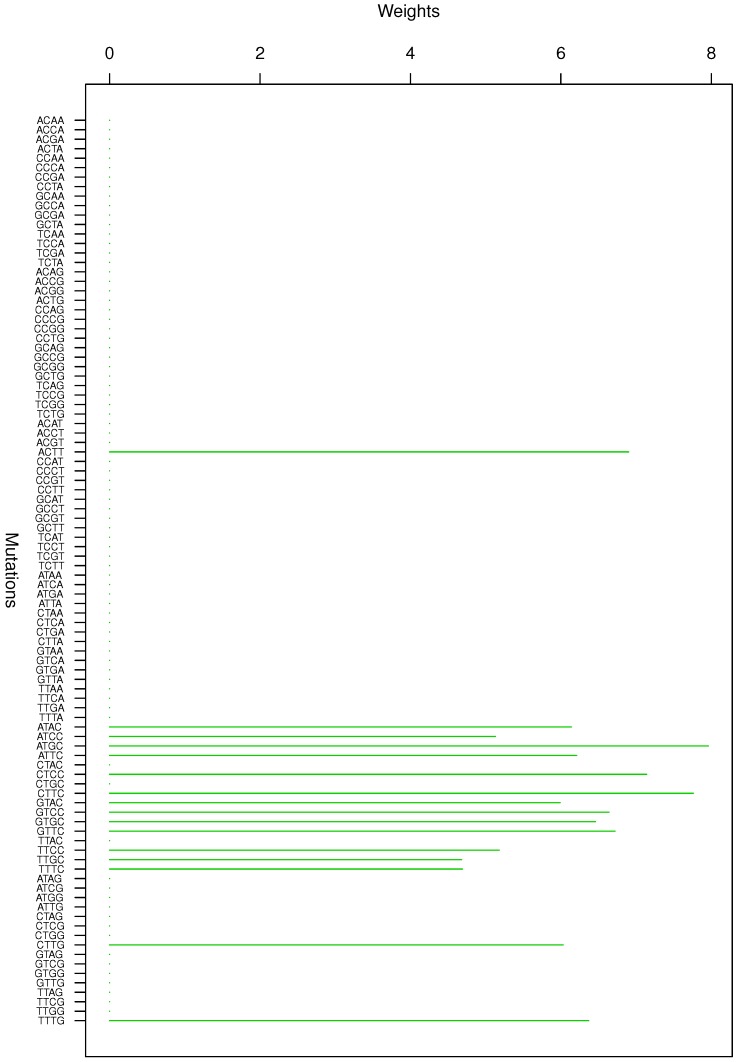
Cluster Cl-11 in Clustering-E1 with weights based on normalized regressions with arithmetic means.

**Figure A1:**
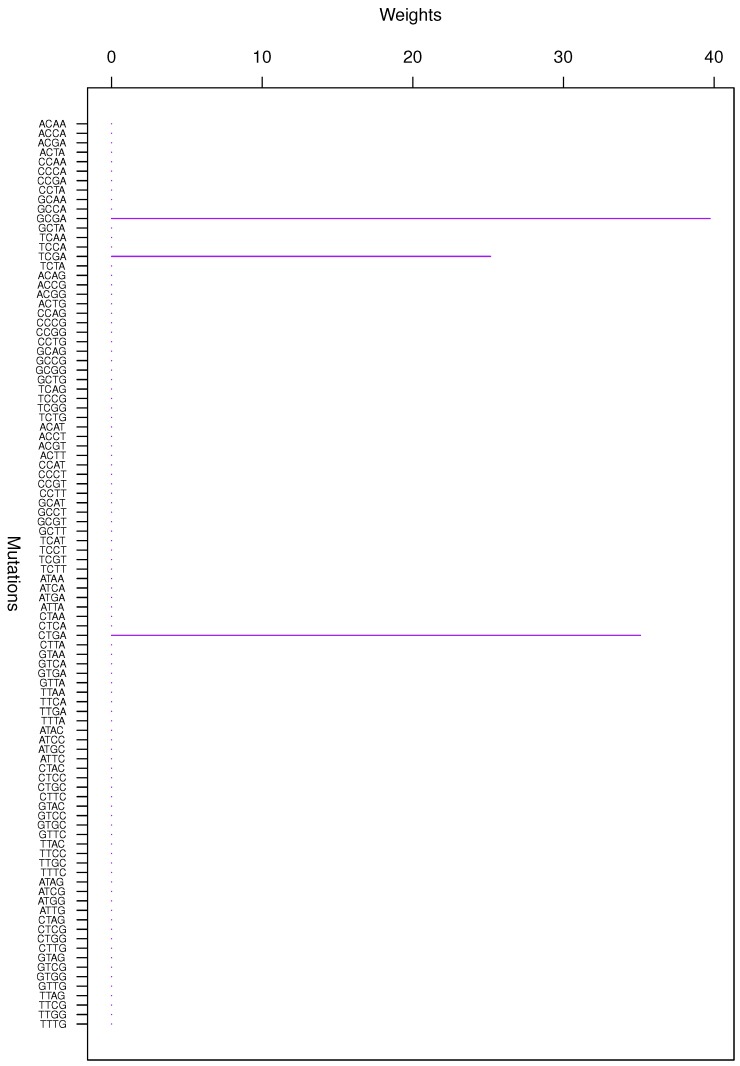
Cluster Cl-1 in Clustering-E1 with weights based on unnormalized regressions with arithmetic means.

**Figure A2:**
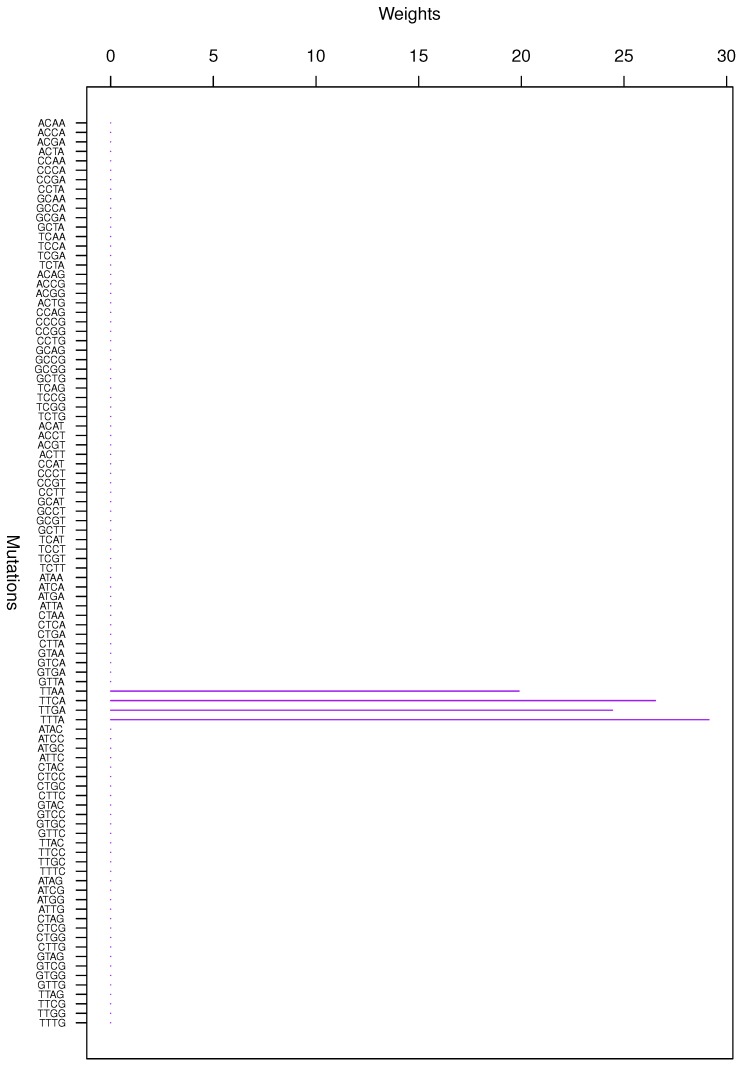
Cluster Cl-2 in Clustering-E1 with weights based on unnormalized regressions with arithmetic means.

**Figure A3:**
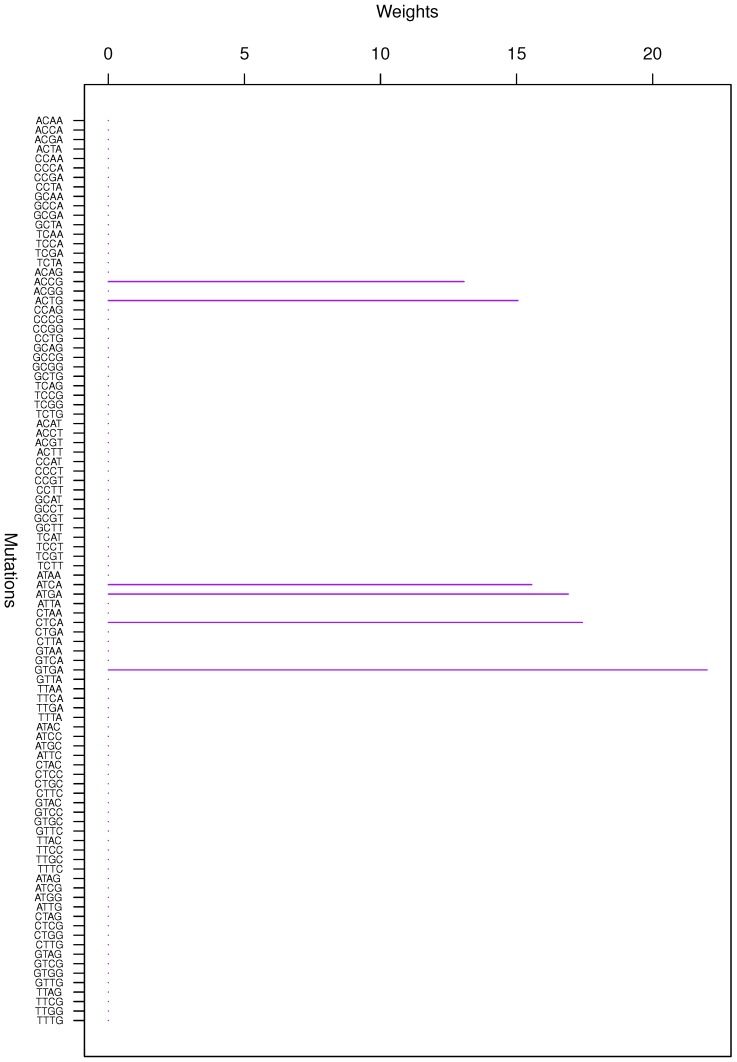
Cluster Cl-3 in Clustering-E1 with weights based on unnormalized regressions with arithmetic means.

**Figure A4:**
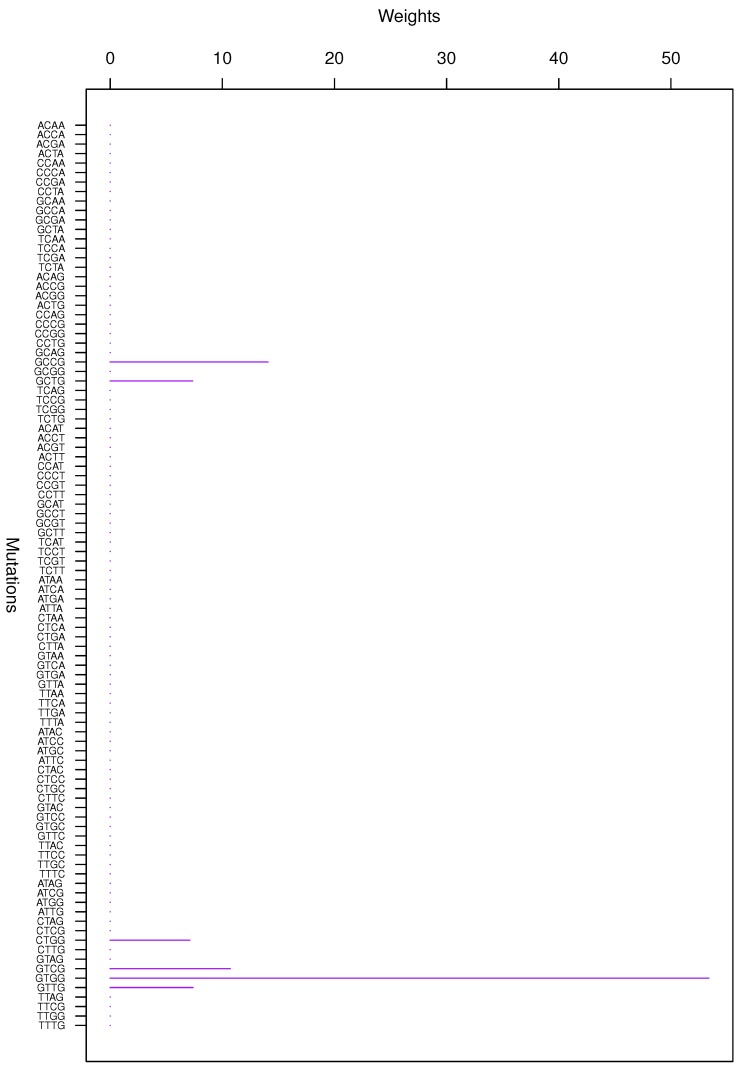
Cluster Cl-4 in Clustering-E1 with weights based on unnormalized regressions with arithmetic means.

**Figure A5:**
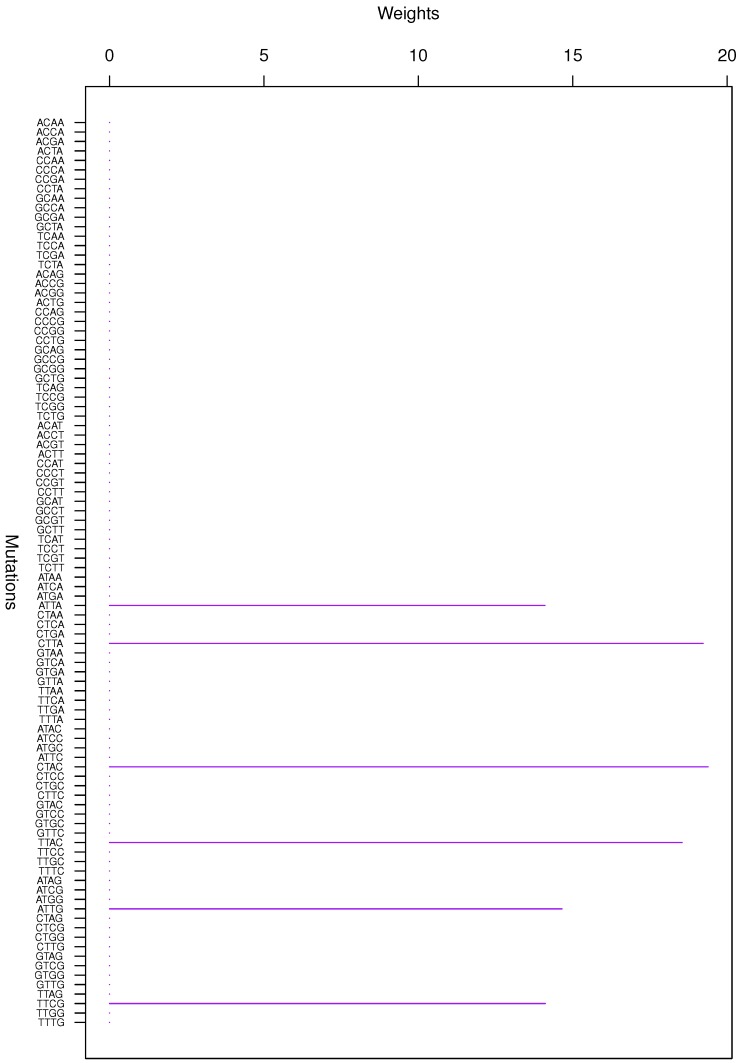
Cluster Cl-5 in Clustering-E1 with weights based on unnormalized regressions with arithmetic means.

**Figure A6:**
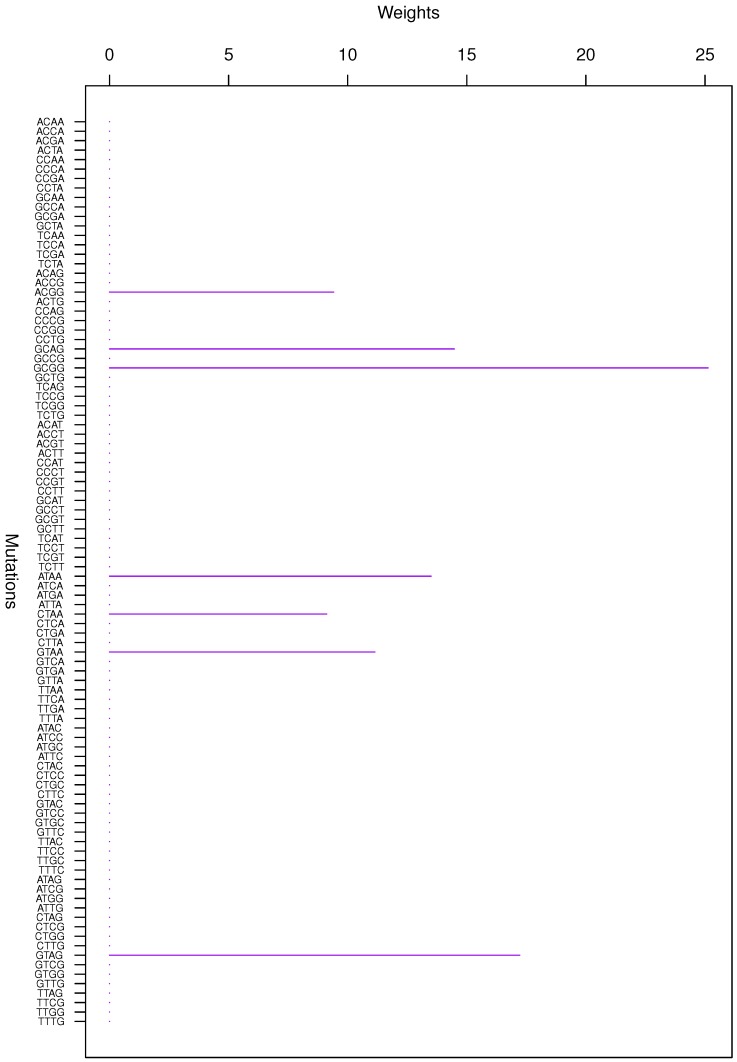
Cluster Cl-6 in Clustering-E1 with weights based on unnormalized regressions with arithmetic means.

**Figure A7:**
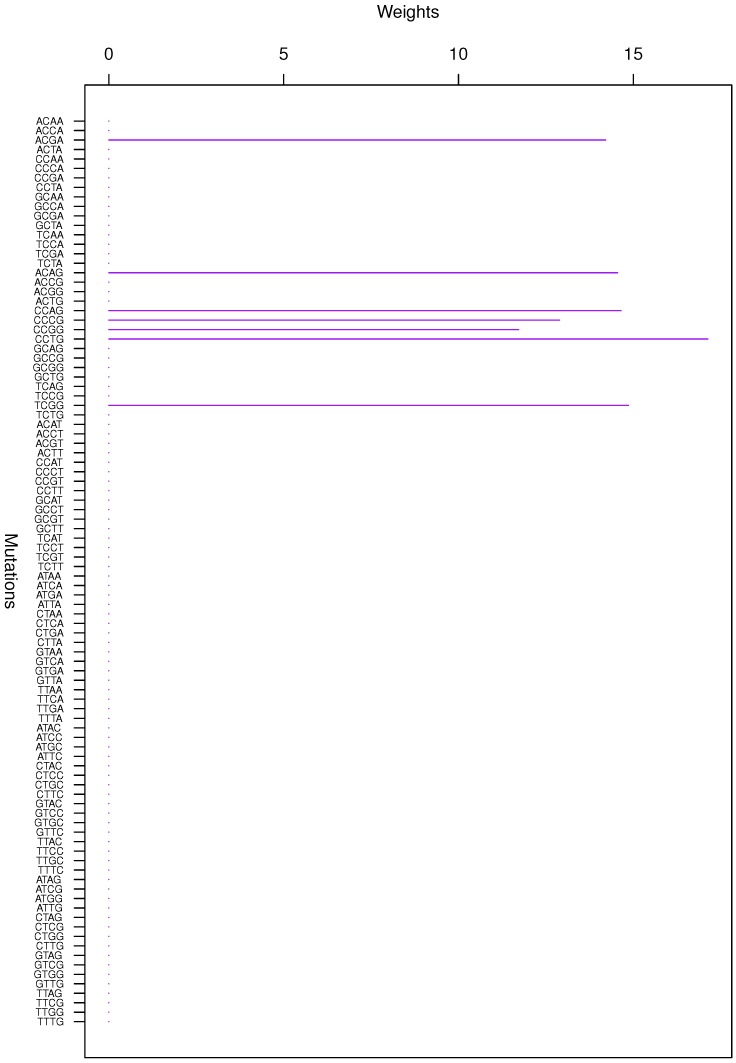
Cluster Cl-7 in Clustering-E1 with weights based on unnormalized regressions with arithmetic means.

**Figure A8:**
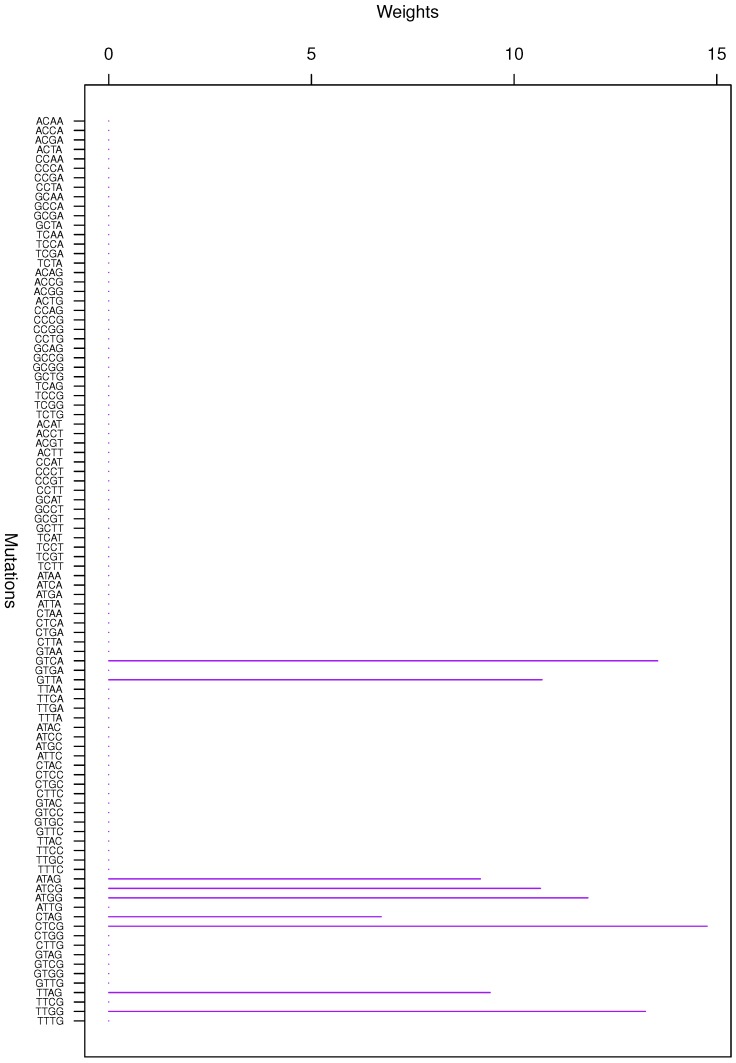
Cluster Cl-8 in Clustering-E1 with weights based on unnormalized regressions with arithmetic means.

**Figure A9:**
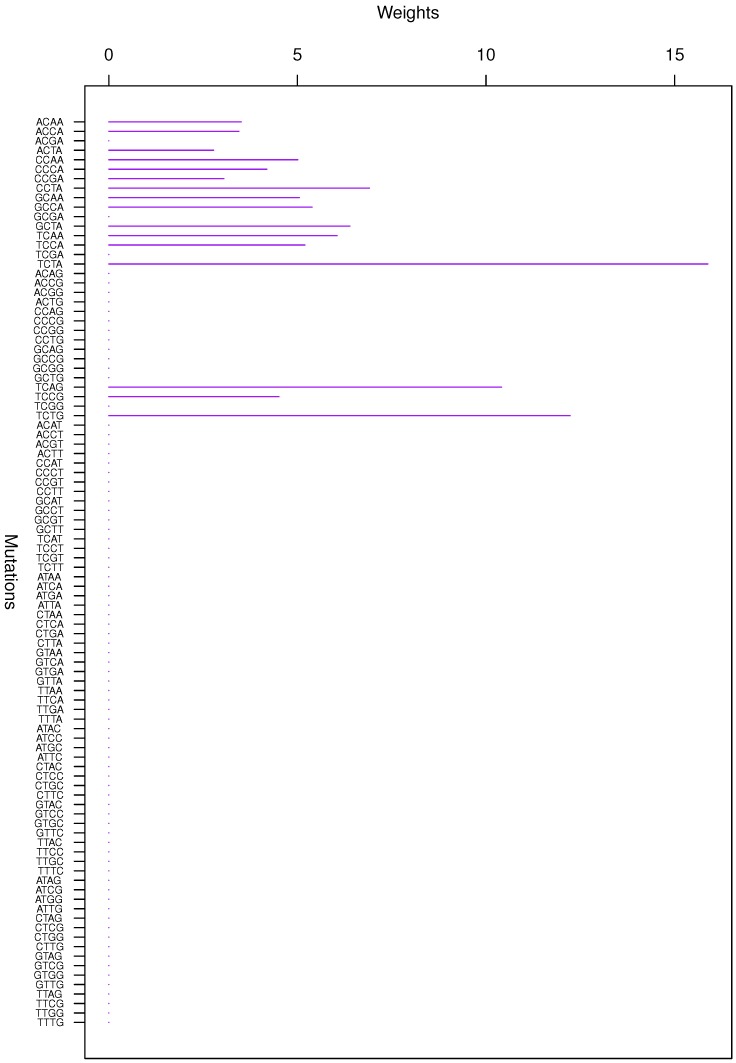
Cluster Cl-9 in Clustering-E1 with weights based on unnormalized regressions with arithmetic means.

**Figure A10:**
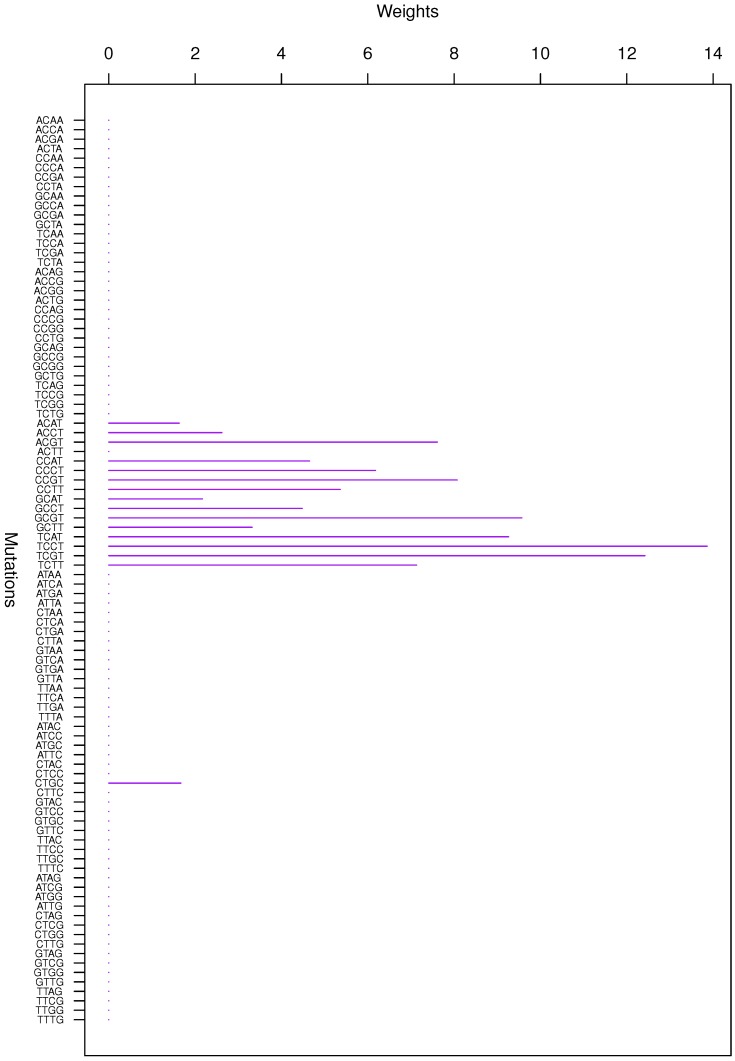
Cluster Cl-10 in Clustering-E1 with weights based on unnormalized regressions with arithmetic means.

**Figure A11:**
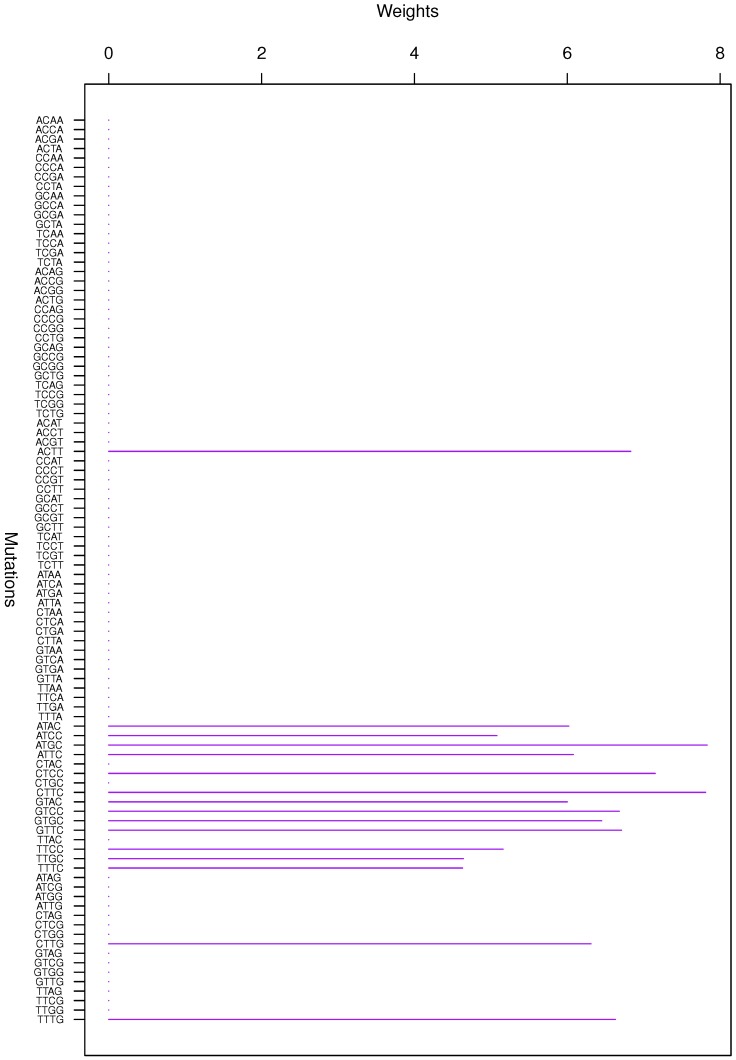
Cluster Cl-11 in Clustering-E1 with weights based on unnormalized regressions with arithmetic means.

For Clustering-E1, as in [16], we compute the within-cluster weights based on unnormalized regressions (via Equations (13)–(15) in [16]) and normalized regressions (via Equations (14), (16) and (17) in [16]) with exposures calculated based on arithmetic averages (see Section 2.6 of [16] for details). We give the within-cluster weights for Clustering-E1 in Table A6 and Table A7 and plot them in Figure A1, Figure A2, Figure A3, Figure A4, Figure A5, Figure A6, Figure A7, Figure A8, Figure A9, Figure A10 and Figure A11 for unnormalized regressions and in Table 2 and Table 3 and Figure 1, Figure 2, Figure 3, Figure 4, Figure 5, Figure 6, Figure 7, Figure 8, Figure 9, Figure 10 and Figure 11 for normalized regressions. The actual mutation categories in each cluster can be read off the aforesaid Table A6 and Table A7 with the weights (thus, the mutation categories with nonzero weights belong to a given cluster), or from the horizontal axis labels in the aforesaid Figure A1, Figure A2, Figure A3, Figure A4, Figure A5, Figure A6, Figure A7, Figure A8, Figure A9, Figure A10 and Figure A11.

### 3.3. Reconstruction and Correlations

#### 3.3.1. Within-Cluster Correlations

We have our data matrix $G_{is}$. We are approximating this matrix via the following factorized matrix:

(2) $$G_{is}^{*}=\sum_{A=1}^{K}W_{iA}\;H_{As}=w_{i}\;H_{Q(i),s}$$

where $W_{iA}$ are the within-cluster weights (i=1,⋯,N; A=1⋯,K), $H_{As}$ are the exposures (s=1,⋯,n=32 labels the cancer types), Q:{1,⋯,N}↦{1,⋯,K} is the map between the N=96 mutations and K=11 clusters in Clustering-E1, and we have $W_{iA}=w_{i}\;\delta_{Q(i),A}$ [31]. It is the matrix $W_{iA}$ that is given in Table A6 and Table A7 for the unnormalized regressions and Table 2 and Table 3 for the normalized regressions.

We can now compute an n×K matrix $\Theta_{sA}$ of within-cluster cross-sectional correlations between $G_{is}$ and $G_{is}^{*}$ defined via (xCor(·,·) stands for “cross-sectional correlation”, i.e., “correlation across the index *i*” – due to the factorized structure (2), these correlations do not directly depend on $H_{As}$)

(3) $$\Theta_{sA}={\left.\mathrm{xCor}(G_{is},G_{is}^{*})\right|}_{i\in J(A)}={\left.\mathrm{xCor}(G_{is},w_{i})\right|}_{i\in J(A)}$$

Here, J(A)={i|Q(i)=A} is the set of mutations labeled by *i* that belong to a given cluster labeled by *A*. We give the matrix $\Theta_{sA}$ for Clustering-E1 for weights based on unnormalized regressions in Table 4 and weights based on normalized regressions in Table 5. As for genome data [16], the fit for normalized regressions is somewhat better than that for unnormalized regressions.

#### 3.3.2. Overall Correlations

Another useful metric, which we use as a sanity check, is this. For each value of *s* (i.e., for each cancer type), we can run a linear cross-sectional regression (without the intercept) of $G_{is}$ over the matrix $W_{iA}$. Therefore, we have n=32 of these regressions. Each regression produces multiple $R^{2}$ and adjusted $R^{2}$, which we give in Table 4 and Table 5. Furthermore, we can compute the fitted values ${\hat{G}}_{is}^{*}$ based on these regressions, which are given by:

(4) $${\hat{G}}_{is}^{*}=\sum_{A=1}^{K}W_{iA}\;F_{As}=w_{i}\;F_{G(i),s}$$

where (for each value of *s*) $F_{As}$ are the regression coefficients. We can now compute the overall cross-sectional correlations (i.e., the index *i* runs over all N=96 mutation categories)

(5) $$\Xi_{s}=\mathrm{xCor}\left(G_{is},{\hat{G}}_{is}^{*}\right)$$

These correlations are also given in Table 4 and Table 5 and measure the overall fit quality.

#### 3.3.3. Interpretation

Looking at Table 5, a few things jump out. First, most—24 out of 32—cancer types have high (80%+) within-cluster correlations with at least one cluster. Out of the other eight cancer types, six have reasonably high (70%+) within-cluster correlations with at least one cluster. The remaining two cancer types are X9 (cervical cancer) and X17 (liver cancer). In [16], based on genome data, we already observed that liver cancer does not have a clustering structure, so this is not surprising. On the other hand, with cervical cancer, the story appears to be trickier. According to [17], we should expect COSMIC signatures CSig2+13 and CSig26 (see Section 4 for more details) to appear in cervical cancer. According to Table A8 (see Section 4), CSig2+13 indeed have high correlations with X9 (but not CSig26). On the other hand, the dominant part of CSig2 (C > T mutations in TCA, TCC, TCG, TCT) is subsumed in Cluster Cl-10 (see Figure 10), and the dominant part of CSig13 (C > G mutations in TCA, TCC, TCT) is subsumed in Cluster Cl-9 (see Figure 9). Basically, it appears that the large (each with 16 mutation categories) Clusters Cl-9, Cl-10 and Cl-11 probably could be split into smaller clusters. In fact, Cl-9 and Cl-11 do not have 80%+ correlations with any cancer types (they do have 70%+ correlations with one cancer type each). This is another indication that these clusters might be “oversized”. The same was observed with the largest cluster (with 21 mutation categories) in [16] in the context of genome data. Simply put, these “oversized” clusters may have to be dealt with via appropriately tweaking the underlying clustering algorithm (this is outside of the scope hereof and will be dealt with elsewhere).

The last three columns in Table 5 provide metrics for the overall fit for each cancer type. The overall correlations (between the original data $G_{is}$ and the model-fitted values ${\hat{G}}_{is}^{*}$; see Section 3.3.2) in the last column of Table 5 are above 80% for 16 (out of the 32) cancer types and above 70% for 26 cancer types. These high correlations indicate a good in-sample agreement between the original and reconstructed (model-fitted) data for each of these 26 cancer types. The remaining six cancer types, which all have overall correlations above 60%, are: X4 (B-cell lymphoma), X6 (bladder cancer), X8 (breast cancer), X9 (cervical cancer), X26 (rectum adenocarcinoma) and X29 (testicular germ cell tumor). We already discussed cervical cancer above. We address breast cancer in Section 4 hereof. Now, the X4 data are sparsely populated: there are 24 samples, and the total number of counts is 706, so there are many zeros in the underlying sample data, albeit only two zeros in the aggregated data. According to [17], we should expect CSig9 and CSig17 in B-cell lymphoma. However, according to Table A8 (see Section 4), these signatures do not have high correlations with X4. Note that clustering worked well for B-cell lymphoma for the genome data in [16], but there, the genome data were well-populated. Therefore, it is reasonable to assume that here, the “underperformance” is likely due to the sparsity of the underlying data. For X6 (bladder cancer), the situation is similar to X9 (cervical cancer) above: according to [17], we should expect CSig2+13 in bladder cancer, and Table A8 is consistent with this. However, as mentioned above, CSig2 and CSig13 are subsumed in Clusters Cl-10 and Cl-9, respectively (“oversizing”). According to Table A9, we should expect CSig10 in X26. CSig10 to be dominated by the C > A mutation in TCT (which is subsumed in Cluster Cl-9) and the C > T mutation in TCG (which is subsumed in Cluster Cl-10). Again, here we are dealing with “oversizing” of these clusters. X29 has high within-cluster correlations with Clusters Cl-4 and Cl-5. The overall fit correlation apparently is lowered by the high negative correlation with Cluster Cl-3. To summarize, “oversizing” is one potential “shortcoming” here.

## 4. Concluding Remarks

In order to understand the significance of our results, let us compare them to the fit that COSMIC signatures (for details, see [17]; for references, see [9,32,33,34,35]) provide for our exome data. We can do this by computing the following p×n cross-sectional correlation matrix:

(6) $$\Delta_{\alpha s}=\mathrm{xCor}\left(U_{i\alpha },G_{is}\right)$$

where $U_{i\alpha }$ (α=1,⋯,p) is the N×p matrix of weights for p=30 COSMIC signatures, which for brevity, we will refer to as CSig1, ..., CSig30 [36]. The matrix $\Delta_{\alpha s}$ is given in Table A8 and Table A9. Let us look at the 80%+ correlations (which are in bold font in Table A8 and Table A9). (Relaxing this cut-off to 70% (see Table A8 and Table A9) does not alter our conclusions below.) Only six out 30 COSMIC signatures, to wit CSig1,2,6,7,10,15, have 80%+ correlations with the exome data for the 32 cancer types. The aetiology of these signatures is known [17]. CSig1 is the result of an endogenous mutational process initiated by spontaneous 5-methylcytosine deamination, hence the ubiquity of its high correlations with many cancer types. CSig2 (which usually appears in tandem with CSig13) is due to APOBEC-mediated cytosine deamination, hence its high correlations with some cancer types. CSig6 is associated with defective DNA mismatch repair, hence its high correlations with several cancer types. CSig7 is due to ultraviolet light exposure, so its high correlation with X19 (melanoma) is spot on [37]. CSig10 is associated with recurrent error-prone polymerase POLE somatic mutations (its high correlations with X26 (rectum adenocarcinoma) and X32 (uterine cancer) are consistent with [17] and, once again, apparently are due to a large overlap between the exome data we use here and those used by [17]). CSig15 is associated with defective DNA mismatch repair; the significance of its high correlation with X23 (pancreatic cancer) is unclear. Therefore, only a handful of COSMIC signatures, all associated with known mutational processes, do well on our exome data [38]. Others do not fit well.

This is the out-of-sample stability issue emphasized in [13]. It traces to the fact that NMF is an intrinsically unstable method, both in- and out-of-sample. In-sample instability relates to the fact that NMF is nondeterministic and produces different looking signatures from one run to another. In fact, we attempted running NMF on our exome data. We ran three batches with 800 sampling in each batch (a computationally time-consuming procedure [39]). The three batches produced different looking results, which with much manual curation could only be partially matched to some COSMIC signatures, but this matching was different and highly unstable across the three batches. Simply put, NMF failed to produce any meaningful results on our exome data. Furthermore, the above discussion illustrates that most COSMIC signatures (extracted using NMF from exome and genome data) apparently are unstable out-of-sample, e.g., when applied to our exome data aggregated by cancer types. Here, one may argue that exome data contain only partial information, and NMF should not be used on it. However, the COSMIC signatures are in fact based on 10,952 exomes and 1048 whole-genomes across 40 cancer types [17] (also, see, e.g., [40]). The difference here is that we are aggregating samples by cancer types, and most COSMIC signatures apparently do not apply, which means that COSMIC signatures are highly sample-set-specific (that is, unstable out-of-sample). Furthermore, as mentioned above, CSig7 (UV exposure) is spot on in that it has 99.66% correlation with X19 (melanoma) (albeit one should keep in mind the comments in [37]). Therefore, one can argue that the culprit is not the exome data, but the method (NMF) itself. To quantify this, let us look at correlations of COSMIC signatures with genome data for 14 cancer types used in [13] and [16]. The results are given in Table A10. As in the case of exome data, here too, we have high correlations only for a handful of COSMIC signatures corresponding to known mutational processes, to wit CSig1,4,6,13. Therefore, most COSMIC signatures do not appear to have explanatory power on genome data aggregated by cancer types, a further indication that most COSMIC signatures lack out-of-sample stability.

What about out-of-sample stability for our clusters we obtained from exome data? One way to test this is to look at within-cluster correlations and the overall fit metrics as in Table 5, but for the aforesaid genome data for 14 cancer types used in [13,16]. The results are given in Table 6. Unsurprisingly, the quality of the fit for genome data (out-of-sample) is not as good as for exome data (in-sample). However, it is (i) reasonably good and (ii) unequivocally much better than the fit provided by the COSMIC signatures (Table A10). Furthermore, the 11 exome-based clusters have a poor overall fit for G.X4 (breast cancer), G.X8 (liver cancer), G.X9 (lung cancer) and G.X14 (renal cell carcinoma), the same four cancer types for which seven genome-based clusters in [16] produced a poor overall fit, and for a good reason as well (see [16] for details). It is less clear why the 11 exome-based clusters do not have a better fit for G.X7 (gastric cancer) considering the in-sample fits for this cancer type based on exome data (X15; Table 5 hereof) and genome data (Row 7, Table 15 of [16]) are petty good.

Therefore, unlike NMF, *K-means clustering, being a statistically deterministic method, is in-sample stable. Here, we can ask, what if we apply to NMF the same two machine learning levels as those that sit on top of k-means in *K-means to make it statistically deterministic? The answer is that when applying NMF, one already uses one machine learning method, which is a form of aggregation of a large number of samplings (i.e., individual NMF runs) [41]. This is conceptually similar to the first machine learning level in *K-means. Therefore, then we can ask, what if we add to NMF the second machine learning level as in *K-means, to wit by comparing a large number of such “averagings”? A simple, prosaic answer is that it would make NMF, which is already computationally costly as is and much more so with the first machine learning level, computationally prohibitive. The reason why *K-means is computationally much less expensive is that the basic building block of *K-means, on top of which we add the two machine learning methods, is vanilla k-means, which is much, much less expensive than NMF. That is what makes all the difference [42].

Finally, let us mention that exome data for chronic myeloid disorders (121 samples, 175 total counts) were published in [43,44], and for neuroblastoma (13 samples, 298 total counts) in [45]. However, these data are so sparsely populated (too many zeros even after aggregation) that we specifically excluded them from our analysis. Much more unpublished data are available for the cancer types we analyze here, as well as other cancer types, and it would be very interesting to apply our methods to these data, including to (still embargoed) extensive genome data of the International Cancer Genome Consortium.

## Figures and Tables

**Table 1:** Exome data summary. See Appendix A for the data source definitions. Here, we label cancer types via X1–X32 for use in the tables below.

Label	Cancer Type	Total Counts	# of Samples	Source
X1	Acute Lymphoblastic Leukemia	938	86	H1, Z1, D1
X2	Acute Myeloid Leukemia	1414	190	T1
X3	Adrenocortical Carcinoma	11,530	91	T2
X4	B-Cell Lymphoma	706	24	M1, L1
X5	Benign Liver Tumor	884	40	P1
X6	Bladder Cancer	90,121	341	G1, T3
X7	Brain Lower Grade Glioma	38,041	465	T4
X8	Breast Cancer	201,555	1182	N1, S1, S2, T5
X9	Cervical Cancer	47,715	197	T6
X10	Cholangiocarcinoma	12,156	139	Z2, T7
X11	Chronic Lymphocytic Leukemia	975	80	Q1
X12	Colorectal Cancer	214,814	581	S3, T8
X13	Esophageal Cancer	59,088	329	D2, T9
X14	Gastric Cancer	161,078	401	Z3, W1, T10
X15	Glioblastoma Multiforme	23,230	359	P2, T11
X16	Head and Neck Cancer	96,816	591	A1, S4, T12
X17	Liver Cancer	252,755	452	S5, H2, T13
X18	Lung Cancer	306,071	1018	D3, R1, P3, S6, I1, T14
X19	Melanoma	357,060	594	S7, D4, B1, A2, H3, T15
X20	Nasopharyngeal Cancer	2241	11	L2
X21	Oral Cancer	13,462	106	I2
X22	Ovarian Cancer	20,610	471	J1, T16
X23	Pancreatic Cancer	39,788	184	W2, J2, T17
X24	Pheochromocytoma and Paraganglioma	3709	178	T18
X25	Prostate Cancer	22,808	480	B2, B3, G2, T19
X26	Rectum Adenocarcinoma	32,797	115	T20
X27	Renal Cell Carcinoma	47,635	709	G3, T21
X28	Sarcoma	28,256	255	T22
X29	Testicular Germ Cell Tumor	6064	150	T23
X30	Thymoma	4444	123	T24
X31	Thyroid Carcinoma	6833	409	T25
X32	Uterine Cancer	164,211	305	T26
—	All Cancer Types	2,269,805	10,656	Above

**Table 2:** Weights for the first 48 mutation categories for the 11 clusters in Clustering-E1 (see Table A5) based on normalized regressions (see Section 3.2 for details). The conventions are the same as in Table A6.

Mutation	Cl-1	Cl-2	Cl-3	Cl-4	Cl-5	Cl-6	Cl-7	Cl-8	Cl-9	Cl-10	Cl-11
ACAA	0.00	0.00	0.00	0.00	0.00	0.00	0.00	0.00	4.05	0.00	0.00
ACCA	0.00	0.00	0.00	0.00	0.00	0.00	0.00	0.00	3.94	0.00	0.00
ACGA	0.00	0.00	0.00	0.00	0.00	0.00	13.92	0.00	0.00	0.00	0.00
ACTA	0.00	0.00	0.00	0.00	0.00	0.00	0.00	0.00	2.98	0.00	0.00
CCAA	0.00	0.00	0.00	0.00	0.00	0.00	0.00	0.00	5.71	0.00	0.00
CCCA	0.00	0.00	0.00	0.00	0.00	0.00	0.00	0.00	4.76	0.00	0.00
CCGA	0.00	0.00	0.00	0.00	0.00	0.00	0.00	0.00	3.49	0.00	0.00
CCTA	0.00	0.00	0.00	0.00	0.00	0.00	0.00	0.00	7.19	0.00	0.00
GCAA	0.00	0.00	0.00	0.00	0.00	0.00	0.00	0.00	5.78	0.00	0.00
GCCA	0.00	0.00	0.00	0.00	0.00	0.00	0.00	0.00	6.17	0.00	0.00
GCGA	39.97	0.00	0.00	0.00	0.00	0.00	0.00	0.00	0.00	0.00	0.00
GCTA	0.00	0.00	0.00	0.00	0.00	0.00	0.00	0.00	6.96	0.00	0.00
TCAA	0.00	0.00	0.00	0.00	0.00	0.00	0.00	0.00	5.91	0.00	0.00
TCCA	0.00	0.00	0.00	0.00	0.00	0.00	0.00	0.00	5.56	0.00	0.00
TCGA	26.06	0.00	0.00	0.00	0.00	0.00	0.00	0.00	0.00	0.00	0.00
TCTA	0.00	0.00	0.00	0.00	0.00	0.00	0.00	0.00	13.30	0.00	0.00
ACAG	0.00	0.00	0.00	0.00	0.00	0.00	14.83	0.00	0.00	0.00	0.00
ACCG	0.00	0.00	13.73	0.00	0.00	0.00	0.00	0.00	0.00	0.00	0.00
ACGG	0.00	0.00	0.00	0.00	0.00	10.02	0.00	0.00	0.00	0.00	0.00
ACTG	0.00	0.00	15.79	0.00	0.00	0.00	0.00	0.00	0.00	0.00	0.00
CCAG	0.00	0.00	0.00	0.00	0.00	0.00	14.81	0.00	0.00	0.00	0.00
CCCG	0.00	0.00	0.00	0.00	0.00	0.00	13.10	0.00	0.00	0.00	0.00
CCGG	0.00	0.00	0.00	0.00	0.00	0.00	11.85	0.00	0.00	0.00	0.00
CCTG	0.00	0.00	0.00	0.00	0.00	0.00	17.23	0.00	0.00	0.00	0.00
GCAG	0.00	0.00	0.00	0.00	0.00	14.97	0.00	0.00	0.00	0.00	0.00
GCCG	0.00	0.00	0.00	14.36	0.00	0.00	0.00	0.00	0.00	0.00	0.00
GCGG	0.00	0.00	0.00	0.00	0.00	23.52	0.00	0.00	0.00	0.00	0.00
GCTG	0.00	0.00	0.00	9.16	0.00	0.00	0.00	0.00	0.00	0.00	0.00
TCAG	0.00	0.00	0.00	0.00	0.00	0.00	0.00	0.00	9.11	0.00	0.00
TCCG	0.00	0.00	0.00	0.00	0.00	0.00	0.00	0.00	4.30	0.00	0.00
TCGG	0.00	0.00	0.00	0.00	0.00	0.00	14.26	0.00	0.00	0.00	0.00
TCTG	0.00	0.00	0.00	0.00	0.00	0.00	0.00	0.00	10.79	0.00	0.00
ACAT	0.00	0.00	0.00	0.00	0.00	0.00	0.00	0.00	0.00	1.97	0.00
ACCT	0.00	0.00	0.00	0.00	0.00	0.00	0.00	0.00	0.00	2.65	0.00
ACGT	0.00	0.00	0.00	0.00	0.00	0.00	0.00	0.00	0.00	9.10	0.00
ACTT	0.00	0.00	0.00	0.00	0.00	0.00	0.00	0.00	0.00	0.00	6.90
CCAT	0.00	0.00	0.00	0.00	0.00	0.00	0.00	0.00	0.00	4.19	0.00
CCCT	0.00	0.00	0.00	0.00	0.00	0.00	0.00	0.00	0.00	5.37	0.00
CCGT	0.00	0.00	0.00	0.00	0.00	0.00	0.00	0.00	0.00	9.13	0.00
CCTT	0.00	0.00	0.00	0.00	0.00	0.00	0.00	0.00	0.00	4.90	0.00
GCAT	0.00	0.00	0.00	0.00	0.00	0.00	0.00	0.00	0.00	2.64	0.00
GCCT	0.00	0.00	0.00	0.00	0.00	0.00	0.00	0.00	0.00	4.98	0.00
GCGT	0.00	0.00	0.00	0.00	0.00	0.00	0.00	0.00	0.00	11.43	0.00
GCTT	0.00	0.00	0.00	0.00	0.00	0.00	0.00	0.00	0.00	3.69	0.00
TCAT	0.00	0.00	0.00	0.00	0.00	0.00	0.00	0.00	0.00	8.08	0.00
TCCT	0.00	0.00	0.00	0.00	0.00	0.00	0.00	0.00	0.00	11.38	0.00
TCGT	0.00	0.00	0.00	0.00	0.00	0.00	0.00	0.00	0.00	12.03	0.00
TCTT	0.00	0.00	0.00	0.00	0.00	0.00	0.00	0.00	0.00	6.42	0.00

**Table 3:** Table 2, continued: weights for the next 48 mutation categories.

Mutation	Cl-1	Cl-2	Cl-3	Cl-4	Cl-5	Cl-6	Cl-7	Cl-8	Cl-9	Cl-10	Cl-11
ATAA	0.00	0.00	0.00	0.00	0.00	13.78	0.00	0.00	0.00	0.00	0.00
ATCA	0.00	0.00	16.08	0.00	0.00	0.00	0.00	0.00	0.00	0.00	0.00
ATGA	0.00	0.00	16.98	0.00	0.00	0.00	0.00	0.00	0.00	0.00	0.00
ATTA	0.00	0.00	0.00	0.00	14.23	0.00	0.00	0.00	0.00	0.00	0.00
CTAA	0.00	0.00	0.00	0.00	0.00	10.07	0.00	0.00	0.00	0.00	0.00
CTCA	0.00	0.00	18.00	0.00	0.00	0.00	0.00	0.00	0.00	0.00	0.00
CTGA	33.97	0.00	0.00	0.00	0.00	0.00	0.00	0.00	0.00	0.00	0.00
CTTA	0.00	0.00	0.00	0.00	19.11	0.00	0.00	0.00	0.00	0.00	0.00
GTAA	0.00	0.00	0.00	0.00	0.00	11.46	0.00	0.00	0.00	0.00	0.00
GTCA	0.00	0.00	0.00	0.00	0.00	0.00	0.00	13.53	0.00	0.00	0.00
GTGA	0.00	0.00	19.41	0.00	0.00	0.00	0.00	0.00	0.00	0.00	0.00
GTTA	0.00	0.00	0.00	0.00	0.00	0.00	0.00	10.75	0.00	0.00	0.00
TTAA	0.00	20.00	0.00	0.00	0.00	0.00	0.00	0.00	0.00	0.00	0.00
TTCA	0.00	26.57	0.00	0.00	0.00	0.00	0.00	0.00	0.00	0.00	0.00
TTGA	0.00	24.38	0.00	0.00	0.00	0.00	0.00	0.00	0.00	0.00	0.00
TTTA	0.00	29.05	0.00	0.00	0.00	0.00	0.00	0.00	0.00	0.00	0.00
ATAC	0.00	0.00	0.00	0.00	0.00	0.00	0.00	0.00	0.00	0.00	6.14
ATCC	0.00	0.00	0.00	0.00	0.00	0.00	0.00	0.00	0.00	0.00	5.13
ATGC	0.00	0.00	0.00	0.00	0.00	0.00	0.00	0.00	0.00	0.00	7.96
ATTC	0.00	0.00	0.00	0.00	0.00	0.00	0.00	0.00	0.00	0.00	6.21
CTAC	0.00	0.00	0.00	0.00	19.54	0.00	0.00	0.00	0.00	0.00	0.00
CTCC	0.00	0.00	0.00	0.00	0.00	0.00	0.00	0.00	0.00	0.00	7.14
CTGC	0.00	0.00	0.00	0.00	0.00	0.00	0.00	0.00	0.00	2.04	0.00
CTTC	0.00	0.00	0.00	0.00	0.00	0.00	0.00	0.00	0.00	0.00	7.76
GTAC	0.00	0.00	0.00	0.00	0.00	0.00	0.00	0.00	0.00	0.00	5.99
GTCC	0.00	0.00	0.00	0.00	0.00	0.00	0.00	0.00	0.00	0.00	6.64
GTGC	0.00	0.00	0.00	0.00	0.00	0.00	0.00	0.00	0.00	0.00	6.46
GTTC	0.00	0.00	0.00	0.00	0.00	0.00	0.00	0.00	0.00	0.00	6.72
TTAC	0.00	0.00	0.00	0.00	18.66	0.00	0.00	0.00	0.00	0.00	0.00
TTCC	0.00	0.00	0.00	0.00	0.00	0.00	0.00	0.00	0.00	0.00	5.18
TTGC	0.00	0.00	0.00	0.00	0.00	0.00	0.00	0.00	0.00	0.00	4.68
TTTC	0.00	0.00	0.00	0.00	0.00	0.00	0.00	0.00	0.00	0.00	4.69
ATAG	0.00	0.00	0.00	0.00	0.00	0.00	0.00	9.14	0.00	0.00	0.00
ATCG	0.00	0.00	0.00	0.00	0.00	0.00	0.00	10.60	0.00	0.00	0.00
ATGG	0.00	0.00	0.00	0.00	0.00	0.00	0.00	11.81	0.00	0.00	0.00
ATTG	0.00	0.00	0.00	0.00	14.48	0.00	0.00	0.00	0.00	0.00	0.00
CTAG	0.00	0.00	0.00	0.00	0.00	0.00	0.00	6.74	0.00	0.00	0.00
CTCG	0.00	0.00	0.00	0.00	0.00	0.00	0.00	14.76	0.00	0.00	0.00
CTGG	0.00	0.00	0.00	9.04	0.00	0.00	0.00	0.00	0.00	0.00	0.00
CTTG	0.00	0.00	0.00	0.00	0.00	0.00	0.00	0.00	0.00	0.00	6.03
GTAG	0.00	0.00	0.00	0.00	0.00	16.18	0.00	0.00	0.00	0.00	0.00
GTCG	0.00	0.00	0.00	10.55	0.00	0.00	0.00	0.00	0.00	0.00	0.00
GTGG	0.00	0.00	0.00	47.90	0.00	0.00	0.00	0.00	0.00	0.00	0.00
GTTG	0.00	0.00	0.00	8.98	0.00	0.00	0.00	0.00	0.00	0.00	0.00
TTAG	0.00	0.00	0.00	0.00	0.00	0.00	0.00	9.40	0.00	0.00	0.00
TTCG	0.00	0.00	0.00	0.00	13.98	0.00	0.00	0.00	0.00	0.00	0.00
TTGG	0.00	0.00	0.00	0.00	0.00	0.00	0.00	13.26	0.00	0.00	0.00
TTTG	0.00	0.00	0.00	0.00	0.00	0.00	0.00	0.00	0.00	0.00	6.37

**Table 4:** The within-cluster cross-sectional correlations $\Theta_{sA}$ (Columns 2–12), the overall correlations $\Xi_{s}$ (Column 15) based on the overall cross-sectional regressions and multiple $R^{2}$ and adjusted $R^{2}$ of these regressions (Columns 13 and 14). The cluster weights are based on unnormalized regressions (see Section 3.2 and Section 3.3.1 for details). All quantities are in the units of 1% rounded to 2 digits. The values above 80% are given in bold font. The values above 70% are underlined.

Type	Cl-1	Cl-2	Cl-3	Cl-4	Cl-5	Cl-6	Cl-7	Cl-8	Cl-9	Cl-10	Cl-11	$R^{2}$	adj-$R^{2}$	Cor
X1	**82.73**	52.61	−38.35	**88.99**	−2.48	62.31	44.74	47.99	46.96	68.29	−17.3	**81.53**	79.14	**86.03**
X2	57.84	79.57	−21.35	2.46	14.07	29	−23.27	45.7	−9.06	37.86	23.69	61.55	56.57	70.97
X3	**97.84**	59.33	−34.88	**85.84**	**93.71**	20.49	49.36	72.28	24.43	48.92	13.55	75.84	72.71	75.03
X4	79.67	9.54	2.33	−53.43	33.46	−25.78	−10.98	37.47	49.42	35.11	6.69	70.35	66.51	60.11
X5	**99.21**	36.43	13.54	46.65	**96.99**	−30.2	−76.87	51.58	−27.23	18.49	37.03	70.96	67.21	61.21
X6	−87.79	64.06	−30.37	**93.94**	**89.43**	27.25	41.11	**81.67**	66.06	61.77	57.68	64.09	59.44	74.35
X7	49.56	**94.33**	−63.27	23.6	69.48	−4.55	53.97	**88.73**	45.59	34.97	28.95	59.28	54.01	68
X8	−33.14	16.16	−72.5	**97.72**	36.79	−35.38	76.35	58.44	67.06	51.8	44.57	65.49	61.02	72.05
X9	−94.76	61.06	−88.86	−49.91	−5.3	−20.18	30.47	59.55	64.49	62.66	37.67	61.25	56.24	73.38
X10	30.52	−7.31	75.57	7.44	77.24	−53.34	36.34	**82.27**	10.41	42.52	54.56	74.72	71.45	65.04
X11	6.48	54.54	53.91	−58.77	44.42	−0.39	70.02	−30.55	49.29	42.58	28.14	77.46	74.55	72.98
X12	−72.76	76.02	−15.94	−43.69	7.61	−44.71	37.64	64.45	67.75	47.08	50.24	67.32	63.09	73.76
X13	−85.31	**93.35**	−52.58	−40.15	50.94	11.64	**93.66**	76.36	73.57	58.14	31.23	73.99	70.63	76.38
X14	70.94	62.01	−32.58	−42.79	**85.81**	−31.98	69.19	77.94	31.37	35.25	38.43	55.44	49.67	65.84
X15	12.1	**87.76**	−64.16	62.01	**92.19**	−40.36	56.64	77.94	34.37	39.43	46.34	60.01	54.84	70.44
X16	30.62	**83.56**	−8.44	−1.16	**84.79**	1	71.1	69.7	60.24	**80.7**	37.18	**85.79**	**83.95**	**87.99**
X17	45.65	1.38	8.66	23.53	66.13	−13.07	45.08	40	17.91	8.63	23.89	75.75	72.62	65.94
X18	66.52	9.2	79.62	−13.51	78.2	−5.95	16.95	56.68	8.34	65.83	51.17	76.87	73.88	74.51
X19	−56.72	76.08	41.95	77.89	21.98	−24.67	−44.45	69.91	−6.06	69.84	31.03	70.19	66.33	**81.77**
X20	63.1	−45.68	59.23	**99.95**	71.77	**98.3**	−66.7	**94.37**	−19.75	54.91	20.7	**91.03**	**89.87**	**94.01**
X21	30.55	−9.19	−3.43	32.96	58.57	−42.26	18.66	10.7	5.66	**87.75**	43.01	78.2	75.37	**81.7**
X22	14.3	**89.91**	−48.97	−15.32	41.05	−28.35	45.06	77.4	49.01	57.61	46.71	**82.71**	**80.48**	73.91
X23	−94.6	78.61	−10.88	54.36	−54.29	−25.86	**80.35**	79.53	41.25	36.57	56.69	59.87	54.68	69.15
X24	14.36	17.95	−64.97	−6.44	67.95	3.25	68.4	77.5	33.97	30.86	8.48	69.67	65.74	71.2
X25	**99.22**	−10.4	−68.67	31.16	70.04	−30.46	51.85	**88.25**	39.8	42.09	38.82	65.17	60.66	70.86
X26	−99.86	68.28	−42.04	−91.74	−44.57	41.96	−32.18	−17.2	71.07	59.33	12.88	51.13	44.8	67.02
X27	22.46	77.37	−17.66	60.25	67.81	−54.02	54.78	**81**	43.57	45.36	69.68	**81.75**	79.39	71.86
X28	74.8	**86.01**	−20.06	20.25	52.18	−29.64	60.87	**82.46**	−0.92	**87.42**	56.07	74.64	71.36	78.39
X29	56.6	−32.03	−73.41	**86.76**	**89.79**	5.85	45.1	65.9	−19.74	8.92	48.8	63.01	58.22	52.27
X30	53.68	**89.56**	−8.73	59.42	27.29	14.29	14.57	55.29	−34.73	35.97	50.84	63.94	59.27	66.09
X31	−63.1	**94.6**	**86.58**	−25.37	54.44	−4.68	23.57	70.77	45.75	58.82	45.69	**80.63**	78.12	**81.63**
X32	−90.38	**92.13**	−40.68	−46.9	−41.39	−47.56	29.25	28.35	70.58	53.25	14.12	60.58	55.47	71.25

**Table 5:** The within-cluster cross-sectional correlations $\Theta_{sA}$ (Columns 2–12), the overall correlations $\Xi_{s}$ (Column 15) based on the overall cross-sectional regressions and multiple $R^{2}$ and adjusted $R^{2}$ of these regressions (Columns 13 and 14). The cluster weights are based on normalized regressions (see Section 3.2 and Section 3.3.1 for details). All quantities are in the units of 1% rounded to 2 digits. The values above 80% are given in bold font. The values above 70% are underlined.

Type	Cl-1	Cl-2	Cl-3	Cl-4	Cl-5	Cl-6	Cl-7	Cl-8	Cl-9	Cl-10	Cl-11	$R^{2}$	adj-$R^{2}$	Cor
X1	74.82	52.72	−43.94	**90.19**	1.85	65.36	45.69	47.77	51.21	**84.05**	−13.31	**89.21**	**87.81**	**92.03**
X2	46.86	79.8	−0.94	3.68	14.82	30.35	−20.14	46.18	−6.97	59.59	32.36	72.73	69.2	**80.3**
X3	**99.69**	59.47	−9.32	**86.89**	**93.76**	26.13	50.46	72.39	34.47	70.55	23.15	**85.24**	**83.34**	**85.05**
X4	71.23	10.76	15.04	−51.44	30.56	−21.88	1.98	36.41	47.97	55.7	−1.58	75.85	72.72	67.89
X5	**100**	35.83	30.3	49.26	**98.08**	−28.3	−65.45	51.98	−17.43	38.98	44.82	77.99	75.14	70.79
X6	−93.22	63.8	−16.06	**94.84**	**90.84**	31.67	27.56	**82.12**	61.61	53.05	64.31	58.83	53.5	69.75
X7	37.97	**94.43**	−47.29	25.4	69.11	0.83	63.68	**88.6**	48.09	59.4	38.15	70.76	66.97	78.2
X8	−45.01	16.5	−62.63	**98.28**	39.35	−32.27	**83.6**	59.19	63.11	43.4	50.52	61.19	56.17	67.77
X9	−89.86	62.25	−80.98	−47.45	−8.97	−16.74	16.12	59.39	60.3	55	33.34	56.23	50.57	69.13
X10	18.02	−8.2	**82.63**	10.18	74.99	−52.09	41.77	**82.73**	20.02	63.78	58.84	79.37	76.7	71.53
X11	−6.42	55.44	52.93	−57.54	47.63	−1.28	**81.7**	−30.07	50.66	62.3	33.12	**84.34**	**82.31**	**81.56**
X12	−63.33	76.58	4.49	−41.77	7.54	−42.03	48.89	64.25	66.89	68.73	45.58	74.94	71.69	**80.69**
X13	−77.89	**93.71**	−27.8	−38.68	47.29	17.44	**95.87**	76.87	75.5	77.51	23.68	**81.52**	79.13	**83.69**
X14	61.29	63.15	−18.3	−41.27	**87.58**	−28.2	**81.25**	78.09	36.86	60.05	33.54	67.85	63.69	76.76
X15	−0.78	**87.99**	−55.94	63.9	**93.15**	−35.35	70.96	78.38	33.18	61.53	54.21	71.73	68.07	**80.1**
X16	18.11	**84.31**	19.73	0.91	**85.33**	7.48	60.17	70.2	57.35	76.08	45.64	**84.17**	**82.13**	**86.47**
X17	33.82	1.05	30.67	26.74	69.21	−9.94	58.67	39.91	22.7	29.39	32.72	**80.42**	77.89	72.46
X18	56.36	8.18	**80.84**	−11.72	77.47	0.12	14.08	57.32	14.3	55.78	58.66	**81.32**	78.91	79.27
X19	−66.84	76.03	63.58	79.35	25.45	−22.45	−33.4	70.1	−5.23	47.49	31.47	57.05	51.49	72.24
X20	72.56	−45.53	50.76	**99.76**	74.67	**96.7**	−64.71	**94.63**	−15.57	67	21.85	**90.27**	**89.01**	**93.47**
X21	42.55	−8.08	−21.72	35.24	61.4	−38.89	10.53	10.83	17.15	**92.87**	48.29	**84.49**	**82.49**	**87.07**
X22	1.45	**90.5**	−35.62	−13.13	39.71	−23.66	60.34	77.98	48.6	72.1	53.62	**87.25**	**85.6**	**80.31**
X23	−89.64	79.52	16.97	55.42	−56.06	−22.53	**84.97**	79.22	45.96	59.23	56.69	70.93	67.17	78.71
X24	1.5	19.38	−60	−5.03	71.17	9.51	76.81	77.96	32.31	54.11	18.55	79.13	76.43	**80.84**
X25	**96.78**	−9.27	−60.49	33.73	71.27	−27.25	66.72	**88.29**	45.16	65.44	47.19	76.9	73.91	**81.59**
X26	−98.33	68.09	−18.47	−91.28	−47.48	47.37	−21.32	−17.49	68.11	70.34	4.83	52.9	46.8	68.69
X27	9.74	78.26	3.2	62.7	67.15	−51.82	65.98	**80.74**	48.21	65.36	74.89	**87.38**	**85.74**	**80.4**
X28	**82.72**	**86.77**	3.18	23.06	54.14	−24.21	69.04	**82.83**	8.5	**88.99**	61.88	77.71	74.83	**81.01**
X29	66.74	−30.7	−75.41	**86.26**	**90.39**	6.19	40.05	65.75	−9.15	33.08	50	70.78	67	63.15
X30	64.09	**89.73**	−9.78	59.19	24.42	15.12	10.09	54.47	−29.33	60	55.91	74.91	71.66	77.47
X31	−72.56	**94.15**	68.86	−23.61	56.73	−0.07	29.76	71.5	42.07	75.55	52.13	**85.8**	**83.96**	**86.69**
X32	−84.12	**92.48**	−23.26	−45.55	−42.69	−46.15	27.16	27.83	68.89	68.65	6.59	62.67	57.84	73.22

**Table 6:** The within-cluster cross-sectional correlations $\Theta_{sA}$ (Columns 2–12), the overall correlations $\Xi_{s}$ (Column 15) based on the overall cross-sectional regressions and multiple $R^{2}$ and adjusted $R^{2}$ of these regressions (Columns 13 and 14). The cluster weights are based on normalized regressions (see Section 3.2 and Section 3.3.1 for details). The definitions of cancer types G.X1–G.X14 for genome data are given in Table A10. All quantities are in the units of 1% rounded to 2 digits. The values above 80% are given in bold font. The values above 70% are underlined.

Type	Cl-1	Cl-2	Cl-3	Cl-4	Cl-5	Cl-6	Cl-7	Cl-8	Cl-9	Cl-10	Cl-11	$R^{2}$	adj-$R^{2}$	Cor
G.X1	8.47	−42.14	−5.83	−28.47	8.6	−27.81	68.1	−58.76	78.71	43.09	1.98	76.74	73.73	64.82
G.X2	12.58	−8.39	5.78	−17.48	36.44	−39.46	65.49	−12.25	32.07	52.76	18.76	**80.04**	77.46	74.6
G.X3	7.9	17.51	−12.85	37.46	63.86	−48.79	43.86	40.63	20.77	53.57	10.21	78.96	76.24	79.3
G.X4	−7.33	−4.67	−35.67	**90.16**	27.48	−35.7	76.34	21.87	59.39	29.95	18.74	57.38	51.87	60.47
G.X5	8.64	−2.63	4.76	13.57	18.72	−19.86	48.29	−54.11	38.75	38.46	5.22	**80.96**	78.49	66.43
G.X6	19.29	**86.79**	63.27	−26.72	−1.53	−52.18	**83.96**	34.55	69.9	77.08	56.94	**83.66**	**81.54**	**86.8**
G.X7	0.1	15.21	40.26	−28.56	3.03	−38.4	60.42	63.09	56.62	42.03	9.24	68.45	64.37	62.44
G.X8	58.39	25.87	−1.42	−17.3	−83.31	75.44	−68.56	65.93	−23.25	−27.49	17.47	58.65	53.3	9.81
G.X9	28.73	−62.34	77.8	64.57	**87.7**	−47.09	49.71	17.1	15.13	3.02	29.05	76.2	73.12	69.99
G.X10	−20.84	−15.96	−61.68	24.6	17.53	−33.44	39.85	−5.4	34.93	58.49	4.99	78.48	75.7	78.18
G.X11	7.25	39.51	−7.86	44.23	46	−54.88	67.08	25.45	50.2	41.11	15.02	**83.99**	**81.92**	65.21
G.X12	7.9	−88.83	−70.05	21.84	**90.47**	−23.28	66.92	53.73	59.49	67.2	70.4	73.55	70.12	**81.42**
G.X13	−5.33	−30.41	−61.53	−56.72	−11.91	−37.94	64.53	−20.61	60.22	66.73	−4.61	**84.31**	**82.28**	79.24
G.X14	6.33	−39.94	42.62	−21	56.7	−51.19	65.01	7	26.19	5.52	−12.27	71.58	67.9	39.79

## Appendix A: Exome Sample IDs

In this Appendix, we give the sample IDs with the corresponding publication references for the exome data we use. We label these references as H1, Z1, etc., and use these labels in Table 1 in the Sources column. This appendix also includes Tables and Figures labeled A⋆ (Table A1, Table A2, Table A3, Table A4, Table A5, Table A6, Table A7, Table A8,Table A9 ,Table A10, Table A11 and Figure A1, Figure A2, Figure A3, Figure A4, Figure A5, Figure A6, Figure A7, Figure A8, Figure A9, Figure A10, Figure A11); see Section 3.1.

⧫ **Acute Lymphoblastic Leukemia** (86 samples):
• Source H1 = [46]. Sample IDs are of the form SJHYPO*, where * is:
  001-D, 002-D, 004-D, 005-D, 006-D, 009-D, 009-R, 012-D, 013-D, 014-D, 016-D, 019-D, 020-D, 022-D, 024-D, 026-D, 029-D, 032-D, 036-D, 037-D, 037-R, 039-D, 040-D, 041-D, 042-D, 044-D, 045-D, 046-D, 047-D, 051-D, 052-D, 052-R, 055-D, 056-D, 116-D, 117-D, 119-D, 120-D, 123-D, 124-D, 125-D, 126-D.
Source Z1 = [47]. Sample IDs are of the form SJTALL*, where * is:
  001, 002, 003, 004, 005, 006, 007, 008, 009, 011, 012, 013, 169, 192, 208.
• Source D1 = [48]:
  TBR01, TBR03, TBR05, TBR06, TBR08, TLE02, TLE10, TLE109, TLE31, TLE33, TLE34, TLE38, TLE39, TLE41, TLE42, TLE43, TLE50, TLE51, TLE54, TLE55, TLE57, TLE60, TLE61, TLE63, TLE64, TLE65, TLE66, TLE67, TLE68.
⧫ **Acute Myeloid Leukemia** (190 samples):
• Source T1 = TCGA (see Acknowledgments (main text)). Sample IDs are of the form TCGA-AB-*, where * is:
  2802, 2803, 2804, 2805, 2806, 2807, 2808, 2809, 2810, 2811, 2812, 2813, 2814, 2816, 2817, 2818, 2819, 2820, 2821, 2822, 2824, 2825, 2826, 2827, 2828, 2829, 2830, 2831, 2832, 2833, 2835, 2836, 2837, 2838, 2839, 2841, 2842, 2843, 2844, 2845, 2846, 2847, 2849, 2850, 2851, 2853, 2854, 2855, 2857, 2858, 2859, 2860, 2861, 2862, 2863, 2864, 2865, 2866, 2867, 2868, 2869, 2870, 2871, 2872, 2873, 2874, 2875, 2876, 2877, 2878, 2879, 2880, 2881, 2882, 2883, 2884, 2885, 2886, 2887, 2888, 2889, 2890, 2891, 2892, 2893, 2894, 2895, 2896, 2897, 2898, 2899, 2900, 2901, 2904, 2905, 2906, 2907, 2908, 2910, 2911, 2912, 2913, 2914, 2915, 2916, 2917, 2918, 2919, 2920, 2921, 2922, 2923, 2924, 2925, 2926, 2927, 2928, 2929, 2930, 2931, 2932, 2933, 2934, 2935, 2936, 2937, 2938, 2939, 2940, 2941, 2943, 2945, 2946, 2947, 2948, 2949, 2950, 2952, 2954, 2955, 2956, 2957, 2959, 2963, 2964, 2965, 2966, 2967, 2968, 2969, 2970, 2971, 2972, 2973, 2974, 2975, 2976, 2977, 2978, 2979, 2980, 2981, 2982, 2983, 2984, 2985, 2986, 2987, 2988, 2989, 2990, 2991, 2992, 2993, 2994, 2995, 2996, 2997, 2998, 2999, 3000, 3001, 3002, 3005, 3006, 3007, 3008, 3009, 3011, 3012.
⧫ **Adrenocortical Carcinoma** (91 samples):
• Source T2 = TCGA (see Acknowledgments (main text)). Sample IDs are of the form TCGA-*, where * is:
  OR-A5J1, OR-A5J2, OR-A5J3, OR-A5J4, OR-A5J5, OR-A5J6, OR-A5J7, OR-A5J8, OR-A5J9, OR-A5JA, OR-A5JB, OR-A5JC, OR-A5JD, OR-A5JE, OR-A5JF, OR-A5JG, OR-A5JH, OR-A5JI, OR-A5JJ, OR-A5JK, OR-A5JL, OR-A5JM, OR-A5JO, OR-A5JP, OR-A5JQ, OR-A5JR, OR-A5JS, OR-A5JT, OR-A5JU, OR-A5JV, OR-A5JW, OR-A5JX, OR-A5JY, OR-A5JZ, OR-A5K0, OR-A5K1, OR-A5K2, OR-A5K3, OR-A5K4, OR-A5K5, OR-A5K6, OR-A5K8, OR-A5K9, OR-A5KB, OR-A5KO, OR-A5KP, OR-A5KQ, OR-A5KS, OR-A5KT, OR-A5KU, OR-A5KV, OR-A5KW, OR-A5KX, OR-A5KY, OR-A5KZ, OR-A5L1, OR-A5L2, OR-A5L3, OR-A5L4, OR-A5L5, OR-A5L6, OR-A5L8, OR-A5L9, OR-A5LA, OR-A5LB, OR-A5LC, OR-A5LD, OR-A5LE, OR-A5LF, OR-A5LG, OR-A5LH, OR-A5LI, OR-A5LJ, OR-A5LK, OR-A5LL, OR-A5LN, OR-A5LO, OR-A5LP, OR-A5LR, OR-A5LS, OR-A5LT, OU-A5PI, P6-A5OF, P6-A5OG, P6-A5OH, PA-A5YG, PK-A5H8, PK-A5H9, PK-A5HA, PK-A5HB, PK-A5HC.
⧫ **B-Cell Lymphoma** (24 samples):
• Source M1 = [49]. In DLBCL sample IDs * runs from A though M (e.g., DLBCL-PatientC):
  07-35482, DLBCL-Patient*, FL-PatientA, FL009.
• Source L1 = [50]:
  1060, 1061, 1065, 1093, 1096, 1102, 515, EB2.
⧫ **Benign Liver Tumor** (40 samples):
• Source P1 = [51]. Sample IDs are of the form CHC*, where * is:
  1023T, 1124T, 1315T, 1328T, 1329T, 1337T, 1382T, 1383T, 1424T, 1425T, 1428T, 1432T, 1434T, 1439T, 1488T, 1489T, 1665T, 1666T, 1854T, 1916T, 340T, 361TB, 462T, 463T, 464T, 470T, 471T, 517T, 575T, 578T, 603T, 605T, 623T, 624T, 674T, 687T, 689T, 846T, 918T, 976T.
⧫ **Bladder Cancer** (341 samples):
• Source G1 = [52]. Sample IDs are of the form TCC+AF8-B**+AC0-Tumor, where ** is (below * stands for +AC0-, e.g., 104*0 = 104+AC0-0, and the full sample ID is TCC+AF8-B104+AC0-0+AC0-Tumor):
  10, 100, 101, 102, 103, 104*0, 104, 105*0, 105*1, 105, 106, 107, 109, 11, 110, 111, 112, 114, 13, 14, 15, 16, 17, 18, 19, 2, 20, 21, 22, 23, 24, 25, 34, 35, 37, 41, 43, 45, 47, 5, 50, 52, 54, 55, 56, 57, 58, 59*0, 59*1, 59*3, 59, 60, 61, 62*0, 63, 64, 65, 66*0, 66, 68, 70, 71, 73, 74, 77, 78, 79, 8, 80*0, 80*1, 80*11, 80*13, 80*3, 80*4, 80*5, 80*7, 80*8, 80, 81*1, 81*2, 81, 82, 83, 84, 85*0, 85*2, 86, 87, 88, 89*1, 89*10, 89*11, 89*12, 89*16, 89*3, 89*4, 89*5, 9, 90, 92, 96, 98, 99.
• Source T3 = TCGA (see Acknowledgments (main text)). Sample IDs are of the form TCGA+AC0-**, where ** is (below * stands for +AC0-A, e.g., BL*0C8 = BL+AC0-A0C8, and the full sample ID is TCGA+AC0-BL+AC0-A0C8; also, below ⋆ = 3OO = 3-double-O):
  BL*0C8, BL*13I, BL*13J, BL*3JM, BL*5ZZ, BT*0S7, BT*0YX, BT*20J, BT*20N, BT*20O, BT*20P, BT*20Q, BT*20R, BT*20T, BT*20U, BT*20V, BT*20W, BT*20X, BT*2LA, BT*2LB, BT*2LD, BT*3PH, BT*3PJ, BT*3PK, BT*42B, BT*42C, BT*42E, BT*42F, C4*0EZ, C4*0F0, C4*0F1, C4*0F6, C4*0F7, CF*1HR, CF*1HS, CF*27C, CF*3MF, CF*3MG, CF*3MH, CF*3MI, CF*47S, CF*47T, CF*47V, CF*47W, CF*47X, CF*47Y, CF*5U8, CF*5UA, CU*0YN, CU*0YO, CU*0YR, CU*3KJ, CU*3QU, CU*3YL, CU*5W6, CU*72E, DK*1A3, DK*1A5, DK*1A6, DK*1A7, DK*1AA, DK*1AB, DK*1AC, DK*1AD, DK*1AE, DK*1AF, DK*1AG, DK*2HX, DK*2I1, DK*2I2, DK*2I4, DK*2I6, DK*3IK, DK*3IL, DK*3IM, DK*3IN, DK*3IQ, DK*3IS, DK*3IT, DK*3IU, DK*3IV, DK*3WW, DK*3WX, DK*3WY, DK*3X1, DK*3X2, DK*6AV, DK*6AW, DK*6B0, DK*6B1, DK*6B2, DK*6B5, DK*6B6, E5*2PC, E5*4TZ, E5*4U1, E7*3X6, E7*3Y1, E7*4IJ, E7*4XJ, E7*541, E7*5KE, E7*5KF, E7*677, E7*678, E7*6ME, E7*6MF, E7*7DU, E7*7DV, FD*3B3, FD*3B4, FD*3B5, FD*3B6, FD*3B7, FD*3B8, FD*3N5, FD*3N6, FD*3NA, FD*3SJ, FD*3SL, FD*3SM, FD*3SN, FD*3SO, FD*3SP, FD*3SQ, FD*3SR, FD*3SS, FD*43N, FD*43P, FD*43S, FD*43U, FD*43X, FD*5BR, FD*5BS, FD*5BU, FD*5BV, FD*5BX, FD*5BY, FD*5BZ, FD*5C0, FD*5C1, FD*62N, FD*62O, FD*62P, FD*62S, FD*6TA, FD*6TB, FD*6TC, FD*6TD, FD*6TE, FD*6TF, FD*6TG, FD*6TH, FD*6TI, FD*6TK, FJ*3Z7, FJ*3Z9, FJ*3ZE, FJ*3ZF, FT*3EE, FT*61P, G2*2EC, G2*2EF, G2*2EJ, G2*2EK, G2*2EL, G2*2EO, G2*2ES, G2*3IB, G2*3IE, G2*3VY, GC*3BM, GC*3I6, GC*⋆, GC*3RB, GC*3RC, GC*3RD, GC*3WC, GC*3YS, GC*6I1, GC*6I3, GD*2C5, GD*3OP, GD*3OQ, GD*3OS, GD*6C6, GD*76B, GU*42P, GU*42Q, GU*42R, GU*762, GU*763, GU*766, GU*767, GV*3JV, GV*3JW, GV*3JX, GV*3JZ, GV*3QF, GV*3QG, GV*3QH, GV*3QI, GV*3QK, GV*40E, GV*40G, GV*6ZA, H4*2HO, H4*2HQ, HQ*2OE, HQ*2OF, HQ*5ND, HQ*5NE, K4*3WS, K4*3WU, K4*3WV, K4*4AB, K4*4AC, K4*54R, K4*5RH, K4*5RI, K4*5RJ, K4*6FZ, K4*6MB, KQ*41N, KQ*41P, KQ*41Q, KQ*41S, LC*66R, LT*5Z6, MV*51V, PQ*6FI, PQ*6FN, R3*69X, S5*6DX, UY*78K, UY*78L, UY*78N, UY*78O.
⧫ **Brain Lower Grade Glioma** (465 samples):
• Source T4 = TCGA (see Acknowledgments (main text)). Sample IDs are of the form TCGA-*, where * is:
  CS-4938, CS-4941, CS-4942, CS-4943, CS-4944, CS-5390, CS-5393, CS-5394, CS-5395, CS-5396, CS-5397, CS-6186, CS-6188, CS-6290, CS-6665, CS-6666, CS-6667, CS-6668, CS-6669, CS-6670, DB-5270, DB-5273, DB-5274, DB-5275, DB-5276, DB-5277, DB-5278, DB-5279, DB-5280, DB-5281, DB-A4X9, DB-A4XA, DB-A4XB, DB-A4XC, DB-A4XD, DB-A4XE, DB-A4XF, DB-A4XG, DB-A4XH, DB-A64L, DB-A64O, DB-A64P, DB-A64Q, DB-A64R, DB-A64S, DB-A64U, DB-A64V, DB-A64W, DB-A64X, DB-A75K, DB-A75L, DB-A75M, DB-A75O, DB-A75P, DH-5140, DH-5141, DH-5142, DH-5143, DH-5144, DH-A669, DH-A66B, DH-A66D, DH-A66F, DH-A66G, DH-A7UR, DH-A7US, DH-A7UT, DH-A7UU, DH-A7UV, DU-5847, DU-5849, DU-5851, DU-5852, DU-5853, DU-5854, DU-5855, DU-5870, DU-5871, DU-5872, DU-5874, DU-6392, DU-6393, DU-6394, DU-6395, DU-6396, DU-6397, DU-6399, DU-6400, DU-6401, DU-6402, DU-6403, DU-6404, DU-6405, DU-6406, DU-6407, DU-6408, DU-6410, DU-6542, DU-7006, DU-7007, DU-7008, DU-7009, DU-7010, DU-7011, DU-7012, DU-7013, DU-7014, DU-7015, DU-7018, DU-7019, DU-7290, DU-7292, DU-7294, DU-7298, DU-7299, DU-7300, DU-7301, DU-7302, DU-7304, DU-7306, DU-7309, DU-8158, DU-8161, DU-8162, DU-8163, DU-8164, DU-8165, DU-8166, DU-8167, DU-8168, DU-A5TP, DU-A5TR, DU-A5TS, DU-A5TT, DU-A5TU, DU-A5TW, DU-A5TY, DU-A6S2, DU-A6S3, DU-A6S6, DU-A6S7, DU-A6S8, DU-A76K, DU-A76L, DU-A76O, DU-A76R, DU-A7T6, DU-A7T8, DU-A7TA, DU-A7TB, DU-A7TC, DU-A7TD, DU-A7TG, DU-A7TJ, E1-5302, E1-5303, E1-5304, E1-5305, E1-5307, E1-5311, E1-5318, E1-5319, E1-5322, E1-A7YD, E1-A7YE, E1-A7YH, E1-A7YI, E1-A7YJ, E1-A7YK, E1-A7YL, E1-A7YM, E1-A7YN, E1-A7YO, E1-A7YQ, E1-A7YS, E1-A7YU, E1-A7YV, E1-A7YW, E1-A7YY, E1-A7Z2, E1-A7Z3, E1-A7Z4, E1-A7Z6, EZ-7264, FG-5962, FG-5963, FG-5964, FG-5965, FG-6688, FG-6689, FG-6690, FG-6691, FG-6692, FG-7634, FG-7636, FG-7637, FG-7638, FG-7641, FG-7643, FG-8181, FG-8182, FG-8185, FG-8186, FG-8187, FG-8188, FG-8189, FG-8191, FG-A4MT, FG-A4MU, FG-A4MW, FG-A4MX, FG-A4MY, FG-A60J, FG-A60K, FG-A60L, FG-A6IZ, FG-A6J1, FG-A6J3, FG-A70Y, FG-A70Z, FG-A710, FG-A711, FG-A713, FN-7833, HT-7467, HT-7468, HT-7469, HT-7470, HT-7471, HT-7472, HT-7473, HT-7474, HT-7475, HT-7476, HT-7477, HT-7478, HT-7479, HT-7480, HT-7481, HT-7482, HT-7483, HT-7485, HT-7601, HT-7602, HT-7603, HT-7604, HT-7605, HT-7606, HT-7607, HT-7608, HT-7609, HT-7610, HT-7611, HT-7616, HT-7620, HT-7676, HT-7677, HT-7680, HT-7681, HT-7684, HT-7686, HT-7687, HT-7688, HT-7689, HT-7690, HT-7691, HT-7692, HT-7693, HT-7694, HT-7695, HT-7854, HT-7855, HT-7856, HT-7857, HT-7858, HT-7860, HT-7873, HT-7874, HT-7875, HT-7877, HT-7879, HT-7880, HT-7881, HT-7882, HT-7884, HT-7902, HT-8010, HT-8011, HT-8012, HT-8013, HT-8015, HT-8018, HT-8019, HT-8104, HT-8105, HT-8106, HT-8107, HT-8108, HT-8109, HT-8110, HT-8111, HT-8113, HT-8114, HT-8558, HT-8563, HT-8564, HT-A4DS, HT-A4DV, HT-A5R5, HT-A5R7, HT-A5R9, HT-A5RA, HT-A5RB, HT-A5RC, HT-A614, HT-A615, HT-A616, HT-A617, HT-A618, HT-A619, HT-A61A, HT-A61B, HT-A61C, HT-A74H, HT-A74J, HT-A74K, HT-A74L, HT-A74O, HW-7486, HW-7487, HW-7489, HW-7490, HW-7491, HW-7493, HW-7495, HW-8319, HW-8320, HW-8321, HW-8322, HW-A5KJ, HW-A5KK, HW-A5KL, HW-A5KM, IK-7675, IK-8125, KT-A74X, KT-A7W1, P5-A5ET, P5-A5EU, P5-A5EV, P5-A5EW, P5-A5EX, P5-A5EY, P5-A5EZ, P5-A5F0, P5-A5F1, P5-A5F2, P5-A5F4, P5-A5F6, P5-A72U, P5-A72W, P5-A72X, P5-A72Z, P5-A730, P5-A731, P5-A733, P5-A735, P5-A736, P5-A737, P5-A77W, P5-A77X, P5-A780, P5-A781, QH-A65R, QH-A65S, QH-A65V, QH-A65X, QH-A65Z, QH-A6CS, QH-A6CU, QH-A6CV, QH-A6CW, QH-A6CX, QH-A6CY, QH-A6CZ, QH-A6X3, QH-A6X4, QH-A6X5, QH-A6X8, QH-A6X9, QH-A6XA, QH-A6XC, R8-A6MK, R8-A6ML, R8-A6MO, R8-A6YH, R8-A73M, S9-A6TS, S9-A6TU, S9-A6TV, S9-A6TW, S9-A6TX, S9-A6TY, S9-A6TZ, S9-A6U0, S9-A6U1, S9-A6U2, S9-A6U5, S9-A6U6, S9-A6U8, S9-A6U9, S9-A6UA, S9-A6UB, S9-A6WD, S9-A6WE, S9-A6WG, S9-A6WH, S9-A6WI, S9-A6WL, S9-A6WM, S9-A6WN, S9-A6WO, S9-A6WP, S9-A6WQ, S9-A7IQ, S9-A7IS, S9-A7IX, S9-A7IY, S9-A7IZ, S9-A7J0, S9-A7J1, S9-A7J2, S9-A7J3, S9-A7QW, S9-A7QX, S9-A7QY, S9-A7QZ, S9-A7R1, S9-A7R2, S9-A7R3, S9-A7R4, S9-A7R7, S9-A7R8, TM-A7C3, TM-A7C4, TM-A7C5, TM-A7CA, TM-A7CF, TQ-A7RF, TQ-A7RG, TQ-A7RH, TQ-A7RI, TQ-A7RJ, TQ-A7RK, TQ-A7RM, TQ-A7RN, TQ-A7RO, TQ-A7RP, TQ-A7RQ, TQ-A7RR, TQ-A7RS, TQ-A7RU, TQ-A7RV, TQ-A7RW, VW-A7QS.
⧫ **Breast Cancer** (1182 samples):
• Source N1 = [53]. Sample IDs are of the form CGP_specimen_*, where * is:
  1096043, 1142475, 1142532, 1142534, 1192095, 1192097, 1192099, 1192101, 1192103, 1192105, 1192107, 1192111, 1192113, 1192115, 1192117, 1192119, 1192121, 1192123, 1192125, 1192127, 1192129, 1192131, 1192133, 1192135, 1192137, 1195364, 1195366, 1195368, 1212804, 1212810, 1212816, 1212822, 1212825, 1212828, 1215490, 1215532, 1215535, 1215553, 1215559, 1215561, 1215563, 1215565, 1215567, 1215573, 1223855, 1223858, 1223861, 1227889, 1227916, 1227918, 1227920, 1227922, 1227924, 1227926, 1227928, 1227951, 1227953, 1227955, 1227957, 1227959, 1227961, 1227963, 1227965, 1227969, 1227971, 1241537, 1241539, 1241541, 1241543, 1241545, 1241547, 1241549, 1241551, 1241553, 1241555, 1241557, 1241559, 1241562, 1241565, 1241568, 1241571, 1241574, 1241579, 1241581, 1261287, 1261291, 1261293, 1261295, 1261297, 1261299, 1261301, 1261303, 1261305, 1261307, 1261309, 1261311, 1261313, 1261337, 1261382, 1261391, 1266549, 1266551, 1266553, 1266561, 1266563, 1266565, 1266567, 1343241, 1343244, 1343247, 1380057, 1380059, 1380061, 1380063, 1380065, 1380067.
• Source S1 = [54]. Sample IDs are of the form PD*a, where * is:
  4842, 4843, 4844, 4934, 4935, 4936, 4937, 4938, 4939, 5961, 7206, 7211, 7316, 9193.
• Source S2 = [55]. Sample IDs are of the form SA*, where * is:
  018, 029, 030, 031, 051, 052, 053, 054, 055, 063, 065, 067, 068, 069, 071, 072, 073, 074, 075, 076, 077, 080, 083, 084, 085, 089, 090, 092, 093, 094, 096, 097, 098, 101, 102, 103, 106, 208, 210, 212, 213, 214, 215, 216, 217, 218, 219, 220, 222, 223, 224, 225, 226, 227, 228, 229, 230, 231, 233, 234, 235, 236, 237.
• Source T5 = TCGA (see Acknowledgments (main text)). Sample IDs are of the form TCGA-*, where * is:
  A1-A0SB, A1-A0SD, A1-A0SE, A1-A0SF, A1-A0SG, A1-A0SH, A1-A0SI, A1-A0SJ, A1-A0SK, A1-A0SM, A1-A0SN, A1-A0SO, A1-A0SP, A1-A0SQ, A2-A04N, A2-A04P, A2-A04Q, A2-A04R, A2-A04T, A2-A04U, A2-A04V, A2-A04W, A2-A04X, A2-A04Y, A2-A0CK, A2-A0CL, A2-A0CM, A2-A0CO, A2-A0CP, A2-A0CQ, A2-A0CR, A2-A0CS, A2-A0CT, A2-A0CU, A2-A0CV, A2-A0CW, A2-A0CX, A2-A0CZ, A2-A0D0, A2-A0D1, A2-A0D2, A2-A0D3, A2-A0D4, A2-A0EM, A2-A0EN, A2-A0EO, A2-A0EP, A2-A0EQ, A2-A0ER, A2-A0ES, A2-A0ET, A2-A0EU, A2-A0EV, A2-A0EW, A2-A0EX, A2-A0EY, A2-A0ST, A2-A0SU, A2-A0SV, A2-A0SW, A2-A0SX, A2-A0SY, A2-A0T0, A2-A0T1, A2-A0T2, A2-A0T3, A2-A0T4, A2-A0T5, A2-A0T6, A2-A0T7, A2-A0YC, A2-A0YD, A2-A0YE, A2-A0YF, A2-A0YG, A2-A0YH, A2-A0YI, A2-A0YJ, A2-A0YK, A2-A0YL, A2-A0YM, A2-A0YT, A2-A1FV, A2-A1FW, A2-A1FX, A2-A1FZ, A2-A1G0, A2-A1G1, A2-A1G4, A2-A1G6, A2-A259, A2-A25A, A2-A25B, A2-A25C, A2-A25D, A2-A25E, A2-A25F, A2-A3KC, A2-A3KD, A2-A3XS, A2-A3XT, A2-A3XU, A2-A3XV, A2-A3XW, A2-A3XX, A2-A3XY, A2-A3XZ, A2-A3Y0, A2-A4RW, A2-A4RX, A2-A4RY, A2-A4S0, A2-A4S1, A2-A4S2, A2-A4S3, A7-A0CD, A7-A0CE, A7-A0CG, A7-A0CH, A7-A0CJ, A7-A0D9, A7-A0DA, A7-A0DB, A7-A0DC, A7-A13D, A7-A13E, A7-A13F, A7-A13G, A7-A13H, A7-A26E, A7-A26F, A7-A26G, A7-A26H, A7-A26I, A7-A26J, A7-A2KD, A7-A3IY, A7-A3IZ, A7-A3J0, A7-A3J1, A7-A3RF, A7-A425, A7-A426, A7-A4SA, A7-A4SB, A7-A4SC, A7-A4SD, A7-A4SE, A7-A4SF, A7-A56D, A7-A5ZV, A7-A5ZW, A7-A5ZX, A8-A06N, A8-A06O, A8-A06P, A8-A06Q, A8-A06R, A8-A06T, A8-A06U, A8-A06X, A8-A06Y, A8-A06Z, A8-A075, A8-A076, A8-A079, A8-A07B, A8-A07C, A8-A07E, A8-A07F, A8-A07G, A8-A07I, A8-A07J, A8-A07L, A8-A07O, A8-A07P, A8-A07R, A8-A07U, A8-A07W, A8-A07Z, A8-A081, A8-A082, A8-A083, A8-A084, A8-A085, A8-A086, A8-A08A, A8-A08B, A8-A08F, A8-A08G, A8-A08H, A8-A08I, A8-A08J, A8-A08L, A8-A08O, A8-A08P, A8-A08R, A8-A08S, A8-A08T, A8-A08X, A8-A08Z, A8-A090, A8-A091, A8-A092, A8-A093, A8-A094, A8-A095, A8-A096, A8-A097, A8-A099, A8-A09A, A8-A09B, A8-A09C, A8-A09D, A8-A09E, A8-A09G, A8-A09I, A8-A09K, A8-A09M, A8-A09N, A8-A09Q, A8-A09R, A8-A09T, A8-A09V, A8-A09W, A8-A09X, A8-A09Z, A8-A0A1, A8-A0A2, A8-A0A4, A8-A0A6, A8-A0A7, A8-A0A9, A8-A0AB, A8-A0AD, AC-A23C, AC-A23E, AC-A23G, AC-A23H, AC-A2B8, AC-A2BK, AC-A2BM, AC-A2FB, AC-A2FE, AC-A2FF, AC-A2FG, AC-A2FK, AC-A2FM, AC-A2FO, AC-A2QH, AC-A2QI, AC-A2QJ, AC-A3BB, AC-A3EH, AC-A3HN, AC-A3OD, AC-A3QP, AC-A3TM, AC-A3TN, AC-A3W5, AC-A3W6, AC-A3W7, AC-A3YI, AC-A3YJ, AC-A5EH, AC-A5EI, AC-A5XS, AC-A5XU, AC-A62X, AC-A62Y, AN-A03X, AN-A03Y, AN-A041, AN-A046, AN-A049, AN-A04A, AN-A04C, AN-A04D, AN-A0AJ, AN-A0AK, AN-A0AL, AN-A0AM, AN-A0AR, AN-A0AS, AN-A0AT, AN-A0FD, AN-A0FF, AN-A0FJ, AN-A0FK, AN-A0FL, AN-A0FN, AN-A0FS, AN-A0FT, AN-A0FV, AN-A0FW, AN-A0FX, AN-A0FY, AN-A0FZ, AN-A0G0, AN-A0XL, AN-A0XN, AN-A0XO, AN-A0XP, AN-A0XR, AN-A0XS, AN-A0XT, AN-A0XU, AN-A0XV, AN-A0XW, AO-A03L, AO-A03M, AO-A03N, AO-A03O, AO-A03P, AO-A03R, AO-A03T, AO-A03U, AO-A03V, AO-A0J2, AO-A0J3, AO-A0J4, AO-A0J5, AO-A0J6, AO-A0J7, AO-A0J8, AO-A0J9, AO-A0JA, AO-A0JB, AO-A0JC, AO-A0JD, AO-A0JE, AO-A0JF, AO-A0JG, AO-A0JI, AO-A0JJ, AO-A0JL, AO-A0JM, AO-A124, AO-A125, AO-A126, AO-A128, AO-A129, AO-A12A, AO-A12B, AO-A12D, AO-A12E, AO-A12F, AO-A12G, AO-A12H, AO-A1KO, AO-A1KP, AO-A1KR, AO-A1KS, AO-A1KT, AQ-A04H, AQ-A04J, AQ-A04L, AQ-A0Y5, AQ-A1H2, AQ-A1H3, AQ-A54N, AQ-A54O, AR-A0TP, AR-A0TQ, AR-A0TR, AR-A0TS, AR-A0TT, AR-A0TU, AR-A0TV, AR-A0TW, AR-A0TX, AR-A0TY, AR-A0TZ, AR-A0U0, AR-A0U1, AR-A0U2, AR-A0U3, AR-A0U4, AR-A1AH, AR-A1AI, AR-A1AJ, AR-A1AK, AR-A1AL, AR-A1AM, AR-A1AN, AR-A1AO, AR-A1AP, AR-A1AQ, AR-A1AR, AR-A1AS, AR-A1AT, AR-A1AU, AR-A1AV, AR-A1AW, AR-A1AX, AR-A1AY, AR-A24H, AR-A24K, AR-A24L, AR-A24M, AR-A24N, AR-A24O, AR-A24P, AR-A24Q, AR-A24R, AR-A24S, AR-A24T, AR-A24U, AR-A24V, AR-A24W, AR-A24X, AR-A24Z, AR-A250, AR-A251, AR-A252, AR-A254, AR-A255, AR-A256, AR-A2LE, AR-A2LH, AR-A2LJ, AR-A2LK, AR-A2LL, AR-A2LM, AR-A2LN, AR-A2LO, AR-A2LQ, AR-A2LR, AR-A5QM, AR-A5QN, AR-A5QP, AR-A5QQ, B6-A0I1, B6-A0I2, B6-A0I5, B6-A0I6, B6-A0I8, B6-A0I9, B6-A0IA, B6-A0IB, B6-A0IC, B6-A0IE, B6-A0IG, B6-A0IH, B6-A0IJ, B6-A0IK, B6-A0IM, B6-A0IN, B6-A0IO, B6-A0IP, B6-A0IQ, B6-A0RE, B6-A0RG, B6-A0RH, B6-A0RI, B6-A0RL, B6-A0RM, B6-A0RN, B6-A0RO, B6-A0RP, B6-A0RQ, B6-A0RS, B6-A0RT, B6-A0RU, B6-A0RV, B6-A0WS, B6-A0WT, B6-A0WV, B6-A0WW, B6-A0WX, B6-A0WY, B6-A0WZ, B6-A0X0, B6-A0X1, B6-A0X4, B6-A0X5, B6-A0X7, B6-A1KC, B6-A1KF, B6-A1KI, B6-A1KN, B6-A2IU, B6-A3ZX, B6-A400, B6-A401, B6-A402, B6-A408, B6-A409, B6-A40B, B6-A40C, BH-A0AU, BH-A0AV, BH-A0AW, BH-A0AY, BH-A0AZ, BH-A0B0, BH-A0B1, BH-A0B3, BH-A0B4, BH-A0B5, BH-A0B6, BH-A0B7, BH-A0B8, BH-A0B9, BH-A0BA, BH-A0BC, BH-A0BD, BH-A0BF, BH-A0BG, BH-A0BJ, BH-A0BL, BH-A0BM, BH-A0BO, BH-A0BP, BH-A0BQ, BH-A0BR, BH-A0BS, BH-A0BT, BH-A0BV, BH-A0BW, BH-A0BZ, BH-A0C0, BH-A0C1, BH-A0C3, BH-A0C7, BH-A0DD, BH-A0DE, BH-A0DG, BH-A0DH, BH-A0DI, BH-A0DK, BH-A0DL, BH-A0DO, BH-A0DP, BH-A0DQ, BH-A0DS, BH-A0DT, BH-A0DV, BH-A0DX, BH-A0DZ, BH-A0E0, BH-A0E1, BH-A0E2, BH-A0E6, BH-A0E7, BH-A0E9, BH-A0EA, BH-A0EB, BH-A0EE, BH-A0EI, BH-A0GY, BH-A0GZ, BH-A0H0, BH-A0H3, BH-A0H5, BH-A0H6, BH-A0H7, BH-A0H9, BH-A0HA, BH-A0HB, BH-A0HF, BH-A0HI, BH-A0HK, BH-A0HL, BH-A0HN, BH-A0HO, BH-A0HP, BH-A0HQ, BH-A0HU, BH-A0HW, BH-A0HX, BH-A0HY, BH-A0RX, BH-A0W3, BH-A0W4, BH-A0W5, BH-A0W7, BH-A0WA, BH-A18F, BH-A18G, BH-A18H, BH-A18I, BH-A18J, BH-A18K, BH-A18L, BH-A18M, BH-A18N, BH-A18P, BH-A18Q, BH-A18R, BH-A18S, BH-A18T, BH-A18U, BH-A18V, BH-A1EN, BH-A1EO, BH-A1ES, BH-A1ET, BH-A1EU, BH-A1EV, BH-A1EW, BH-A1EX, BH-A1EY, BH-A1F0, BH-A1F2, BH-A1F5, BH-A1F6, BH-A1F8, BH-A1FC, BH-A1FD, BH-A1FE, BH-A1FG, BH-A1FH, BH-A1FJ, BH-A1FL, BH-A1FM, BH-A1FN, BH-A1FR, BH-A1FU, BH-A201, BH-A202, BH-A203, BH-A204, BH-A208, BH-A209, BH-A28O, BH-A28Q, BH-A2L8, BH-A42T, BH-A42U, BH-A42V, BH-A5IZ, BH-A5J0, C8-A12K, C8-A12L, C8-A12M, C8-A12N, C8-A12O, C8-A12P, C8-A12Q, C8-A12T, C8-A12U, C8-A12V, C8-A12W, C8-A12X, C8-A12Y, C8-A12Z, C8-A130, C8-A131, C8-A132, C8-A133, C8-A134, C8-A135, C8-A137, C8-A138, C8-A1HE, C8-A1HF, C8-A1HG, C8-A1HI, C8-A1HJ, C8-A1HK, C8-A1HL, C8-A1HM, C8-A1HN, C8-A1HO, C8-A26V, C8-A26W, C8-A26X, C8-A26Y, C8-A26Z, C8-A273, C8-A274, C8-A275, C8-A278, C8-A27A, C8-A27B, C8-A3M7, C8-A3M8, D8-A13Y, D8-A13Z, D8-A140, D8-A141, D8-A142, D8-A143, D8-A145, D8-A146, D8-A147, D8-A1J8, D8-A1J9, D8-A1JA, D8-A1JB, D8-A1JC, D8-A1JD, D8-A1JE, D8-A1JF, D8-A1JG, D8-A1JH, D8-A1JI, D8-A1JJ, D8-A1JK, D8-A1JL, D8-A1JM, D8-A1JN, D8-A1JP, D8-A1JS, D8-A1JT, D8-A1JU, D8-A1X5, D8-A1X6, D8-A1X7, D8-A1X8, D8-A1X9, D8-A1XA, D8-A1XB, D8-A1XC, D8-A1XF, D8-A1XG, D8-A1XJ, D8-A1XK, D8-A1XL, D8-A1XM, D8-A1XO, D8-A1XQ, D8-A1XR, D8-A1XS, D8-A1XT, D8-A1XU, D8-A1XV, D8-A1XW, D8-A1XY, D8-A1XZ, D8-A1Y0, D8-A1Y1, D8-A1Y2, D8-A1Y3, D8-A27E, D8-A27F, D8-A27G, D8-A27H, D8-A27I, D8-A27K, D8-A27L, D8-A27M, D8-A27N, D8-A27P, D8-A27R, D8-A27T, D8-A27V, D8-A27W, D8-A3Z5, D8-A3Z6, D8-A4Z1, E2-A105, E2-A107, E2-A108, E2-A109, E2-A10A, E2-A10B, E2-A10C, E2-A10E, E2-A10F, E2-A14N, E2-A14O, E2-A14P, E2-A14Q, E2-A14R, E2-A14S, E2-A14T, E2-A14U, E2-A14V, E2-A14W, E2-A14X, E2-A14Y, E2-A14Z, E2-A150, E2-A152, E2-A153, E2-A154, E2-A155, E2-A156, E2-A158, E2-A159, E2-A15A, E2-A15C, E2-A15D, E2-A15E, E2-A15F, E2-A15G, E2-A15H, E2-A15I, E2-A15J, E2-A15K, E2-A15L, E2-A15M, E2-A15O, E2-A15P, E2-A15R, E2-A15S, E2-A15T, E2-A1AZ, E2-A1B0, E2-A1B1, E2-A1B4, E2-A1B5, E2-A1B6, E2-A1BC, E2-A1BD, E2-A1IE, E2-A1IF, E2-A1IG, E2-A1IH, E2-A1II, E2-A1IJ, E2-A1IK, E2-A1IL, E2-A1IN, E2-A1IO, E2-A1IU, E2-A1L6, E2-A1L7, E2-A1L8, E2-A1L9, E2-A1LA, E2-A1LB, E2-A1LE, E2-A1LG, E2-A1LH, E2-A1LI, E2-A1LK, E2-A1LL, E2-A1LS, E2-A2P5, E2-A2P6, E2-A3DX, E2-A56Z, E2-A570, E2-A573, E2-A574, E9-A1N3, E9-A1N4, E9-A1N5, E9-A1N8, E9-A1N9, E9-A1NA, E9-A1NC, E9-A1ND, E9-A1NE, E9-A1NF, E9-A1NG, E9-A1NH, E9-A1NI, E9-A1QZ, E9-A1R0, E9-A1R2, E9-A1R3, E9-A1R4, E9-A1R5, E9-A1R6, E9-A1R7, E9-A1RA, E9-A1RB, E9-A1RC, E9-A1RD, E9-A1RE, E9-A1RF, E9-A1RG, E9-A1RH, E9-A1RI, E9-A226, E9-A227, E9-A228, E9-A229, E9-A22A, E9-A22B, E9-A22D, E9-A22E, E9-A22G, E9-A22H, E9-A243, E9-A244, E9-A245, E9-A247, E9-A248, E9-A249, E9-A24A, E9-A295, E9-A2JS, E9-A2JT, E9-A3HO, E9-A3Q9, E9-A3QA, E9-A3X8, E9-A54X, E9-A54Y, E9-A5FK, E9-A5FL, E9-A5UO, E9-A5UP, EW-A1IW, EW-A1IX, EW-A1IY, EW-A1IZ, EW-A1J1, EW-A1J2, EW-A1J3, EW-A1J5, EW-A1J6, EW-A1OV, EW-A1OX, EW-A1OY, EW-A1OZ, EW-A1P0, EW-A1P1, EW-A1P3, EW-A1P4, EW-A1P5, EW-A1P6, EW-A1P7, EW-A1P8, EW-A1PA, EW-A1PB, EW-A1PC, EW-A1PD, EW-A1PE, EW-A1PG, EW-A1PH, EW-A2FR, EW-A2FS, EW-A2FV, EW-A2FW, EW-A3E8, EW-A3U0, EW-A423, GI-A2C8, GI-A2C9, GM-A2D9, GM-A2DA, GM-A2DB, GM-A2DC, GM-A2DD, GM-A2DF, GM-A2DH, GM-A2DI, GM-A2DK, GM-A2DL, GM-A2DM, GM-A2DN, GM-A2DO, GM-A3NW, GM-A3NY, GM-A3XG, GM-A3XL, GM-A3XN, GM-A4E0, GM-A5PV, GM-A5PX, HN-A2NL, HN-A2OB, JL-A3YW, JL-A3YX, LL-A440, LL-A441, LL-A50Y, LL-A5YL, LL-A5YM, LL-A5YN, LL-A5YO, LL-A5YP, LQ-A4E4, MS-A51U, OK-A5Q2, OL-A5D6, OL-A5D7, OL-A5D8, OL-A5DA, OL-A5RU, OL-A5RV, OL-A5RW, OL-A5RX, OL-A5RY, OL-A5RZ, OL-A5S0, OL-A66H, OL-A66I, OL-A66J, OL-A66K, PE-A5DC, PE-A5DD, PE-A5DE.
⧫ **Cervical Cancer** (197 samples):
• Source T6 = TCGA (see Acknowledgments (main text)). Sample IDs are of the form TCGA-*, where * is:
  BI-A0VR, BI-A0VS, BI-A20A, C5-A0TN, C5-A1BE, C5-A1BF, C5-A1BI, C5-A1BJ, C5-A1BK, C5-A1BL, C5-A1BM, C5-A1BN, C5-A1BQ, C5-A1M5, C5-A1M6, C5-A1M7, C5-A1M8, C5-A1M9, C5-A1ME, C5-A1MF, C5-A1MH, C5-A1MI, C5-A1MJ, C5-A1MK, C5-A1ML, C5-A1MN, C5-A1MP, C5-A1MQ, C5-A2LS, C5-A2LT, C5-A2LV, C5-A2LX, C5-A2LY, C5-A2LZ, C5-A2M1, C5-A2M2, C5-A3HD, C5-A3HE, C5-A3HF, C5-A3HL, C5-A7CG, C5-A7CH, C5-A7CJ, C5-A7CK, C5-A7CL, C5-A7CM, C5-A7CO, C5-A7UC, C5-A7UE, C5-A7UH, C5-A7X3, DG-A2KH, DG-A2KJ, DG-A2KK, DG-A2KL, DG-A2KM, DR-A0ZL, DR-A0ZM, DS-A0VK, DS-A0VL, DS-A0VM, DS-A0VN, DS-A1OA, DS-A3LQ, DS-A5RQ, DS-A7WF, DS-A7WH, DS-A7WI, EA-A1QS, EA-A1QT, EA-A3HQ, EA-A3HR, EA-A3HT, EA-A3HU, EA-A3QD, EA-A3QE, EA-A3Y4, EA-A410, EA-A411, EA-A439, EA-A43B, EA-A44S, EA-A4BA, EA-A50E, EA-A556, EA-A5FO, EA-A5O9, EA-A5ZD, EA-A5ZE, EA-A5ZF, EA-A6QX, EA-A78R, EK-A2GZ, EK-A2H0, EK-A2H1, EK-A2IP, EK-A2PG, EK-A2PI, EK-A2PK, EK-A2PL, EK-A2PM, EK-A2R7, EK-A2R8, EK-A2R9, EK-A2RA, EK-A2RB, EK-A2RC, EK-A2RD, EK-A2RE, EK-A2RJ, EK-A2RK, EK-A2RL, EK-A2RM, EK-A2RN, EK-A2RO, EK-A3GJ, EK-A3GK, EK-A3GM, EK-A3GN, EX-A1H5, EX-A1H6, EX-A3L1, EX-A449, EX-A69L, EX-A69M, FU-A23K, FU-A23L, FU-A2QG, FU-A3EO, FU-A3HY, FU-A3HZ, FU-A3NI, FU-A3TQ, FU-A3TX, FU-A3WB, FU-A3YQ, FU-A40J, FU-A57G, FU-A5XV, FU-A770, HG-A2PA, HM-A3JJ, HM-A3JK, HM-A4S6, HM-A6W2, IR-A3L7, IR-A3LA, IR-A3LB, IR-A3LC, IR-A3LF, IR-A3LH, IR-A3LI, IR-A3LK, IR-A3LL, JW-A5VG, JW-A5VH, JW-A5VI, JW-A5VJ, JW-A5VK, JW-A5VL, JW-A69B, JW-A852, JX-A3PZ, JX-A3Q0, JX-A3Q8, JX-A5QV, LP-A4AU, LP-A4AV, LP-A4AW, LP-A4AX, LP-A5U2, LP-A5U3, LP-A7HU, MU-A51Y, MU-A5YI, MY-A5BD, MY-A5BE, MY-A5BF, Q1-A5R1, Q1-A5R2, Q1-A5R3, Q1-A6DT, Q1-A6DV, Q1-A6DW, Q1-A73O, Q1-A73P, Q1-A73Q, Q1-A73R, Q1-A73S, R2-A69V, RA-A741, UC-A7PD, UC-A7PF, WL-A834, DS-A1OB, DS-A1OC, DS-A1OD.
⧫ **Cholangiocarcinoma** (139 samples):
• Source Z2 = [56]:
  1, 10, 100, 101, 107, 108, 109, 110, 111, 112, 113, 115, 116, 118, 119, 120, 121, 122, 123, 125, 127, 128, 129, 13, 130, 131, 132, 133, 134, 135, 137, 139, 140, 141, 142, 143, 144, 145, 146, 147, 16, 17, 18, 19, 2, 20, 24, 25, 26, 28, 29, 3, 33, 34, 35, 39, 41, 42, 44, 46, 48, 5, 50, 51, 52, 53, 56, 58, 59, 6, 60, 61, 63, 64, 66, 67, 69, 7, 70, 71, 74, 79, 8, 8_1, 8_2, 8_4, 8_6, 80, 81, 82, 85, 86, 87, 88, 89, 9, 90, 91, 94, 95, 97, 98, 99.
• Source T7 = TCGA (see Acknowledgments (main text)). Sample IDs are of the form TCGA-*, where * is:
  3X-AAV9, 3X-AAVA, 3X-AAVB, 3X-AAVC, 3X-AAVE, 4G-AAZO, 4G-AAZT, W5-AA2G, W5-AA2H, W5-AA2I, W5-AA2O, W5-AA2Q, W5-AA2R, W5-AA2T, W5-AA2U, W5-AA2W, W5-AA2X, W5-AA2Z, W5-AA30, W5-AA31, W5-AA33, W5-AA34, W5-AA36, W5-AA38, W5-AA39, W6-AA0S, WD-A7RX, YR-A95A, ZD-A8I3, ZH-A8Y1, ZH-A8Y2, ZH-A8Y4, ZH-A8Y5, ZH-A8Y6, ZH-A8Y8, ZU-A8S4.
⧫ **Chronic Lymphocytic Leukemia** (80 samples):
• Source Q1 = [57]:
  170, 171, 172, 173, 174, 175, 18, 181, 182, 184, 185, 186, 188, 189, 19, 191, 193, 194, 195, 197, 20, 22, 23, 264, 266, 267, 27, 270, 272, 273, 274, 275, 276, 278, 279, 280, 29, 290, 30, 319, 32, 321, 322, 323, 324, 325, 326, 328, 33, 375, 39, 40, 41, 42, 43, 44, 45, 48, 49, 5, 51, 52, 53, 54, 6, 618, 63, 64, 642, 680, 7, 758, 761, 785, 8, 82, 83, 9, 90, 91.
⧫ **Colorectal Cancer** (581 samples):
• Source S3 = [58]:
  587220, 587222, 587224, 587226, 587228, 587230, 587232, 587234, 587238, 587242, 587246, 587254, 587256, 587260, 587262, 587264, 587268, 587270, 587276, 587278, 587282, 587284, 587286, 587288, 587290, 587292, 587294, 587298, 587300, 587302, 587304, 587306, 587316, 587318, 587322, 587328, 587330, 587332, 587334, 587336, 587338, 587340, 587342, 587344, 587346, 587348, 587350, 587352, 587354, 587356, 587358, 587360, 587362, 587364, 587368, 587370, 587372, 587374, 587376, 587378, 587380, 587382, 587384, 587386, 587388, 587390, 587392, 587394, 587398, 587400.
• Source T8 = TCGA (see Acknowledgments (main text)). Sample IDs are of the form TCGA-*, where * is:
  A6-2670, A6-2671, A6-2672, A6-2674, A6-2675, A6-2676, A6-2677, A6-2678, A6-2683, A6-3807, A6-3808, A6-3809, A6-3810, A6-4105, A6-5656, A6-5657, A6-5659, A6-5660, A6-5661, A6-5662, A6-5664, A6-5665, A6-5666, A6-5667, A6-6137, A6-6138, A6-6140, A6-6141, A6-6142, A6-6648, A6-6649, A6-6650, A6-6651, A6-6652, A6-6653, A6-6654, A6-6780, A6-6781, A6-6782, AA-3489, AA-3492, AA-3496, AA-3502, AA-3510, AA-3511, AA-3514, AA-3516, AA-3517, AA-3518, AA-3519, AA-3520, AA-3521, AA-3522, AA-3524, AA-3525, AA-3526, AA-3527, AA-3529, AA-3531, AA-3532, AA-3534, AA-3538, AA-3542, AA-3543, AA-3544, AA-3548, AA-3549, AA-3552, AA-3553, AA-3554, AA-3555, AA-3556, AA-3558, AA-3560, AA-3561, AA-3562, AA-3655, AA-3660, AA-3662, AA-3663, AA-3664, AA-3666, AA-3667, AA-3672, AA-3673, AA-3678, AA-3679, AA-3680, AA-3681, AA-3684, AA-3685, AA-3688, AA-3692, AA-3693, AA-3695, AA-3696, AA-3697, AA-3710, AA-3712, AA-3713, AA-3715, AA-3811, AA-3812, AA-3814, AA-3815, AA-3818, AA-3819, AA-3821, AA-3831, AA-3833, AA-3837, AA-3842, AA-3844, AA-3845, AA-3846, AA-3848, AA-3850, AA-3851, AA-3852, AA-3854, AA-3855, AA-3856, AA-3858, AA-3860, AA-3861, AA-3864, AA-3866, AA-3867, AA-3869, AA-3870, AA-3872, AA-3875, AA-3877, AA-3930, AA-3939, AA-3941, AA-3947, AA-3949, AA-3950, AA-3952, AA-3955, AA-3956, AA-3966, AA-3968, AA-3971, AA-3972, AA-3973, AA-3975, AA-3977, AA-3979, AA-3980, AA-3982, AA-3984, AA-3986, AA-3989, AA-3994, AA-A004, AA-A00A, AA-A00D, AA-A00E, AA-A00F, AA-A00J, AA-A00K, AA-A00L, AA-A00N, AA-A00O, AA-A00Q, AA-A00R, AA-A00U, AA-A00W, AA-A00Z, AA-A010, AA-A017, AA-A01D, AA-A01F, AA-A01G, AA-A01I, AA-A01K, AA-A01P, AA-A01Q, AA-A01R, AA-A01S, AA-A01T, AA-A01V, AA-A01X, AA-A01Z, AA-A022, AA-A024, AA-A029, AA-A02F, AA-A02H, AA-A02J, AA-A02K, AA-A02O, AA-A02W, AA-A02Y, AA-A03F, AA-A03J, AD-5900, AD-6548, AD-6888, AD-6889, AD-6890, AD-6895, AD-6899, AD-6901, AD-6963, AD-6964, AD-6965, AF-2687, AF-2689, AF-2691, AF-2692, AF-2693, AF-3400, AF-3913, AF-4110, AF-5654, AF-6136, AF-6655, AF-6672, AG-3574, AG-3575, AG-3578, AG-3580, AG-3581, AG-3582, AG-3583, AG-3584, AG-3586, AG-3587, AG-3593, AG-3594, AG-3598, AG-3599, AG-3600, AG-3601, AG-3602, AG-3605, AG-3608, AG-3609, AG-3611, AG-3612, AG-3726, AG-3727, AG-3731, AG-3732, AG-3742, AG-3878, AG-3881, AG-3882, AG-3883, AG-3885, AG-3887, AG-3890, AG-3892, AG-3893, AG-3894, AG-3896, AG-3898, AG-3901, AG-3902, AG-3909, AG-3999, AG-4001, AG-4005, AG-4007, AG-4008, AG-4015, AG-A002, AG-A008, AG-A00C, AG-A00H, AG-A00Y, AG-A011, AG-A014, AG-A015, AG-A016, AG-A01L, AG-A01W, AG-A01Y, AG-A020, AG-A025, AG-A026, AG-A02G, AG-A02N, AG-A02X, AG-A032, AG-A036, AH-6544, AH-6547, AH-6549, AH-6643, AH-6644, AM-5820, AM-5821, AU-3779, AU-6004, AY-4070, AY-4071, AY-5543, AY-6196, AY-6197, AY-6386, AZ-4315, AZ-4323, AZ-4615, AZ-4616, AZ-4681, AZ-4682, AZ-5403, AZ-5407, AZ-6598, AZ-6599, AZ-6600, AZ-6601, AZ-6603, AZ-6605, AZ-6606, AZ-6607, AZ-6608, CA-5254, CA-5255, CA-5796, CA-5797, CA-6715, CA-6716, CA-6717, CA-6718, CA-6719, CI-6619, CI-6620, CI-6621, CI-6622, CI-6624, CK-4947, CK-4948, CK-4950, CK-4952, CK-5912, CK-5913, CK-5914, CK-5915, CK-5916, CK-6746, CK-6747, CK-6748, CK-6751, CL-5917, CL-5918, CM-4743, CM-4744, CM-4746, CM-4747, CM-4748, CM-4750, CM-4752, CM-5341, CM-5344, CM-5348, CM-5349, CM-5860, CM-5861, CM-5862, CM-5863, CM-5864, CM-5868, CM-6161, CM-6162, CM-6163, CM-6164, CM-6165, CM-6166, CM-6167, CM-6168, CM-6169, CM-6170, CM-6171, CM-6172, CM-6674, CM-6675, CM-6676, CM-6677, CM-6678, CM-6679, CM-6680, D5-5537, D5-5538, D5-5539, D5-5540, D5-5541, D5-6529, D5-6531, D5-6532, D5-6533, D5-6534, D5-6535, D5-6536, D5-6537, D5-6538, D5-6539, D5-6540, D5-6541, D5-6898, D5-6920, D5-6922, D5-6923, D5-6924, D5-6926, D5-6927, D5-6928, D5-6929, D5-6930, D5-6931, D5-6932, D5-7000, DC-5337, DC-5869, DC-6155, DC-6157, DC-6158, DC-6160, DC-6681, DC-6682, DC-6683, DM-A0X9, DM-A0XD, DM-A0XF, DM-A1D0, DM-A1D4, DM-A1D6, DM-A1D7, DM-A1D8, DM-A1D9, DM-A1DA, DM-A1DB, DM-A1HA, DM-A1HB, DM-A282, DM-A285, DM-A28C, DM-A28E, DM-A28F, DM-A28G, DM-A28H, DM-A28K, DM-A28M, DT-5265, DY-A0XA, DY-A1DC, DY-A1DD, DY-A1DF, DY-A1DG, DY-A1H8, EF-5830, EI-6506, EI-6507, EI-6508, EI-6510, F4-6459, F4-6460, F4-6461, F4-6463, F4-6569, F4-6570, F4-6703, F4-6704, F4-6805, F4-6806, F4-6807, F4-6808, F4-6809, F4-6854, F4-6855, F4-6856, F4-6857, F5-6464, F5-6465, F5-6571, F5-6702, F5-6811, F5-6812, F5-6813, G4-6293, G4-6294, G4-6295, G4-6297, G4-6298, G4-6299, G4-6302, G4-6303, G4-6304, G4-6306, G4-6307, G4-6309, G4-6310, G4-6311, G4-6314, G4-6315, G4-6317, G4-6320, G4-6321, G4-6322, G4-6323, G4-6586, G4-6588, G4-6625, G4-6626, G4-6628, G5-6235, G5-6641.
⧫ **Esophageal Cancer** (329 samples):
• Source D2 = [59]. Sample IDs are of the form ESO-*-Tumor, where * is:
  0001, 0009, 0013, 0015, 0019, 0023, 0025, 0029, 003, 005, 0053, 0059, 0061, 0067, 007, 0071, 0079, 0103, 0115, 0123, 0125, 0129, 0133, 0149, 0167, 017, 0176, 021, 0255, 027, 0280, 037, 043, 045, 0459, 049, 051, 0590, 075, 077, 083, 085, 0950, 105, 1059, 1060, 107, 1096, 111, 1130, 1133, 114, 1145, 1154, 116, 1163, 117, 118, 119, 120, 122, 130, 131, 135, 137, 139, 141, 1427, 143, 147, 1481, 1488, 151, 152, 153, 155, 157, 159, 1594, 160, 1608, 161, 164, 165, 167, 1670, 169, 171, 173, 1733, 1748, 175, 177, 179, 184, 185, 187, 1872, 189, 191, 2143, 224, 2472, 250, 251, 2536, 327, 408, 409, 512, 536, 539, 555, 580, 582, 601, 610, 632, 640, 669, 682, 683, 708, 718, 720, 721, 732, 752, 805, 837, 838, 859, 864, 866, 874, 887, 913, 916, 931, 963, D76, H01, H63, K08, R61, S41.
• Source T9 = TCGA (see Acknowledgments (main text)). Sample IDs are of the form TCGA-*, where * is:
  2H-A9GF, 2H-A9GH, 2H-A9GI, 2H-A9GJ, 2H-A9GK, 2H-A9GL, 2H-A9GM, 2H-A9GN, 2H-A9GO, 2H-A9GQ, 2H-A9GR, IC-A6RE, IC-A6RF, IG-A3I8, IG-A3QL, IG-A3Y9, IG-A3YA, IG-A3YB, IG-A3YC, IG-A4P3, IG-A4QS, IG-A4QT, IG-A50L, IG-A51D, IG-A5B8, IG-A5S3, IG-A625, IG-A6QS, IG-A7DP, IG-A8O2, IG-A97H, IG-A97I, JY-A6F8, JY-A6FA, JY-A6FB, JY-A6FD, JY-A6FE, JY-A6FG, JY-A6FH, JY-A938, JY-A939, JY-A93C, JY-A93D, JY-A93E, JY-A93F, KH-A6WC, L5-A43C, L5-A43E, L5-A43H, L5-A43I, L5-A43J, L5-A43M, L5-A4OE, L5-A4OF, L5-A4OG, L5-A4OH, L5-A4OI, L5-A4OJ, L5-A4OM, L5-A4ON, L5-A4OO, L5-A4OP, L5-A4OQ, L5-A4OR, L5-A4OS, L5-A4OT, L5-A4OU, L5-A4OW, L5-A4OX, L5-A88S, L5-A88T, L5-A88V, L5-A88W, L5-A88Y, L5-A88Z, L5-A891, L5-A893, L5-A8NE, L5-A8NF, L5-A8NG, L5-A8NH, L5-A8NI, L5-A8NJ, L5-A8NK, L5-A8NL, L5-A8NM, L5-A8NN, L5-A8NQ, L5-A8NR, L5-A8NS, L5-A8NT, L5-A8NU, L5-A8NV, L5-A8NW, L7-A56G, L7-A6VZ, LN-A49K, LN-A49L, LN-A49M, LN-A49N, LN-A49O, LN-A49P, LN-A49R, LN-A49S, LN-A49U, LN-A49V, LN-A49W, LN-A49X, LN-A49Y, LN-A4A1, LN-A4A2, LN-A4A3, LN-A4A4, LN-A4A5, LN-A4A6, LN-A4A8, LN-A4A9, LN-A4MQ, LN-A4MR, LN-A5U5, LN-A5U6, LN-A5U7, LN-A7HV, LN-A7HW, LN-A7HX, LN-A7HY, LN-A7HZ, LN-A8HZ, LN-A8I0, LN-A8I1, LN-A9FO, LN-A9FP, LN-A9FQ, LN-A9FR, M9-A5M8, Q9-A6FU, Q9-A6FW, R6-A6DN, R6-A6DQ, R6-A6KZ, R6-A6L4, R6-A6L6, R6-A6XG, R6-A6XQ, R6-A6Y0, R6-A6Y2, R6-A8W5, R6-A8W8, R6-A8WC, R6-A8WG, RE-A7BO, S8-A6BV, S8-A6BW, V5-A7RB, V5-A7RC, V5-A7RE, V5-AASV, V5-AASW, V5-AASX, VR-A8EO, VR-A8EP, VR-A8EQ, VR-A8ER, VR-A8ET, VR-A8EU, VR-A8EW, VR-A8EX, VR-A8EY, VR-A8EZ, VR-A8Q7, VR-AA4D, VR-AA4G, VR-AA7B, VR-AA7D, X8-AAAR, XP-A8T6, XP-A8T7, XP-A8T8, Z6-A8JD, Z6-A8JE, Z6-A9VB, Z6-AAPN, ZR-A9CJ.
⧫ **Gastric Cancer** (401 samples):
• Source Z3 = [60]:
  2000362, 31231321, 76629543, 970010, 98748381, 990089, 990097, 990172, 990300, 990396, 990475, 990515, TGH, TWH.
• Source W1 = [61]. Sample IDs are of the form pfg*T, where * is:
  001, 002, 003, 005, 006, 007, 008, 009, 010, 011, 014, 015, 016, 017, 018, 019, 020, 021, 022, 024, 025, 029.
• Source T10 = TCGA (see Acknowledgments (main text)). Sample IDs are of the form TCGA-*, where * is:
  B7-5816, B7-5818, B7-A5TI, B7-A5TJ, B7-A5TK, B7-A5TN, BR-4183, BR-4184, BR-4186, BR-4187, BR-4188, BR-4190, BR-4191, BR-4194, BR-4195, BR-4197, BR-4199, BR-4200, BR-4201, BR-4205, BR-4253, BR-4255, BR-4256, BR-4257, BR-4259, BR-4261, BR-4263, BR-4264, BR-4265, BR-4267, BR-4271, BR-4273, BR-4276, BR-4277, BR-4278, BR-4279, BR-4280, BR-4281, BR-4283, BR-4284, BR-4286, BR-4288, BR-4291, BR-4292, BR-4294, BR-4298, BR-4357, BR-4361, BR-4362, BR-4363, BR-4366, BR-4368, BR-4369, BR-4370, BR-4371, BR-4375, BR-4376, BR-6452, BR-6453, BR-6454, BR-6455, BR-6456, BR-6457, BR-6458, BR-6563, BR-6564, BR-6565, BR-6566, BR-6705, BR-6706, BR-6707, BR-6709, BR-6801, BR-6802, BR-6803, BR-6852, BR-7196, BR-7197, BR-7703, BR-7704, BR-7707, BR-7715, BR-7716, BR-7717, BR-7722, BR-7723, BR-7851, BR-7901, BR-7957, BR-7958, BR-7959, BR-8058, BR-8059, BR-8060, BR-8077, BR-8078, BR-8080, BR-8081, BR-8284, BR-8285, BR-8286, BR-8289, BR-8291, BR-8295, BR-8296, BR-8297, BR-8360, BR-8361, BR-8363, BR-8364, BR-8365, BR-8366, BR-8367, BR-8368, BR-8369, BR-8370, BR-8371, BR-8372, BR-8373, BR-8380, BR-8381, BR-8382, BR-8384, BR-8483, BR-8484, BR-8485, BR-8486, BR-8487, BR-8588, BR-8589, BR-8590, BR-8591, BR-8592, BR-8676, BR-8677, BR-8678, BR-8679, BR-8680, BR-8682, BR-8683, BR-8686, BR-8687, BR-8690, BR-A44T, BR-A44U, BR-A452, BR-A453, BR-A4CQ, BR-A4CR, BR-A4CS, BR-A4IU, BR-A4IV, BR-A4IY, BR-A4IZ, BR-A4J1, BR-A4J2, BR-A4J4, BR-A4J5, BR-A4J6, BR-A4J7, BR-A4J8, BR-A4PD, BR-A4PE, BR-A4PF, BR-A4QI, BR-A4QL, BR-A4QM, CD-5798, CD-5799, CD-5800, CD-5801, CD-5802, CD-5803, CD-5804, CD-5813, CD-8524, CD-8525, CD-8526, CD-8527, CD-8528, CD-8529, CD-8530, CD-8531, CD-8532, CD-8533, CD-8534, CD-8535, CD-8536, CD-A486, CD-A487, CD-A489, CD-A48A, CD-A48C, CD-A4MG, CD-A4MH, CD-A4MI, CD-A4MJ, CG-4300, CG-4301, CG-4304, CG-4305, CG-4306, CG-4436, CG-4437, CG-4438, CG-4440, CG-4441, CG-4442, CG-4443, CG-4444, CG-4449, CG-4455, CG-4460, CG-4462, CG-4465, CG-4466, CG-4469, CG-4474, CG-4475, CG-4476, CG-4477, CG-5716, CG-5717, CG-5718, CG-5719, CG-5720, CG-5721, CG-5722, CG-5723, CG-5724, CG-5725, CG-5726, CG-5727, CG-5728, CG-5730, CG-5732, CG-5733, CG-5734, D7-5577, D7-5578, D7-5579, D7-6518, D7-6519, D7-6520, D7-6521, D7-6522, D7-6524, D7-6525, D7-6526, D7-6527, D7-6528, D7-6815, D7-6817, D7-6818, D7-6820, D7-6822, D7-8570, D7-8572, D7-8573, D7-8574, D7-8575, D7-8576, D7-8578, D7-8579, D7-A4YT, D7-A4YU, D7-A4YV, D7-A4YX, D7-A4YY, D7-A4Z0, D7-A6ET, D7-A6EV, D7-A6EX, D7-A6EY, D7-A6EZ, D7-A6F0, D7-A6F2, D7-A747, D7-A748, D7-A74A, D7-A74B, EQ-5647, EQ-8122, EQ-A4SO, F1-6177, F1-6874, F1-6875, F1-A448, F1-A72C, FP-7735, FP-7829, FP-7916, FP-7998, FP-8099, FP-8209, FP-8210, FP-8211, FP-8631, FP-A4BE, FP-A4BF, HF-7131, HF-7132, HF-7133, HF-7134, HF-7136, HF-A5NB, HJ-7597, HU-8238, HU-8243, HU-8244, HU-8245, HU-8249, HU-8602, HU-8604, HU-8608, HU-8610, HU-A4G2, HU-A4G3, HU-A4G6, HU-A4G8, HU-A4G9, HU-A4GC, HU-A4GD, HU-A4GF, HU-A4GH, HU-A4GJ, HU-A4GN, HU-A4GP, HU-A4GQ, HU-A4GT, HU-A4GU, HU-A4GX, HU-A4GY, HU-A4H0, HU-A4H2, HU-A4H3, HU-A4H4, HU-A4H5, HU-A4H6, HU-A4H8, HU-A4HB, HU-A4HD, IN-7806, IN-7808, IN-8462, IN-8663, IN-A6RI, IN-A6RJ, IN-A6RL, IN-A6RN, IN-A6RO, IN-A6RP, IN-A6RR, IP-7968, KB-A6F5, KB-A6F7, MX-A5UG, MX-A5UJ, MX-A663, MX-A666, R5-A7O7, RD-A7BS, RD-A7BT, RD-A7BW, RD-A7C1.
⧫ **Glioblastoma Multiforme** (359 samples):
• Source P2 = [62]. Sample IDs are of the form Br*, where * is:
  001X, 018X, 019X, 02X, 03X, 04X, 05X, 06X, 07X, 08X, 102X, 103X, 104X, 10P, 112X, 116X, 117X, 118X, 11P, 128X, 12P, 132X, 133X, 136X, 13X, 143X, 148X, 14X, 15X, 16X, 17X, 20P, 21PT, 229T, 230T, 237T, 238T, 23X, 247T, 248T, 25X, 26X, 27P, 29P, 301T, 302T, 303T, 306T, 401X, 9PT.
• Source T11 = TCGA (see Acknowledgments (main text)). Sample IDs are of the form TCGA-*, where * is:
  02-0003, 02-0007, 02-0010, 02-0014, 02-0015, 02-0021, 02-0028, 02-0033, 02-0043, 02-0047, 02-0055, 02-0083, 02-0089, 02-0099, 02-0107, 02-0114, 02-0115, 02-2470, 02-2483, 02-2485, 02-2486, 06-0119, 06-0122, 06-0124, 06-0125, 06-0126, 06-0128, 06-0129, 06-0130, 06-0132, 06-0137, 06-0138, 06-0139, 06-0140, 06-0141, 06-0142, 06-0143, 06-0145, 06-0147, 06-0148, 06-0151, 06-0152, 06-0154, 06-0155, 06-0157, 06-0158, 06-0165, 06-0166, 06-0167, 06-0168, 06-0169, 06-0171, 06-0173, 06-0174, 06-0176, 06-0178, 06-0184, 06-0185, 06-0188, 06-0189, 06-0190, 06-0192, 06-0195, 06-0201, 06-0209, 06-0210, 06-0211, 06-0213, 06-0214, 06-0216, 06-0219, 06-0221, 06-0237, 06-0238, 06-0240, 06-0241, 06-0644, 06-0645, 06-0646, 06-0648, 06-0649, 06-0650, 06-0686, 06-0743, 06-0744, 06-0745, 06-0747, 06-0749, 06-0750, 06-0875, 06-0876, 06-0877, 06-0878, 06-0879, 06-0881, 06-0882, 06-0939, 06-1804, 06-1806, 06-2557, 06-2558, 06-2559, 06-2561, 06-2562, 06-2563, 06-2564, 06-2565, 06-2567, 06-2569, 06-2570, 06-5408, 06-5410, 06-5411, 06-5412, 06-5413, 06-5414, 06-5415, 06-5417, 06-5418, 06-5856, 06-5858, 06-5859, 06-6388, 06-6389, 06-6390, 06-6391, 06-6693, 06-6694, 06-6695, 06-6697, 06-6698, 06-6699, 06-6700, 06-6701, 08-0244, 08-0345, 08-0352, 08-0353, 08-0360, 08-0373, 08-0375, 08-0385, 08-0386, 12-0615, 12-0616, 12-0618, 12-0619, 12-0688, 12-0692, 12-0821, 12-1597, 12-3649, 12-3650, 12-3652, 12-3653, 12-5295, 12-5299, 12-5301, 14-0740, 14-0781, 14-0786, 14-0787, 14-0789, 14-0790, 14-0813, 14-0817, 14-0862, 14-0871, 14-1034, 14-1043, 14-1395, 14-1450, 14-1456, 14-1823, 14-1825, 14-1829, 14-2554, 14-3476, 14-4157, 15-0742, 15-1444, 16-0846, 16-0861, 16-1045, 16-1048, 19-1390, 19-1790, 19-2619, 19-2620, 19-2623, 19-2624, 19-2625, 19-2629, 19-2631, 19-4068, 19-5953, 26-1439, 26-1442, 26-5132, 26-5133, 26-5134, 26-5135, 26-5136, 26-5139, 26-6173, 26-6174, 27-1830, 27-1831, 27-1832, 27-1833, 27-1834, 27-1835, 27-1836, 27-1837, 27-1838, 27-2518, 27-2519, 27-2521, 27-2523, 27-2524, 27-2526, 27-2527, 27-2528, 28-1747, 28-1753, 28-2499, 28-2501, 28-2502, 28-2509, 28-2510, 28-2513, 28-2514, 28-5204, 28-5207, 28-5208, 28-5209, 28-5211, 28-5213, 28-5214, 28-5215, 28-5216, 28-5218, 28-5219, 28-5220, 28-6450, 32-1970, 32-1977, 32-1979, 32-1980, 32-1982, 32-1986, 32-1991, 32-2491, 32-2494, 32-2495, 32-2498, 32-2615, 32-2632, 32-2634, 32-2638, 32-4208, 32-4209, 32-4210, 32-4211, 32-4213, 32-4719, 32-5222, 41-2571, 41-2572, 41-2573, 41-2575, 41-3392, 41-3393, 41-3915, 41-4097, 41-5651, 41-6646, 74-6573, 74-6575, 74-6577, 74-6578, 74-6584, 76-4925, 76-4926, 76-4927, 76-4928, 76-4929, 76-4931, 76-4932, 76-4934, 76-4935, 76-6191, 76-6192, 76-6193, 76-6280, 76-6282, 76-6283, 76-6285, 76-6286, 76-6656, 76-6657, 76-6660, 76-6661, 76-6662, 76-6663, 76-6664, 81-5910, 81-5911, 87-5896.
⧫ **Head and Neck Cancer** (591 samples):
• Source A1 = [63]:
  139, 266, 325, 347, 388, 478, 91.
• Source S4 = [64]:
  HN12PT, HN22PT, HN27PT, HN32PT, HN33PT.
  Remaining sample IDs are of the form HN_*-Tumor, where * is:
  0-046, 0-064, 00076, 00122, 00190, 00313, 00338, 00361, 00378, 00443, 00466, 00761, 01000, 62237, 62298, 62318, 62338, 62374, 62415, 62417, 62421, 62426, 62469, 62481, 62493, 62505, 62506, 62515, 62532, 62539, 62601, 62602, 62624, 62646, 62652, 62671, 62672, 62686, 62699, 62739, 62740, 62741, 62755, 62756, 62807, 62814, 62825, 62832, 62854, 62857_2, 62860, 62861, 62863, 62897, 62921, 62926, 62984, 62996, 63007, 63021, 63027, 63039, 63048, 63058, 63080, 63081, 63095, 63114.
• Source T12 = TCGA (see Acknowledgments (main text)). Sample IDs are of the form TCGA-*, where * is:
  BA-4074, BA-4075, BA-4076, BA-4077, BA-4078, BA-5149, BA-5151, BA-5152, BA-5153, BA-5555, BA-5556, BA-5557, BA-5558, BA-5559, BA-6868, BA-6869, BA-6870, BA-6871, BA-6872, BA-6873, BA-7269, BA-A4IF, BA-A4IG, BA-A4IH, BA-A4II, BA-A6D8, BA-A6DA, BA-A6DB, BA-A6DD, BA-A6DE, BA-A6DF, BA-A6DG, BA-A6DI, BA-A6DJ, BA-A6DL, BB-4217, BB-4223, BB-4224, BB-4225, BB-4227, BB-4228, BB-7861, BB-7862, BB-7863, BB-7864, BB-7866, BB-7870, BB-7871, BB-7872, BB-8596, BB-8601, BB-A5HU, BB-A5HY, BB-A5HZ, BB-A6UM, BB-A6UO, C9-A47Z, C9-A480, CN-4723, CN-4725, CN-4726, CN-4727, CN-4728, CN-4729, CN-4730, CN-4731, CN-4733, CN-4734, CN-4735, CN-4736, CN-4737, CN-4738, CN-4739, CN-4740, CN-4741, CN-4742, CN-5355, CN-5356, CN-5358, CN-5359, CN-5360, CN-5361, CN-5363, CN-5364, CN-5365, CN-5366, CN-5367, CN-5369, CN-5370, CN-5373, CN-5374, CN-6010, CN-6011, CN-6012, CN-6013, CN-6016, CN-6017, CN-6018, CN-6019, CN-6020, CN-6021, CN-6022, CN-6023, CN-6024, CN-6988, CN-6989, CN-6992, CN-6994, CN-6995, CN-6996, CN-6997, CN-6998, CN-A497, CN-A498, CN-A499, CN-A49A, CN-A49B, CN-A49C, CN-A63T, CN-A63U, CN-A63V, CN-A63W, CN-A63Y, CN-A640, CN-A641, CN-A642, CN-A6UY, CN-A6V1, CN-A6V3, CN-A6V6, CN-A6V7, CQ-5323, CQ-5324, CQ-5325, CQ-5326, CQ-5327, CQ-5329, CQ-5330, CQ-5331, CQ-5332, CQ-5333, CQ-5334, CQ-6218, CQ-6219, CQ-6220, CQ-6221, CQ-6222, CQ-6223, CQ-6224, CQ-6225, CQ-6227, CQ-6228, CQ-6229, CQ-7063, CQ-7064, CQ-7065, CQ-7067, CQ-7068, CQ-7069, CQ-7071, CQ-7072, CQ-A4C6, CQ-A4C7, CQ-A4C9, CQ-A4CB, CQ-A4CD, CQ-A4CE, CQ-A4CG, CQ-A4CH, CQ-A4CI, CR-5243, CR-5247, CR-5248, CR-5249, CR-5250, CR-6467, CR-6470, CR-6471, CR-6472, CR-6473, CR-6474, CR-6477, CR-6478, CR-6480, CR-6481, CR-6482, CR-6484, CR-6487, CR-6488, CR-6491, CR-6492, CR-6493, CR-7364, CR-7365, CR-7367, CR-7368, CR-7369, CR-7370, CR-7371, CR-7372, CR-7373, CR-7374, CR-7376, CR-7377, CR-7379, CR-7380, CR-7382, CR-7383, CR-7385, CR-7386, CR-7388, CR-7389, CR-7390, CR-7391, CR-7392, CR-7393, CR-7394, CR-7395, CR-7397, CR-7398, CR-7399, CR-7401, CR-7402, CR-7404, CV-5430, CV-5431, CV-5432, CV-5434, CV-5435, CV-5436, CV-5439, CV-5440, CV-5441, CV-5442, CV-5443, CV-5444, CV-5966, CV-5970, CV-5971, CV-5973, CV-5976, CV-5977, CV-5978, CV-5979, CV-6003, CV-6433, CV-6436, CV-6441, CV-6933, CV-6934, CV-6935, CV-6936, CV-6937, CV-6938, CV-6939, CV-6940, CV-6941, CV-6942, CV-6943, CV-6945, CV-6948, CV-6950, CV-6951, CV-6952, CV-6953, CV-6954, CV-6955, CV-6956, CV-6959, CV-6960, CV-6961, CV-6962, CV-7089, CV-7090, CV-7091, CV-7095, CV-7097, CV-7099, CV-7100, CV-7101, CV-7102, CV-7103, CV-7104, CV-7177, CV-7178, CV-7180, CV-7183, CV-7235, CV-7236, CV-7238, CV-7242, CV-7243, CV-7245, CV-7247, CV-7248, CV-7250, CV-7252, CV-7253, CV-7254, CV-7255, CV-7261, CV-7263, CV-7406, CV-7407, CV-7409, CV-7410, CV-7411, CV-7413, CV-7414, CV-7415, CV-7416, CV-7418, CV-7421, CV-7422, CV-7423, CV-7424, CV-7425, CV-7427, CV-7429, CV-7430, CV-7432, CV-7433, CV-7434, CV-7435, CV-7437, CV-7438, CV-7440, CV-7446, CV-7568, CV-A45O, CV-A45P, CV-A45Q, CV-A45R, CV-A45T, CV-A45U, CV-A45V, CV-A45W, CV-A45X, CV-A45Y, CV-A45Z, CV-A460, CV-A461, CV-A463, CV-A464, CV-A465, CV-A468, CV-A6JD, CV-A6JE, CV-A6JM, CV-A6JN, CV-A6JO, CV-A6JT, CV-A6JU, CV-A6JY, CV-A6JZ, CV-A6K0, CV-A6K1, CV-A6K2, CX-7082, CX-7085, CX-7086, CX-7219, CX-A4AQ, D6-6515, D6-6516, D6-6517, D6-6823, D6-6824, D6-6825, D6-6826, D6-6827, D6-8568, D6-8569, D6-A4Z9, D6-A4ZB, D6-A6EK, D6-A6EM, D6-A6EN, D6-A6EO, D6-A6EP, D6-A6EQ, D6-A6ES, D6-A74Q, DQ-5624, DQ-5625, DQ-5629, DQ-5630, DQ-5631, DQ-7588, DQ-7589, DQ-7590, DQ-7591, DQ-7592, DQ-7593, DQ-7594, DQ-7595, DQ-7596, F7-7848, F7-8489, F7-A50G, F7-A50I, F7-A50J, F7-A61S, F7-A61V, F7-A61W, F7-A620, F7-A622, F7-A623, F7-A624, H7-7774, H7-8501, H7-A6C4, H7-A6C5, H7-A76A, HD-7229, HD-7753, HD-7754, HD-7831, HD-7832, HD-7917, HD-8224, HD-8314, HD-8634, HD-8635, HD-A4C1, HD-A633, HD-A634, HD-A6HZ, HD-A6I0, HL-7533, IQ-7630, IQ-7631, IQ-7632, IQ-A61E, IQ-A61G, IQ-A61H, IQ-A61I, IQ-A61J, IQ-A61K, IQ-A61L, IQ-A61O, IQ-A6SG, IQ-A6SH, KU-A66S, KU-A66T, KU-A6H7, KU-A6H8, MT-A51W, MT-A51X, MT-A67A, MT-A67D, MT-A67F, MT-A67G, MT-A7BN, MZ-A5BI, MZ-A6I9, MZ-A7D7, P3-A5Q6, P3-A5QA, P3-A5QE, P3-A5QF, P3-A6SW, P3-A6SX, P3-A6T0, P3-A6T2, P3-A6T3, P3-A6T4, P3-A6T5, P3-A6T6, P3-A6T7, P3-A6T8, QK-A64Z, QK-A652, QK-A6IF, QK-A6IG, QK-A6IH, QK-A6II, QK-A6IJ, QK-A6V9, QK-A6VB, QK-A6VC, RS-A6TO, RS-A6TP, T2-A6WX, T2-A6WZ, T2-A6X0, T2-A6X2, TN-A7HI, TN-A7HJ, TN-A7HL, UF-A718, UF-A719, UF-A71A, UF-A71B, UF-A71D, UF-A71E, UF-A7J9, UF-A7JA, UF-A7JC, UF-A7JD, UF-A7JF, UF-A7JH, UF-A7JJ, UF-A7JK, UF-A7JO, UF-A7JS, UF-A7JT, UF-A7JV, UP-A6WW, WA-A7GZ, WA-A7H4.
⧫ **Liver Cancer** (452 samples):
• Source S5 = [40]. Sample IDs are of the form BCB*, where * is:
  109T, 111T, 151T, 157T, 167T, 231T, 301T, 307T, 325T.
  Additional sample IDs are of the form BCM*, where * is:
  229T, 257T, 265T, 269T, 275T, 321T, 325T, 329T, 337T, 339T, 371T, 375T, 397T, 399T, 423T, 439T, 455T, 483T, 489T, 501T, 529T, 531T, 543T, 545T, 565T, 567T, 617T, 643T, 671T, 683T, 689T, 695T, 703T, 711T, 723T, 735T, 739T, 759T, 769T, 783T, 791T.
  Remaining sample IDs are of the form CHC*, where * is:
  051T, 059T, 060T, 097T, 1010T, 1028T, 1035T, 1040T, 1041T, 1044T, 1052T, 1053T, 1055T, 1060T, 1061T, 1062T, 1065T, 1079T, 1081T, 1082T, 1083T, 1085T, 1089T, 1091T, 1097T, 1098T, 1137T, 1148T, 1152T, 1154T, 1162T, 1177T, 1180T, 1182T, 1183T, 1185T, 1186T, 1190T, 1191T, 1192T, 1201T, 1205T, 1207T, 1209T, 1210T, 1211T, 121T, 1530T, 1531T, 1534T, 1539T, 1545T, 1556T, 155T, 1566T, 1568T, 1569T, 1591T, 1592T, 1594T, 1595T, 1596T, 1597T, 1598T, 1600T, 1601T, 1602T, 1603T, 1604T, 1611T, 1616T, 1624T, 1626T, 1629T, 1700T, 1704T, 1708T, 1712T, 1714T, 1715T, 1717T, 1719T, 1720T, 1725T, 1731T, 1732T, 1734T, 1736T, 1737T, 1738T, 1739T, 1741T, 1742T, 1743T, 1744T, 1745T, 1746T, 1747T, 1749T, 1750T, 1751T, 1753T, 1754T, 1756T, 1757T, 1763T, 1774T, 1775T, 1915T, 197T, 2029T, 2034T, 2039Tbis, 2043T, 2048T, 2052T, 205T, 2098T, 2099T, 2103T, 2110Tbis, 2111T, 2112T, 2113T, 2115T, 2127T, 2128T, 2134T, 2141T, 218T, 2200T, 2202T, 2206T, 2208T, 2211T, 2213T, 2215T, 2216T, 2321T, 2351T, 2352T, 2358T, 2362T, 253T, 258T, 301T, 302T, 303T, 304T, 306T, 307T, 313T, 314T, 320T, 322T, 326T, 327T, 361TA, 429T, 432T, 433T, 434T, 437T, 451T, 465T, 469T, 510T, 609T, 614T, 703T, 734T, 736T, 789T, 793T, 794T, 796T, 798T, 799T, 801T, 805T, 879T, 884T, 889T, 891T, 892T, 896T, 898T, 902T, 909T, 912T, 917T, 923T, 961T.
• Source H2 = [65]:
  P47, P48, P51, P52, P55, P56, P929.
• Source T13 = TCGA (see Acknowledgments (main text)). Sample IDs are of the form TCGA-*, where * is:
  BC-4073, BC-A10Q, BC-A10R, BC-A10S, BC-A10T, BC-A10U, BC-A10W, BC-A10X, BC-A10Y, BC-A10Z, BC-A110, BC-A112, BC-A216, BC-A217, BC-A3KF, BC-A3KG, BC-A5W4, BC-A69H, BC-A69I, BD-A2L6, BD-A3EP, BD-A3ER, BW-A5NO, BW-A5NP, BW-A5NQ, CC-5258, CC-5259, CC-5260, CC-5261, CC-5262, CC-5263, CC-5264, CC-A123, CC-A1HT, CC-A3M9, CC-A3MA, CC-A3MB, CC-A3MC, CC-A5UC, CC-A5UD, CC-A5UE, CC-A7IF, CC-A7IG, CC-A7IH, CC-A7II, CC-A7IJ, CC-A7IK, CC-A7IL, DD-A113, DD-A114, DD-A115, DD-A116, DD-A118, DD-A119, DD-A11A, DD-A11B, DD-A11C, DD-A11D, DD-A1E9, DD-A1EA, DD-A1EB, DD-A1EC, DD-A1ED, DD-A1EF, DD-A1EG, DD-A1EH, DD-A1EI, DD-A1EJ, DD-A1EK, DD-A1EL, DD-A39V, DD-A39W, DD-A39X, DD-A39Y, DD-A39Z, DD-A3A0, DD-A3A1, DD-A3A2, DD-A3A3, DD-A3A4, DD-A3A5, DD-A3A6, DD-A3A7, DD-A3A8, DD-A3A9, DD-A4NA, DD-A4NB, DD-A4ND, DD-A4NE, DD-A4NF, DD-A4NG, DD-A4NH, DD-A4NI, DD-A4NJ, DD-A4NK, DD-A4NL, DD-A4NN, DD-A4NO, DD-A4NP, DD-A4NQ, DD-A4NR, DD-A4NS, DD-A4NV, DD-A73A, DD-A73B, DD-A73C, DD-A73D, DD-A73E, DD-A73F, DD-A73G, ED-A459, ED-A4XI, ED-A5KG, ED-A627, ED-A66X, ED-A66Y, ED-A7PX, ED-A7PY, ED-A7PZ, ED-A7XO, ED-A7XP, ED-A82E, EP-A12J, EP-A26S, EP-A2KA, EP-A2KB, EP-A2KC, EP-A3JL, EP-A3RK, ES-A2HS, ES-A2HT, FV-A23B, FV-A2QQ, FV-A2QR, FV-A3I0, FV-A3I1, FV-A3R2, FV-A3R3, FV-A495, FV-A496, FV-A4ZP, FV-A4ZQ, G3-A25S, G3-A25T, G3-A25U, G3-A25V, G3-A25W, G3-A25Y, G3-A25Z, G3-A3CG, G3-A3CH, G3-A3CI, G3-A3CJ, G3-A3CK, G3-A5SI, G3-A5SJ, G3-A5SK, G3-A5SL, G3-A5SM, G3-A6UC, G3-A7M5, G3-A7M6, G3-A7M7, G3-A7M8, G3-A7M9, GJ-A6C0, HP-A5MZ, HP-A5N0, K7-A5RF, K7-A5RG, K7-A6G5, KR-A7K0, KR-A7K2, KR-A7K7, KR-A7K8, LG-A6GG, MI-A75C, MI-A75E, MI-A75G, MI-A75H, MI-A75I, MR-A520, NI-A4U2, O8-A75V, PD-A5DF, QA-A7B7, RC-A6M3, RC-A6M4, RC-A6M5, RC-A6M6, RC-A7S9, RC-A7SB, RC-A7SF, RC-A7SK, RG-A7D4, T1-A6J8, UB-A7MA, UB-A7MB, UB-A7MC, UB-A7MD, UB-A7ME, UB-A7MF.
⧫ **Lung Cancer** (1018 samples):
• Source D3 = [66]:
  16600, 16608, 16628, 16632, 16648, 16660, 16668, 16678, 16686, 16724, 16802, 16814, 16835, 16857, 16949, 17042, 17055, 17156, 17174, 17210, 17218, 17226, 17242, 17268, 17290, 17308, 17733, 17746, 17759, 17763.
• Source R1 = [67]:
  113368, 134398, 134413, 134417, 134421, 134426, 134427, 134430, 2334187, 2334188, 2334189, 2334191, 2334193, 2334195, 2334196, 2334199, 2334201, 2334202, 585203, 585205, 585208, 585210, 585223, 585258, 585260, 585265, 585267, 585270, 585272, 585276, 631052, 631056, 631060, 631064, 631076, 631084, 631092, 98687, 98711, 98735.
• Source P3 = [68]:
  H1672, H2171, S00022, S00050, S00356, S00472, S00501, S00539, S00827, S00830, S00833, S00836, S00837, S00841, S00932, S00933, S00935, S00936, S00943, S00944, S00945, S00946, S00947, S01366, S01453, S01494, S01512, S01563, S01728.
• Source S6 = [69]. Sample IDs are of the form LC_*, where * is:
  C1, C10, C11, C13, C14, C15, C17, C18, C19, C2, C20, C21, C22, C23, C24, C25, C26, C27, C28, C29, C30, C32, C33, C34, C35, C36, C4, C5, C6, C7, C8, C9, S10, S11, S12, S13, S14, S15, S16, S17, S18, S19, S2, S20, S21, S23, S24, S25, S27, S28, S29, S3, S31, S32, S34, S35, S37, S38, S39, S4, S40, S41, S42, S43, S44, S45, S46, S47, S48, S49, S5, S51, S6, S8, S9.
• Source I1 = [70]. Sample IDs are of the form LUAD.**.Tumor, where ** is (below * stands for NYU, e.g., *1021 = NYU1021 and the full sample ID is LUAD.NYU1021.Tumor):
  5O6B5, 74TBW, B00416, B00523, B00859, B00915, B01102, B01145, B01811, B01970, B02077, B02216, B02477, B02515, B02594, D00147, D01278, D01603, D01751, D02085, D02185, E00163, E00443, E00897, E00918, F00018, F00057, F00089, F00121, F00134, F00162, F00170, F00257, F00282, F00365, F00368, GU4I3, LC15C, LIP77, *1021, *1026, *1027, *1051S, *1093, *1096, *1101, *1142, *1177, *1195, *1210, *1219, *160, *184, *195, *201, *213, *252, *259, *263, *282, *284, *287, *315, *330, *408, *508, *574S, *575, *584S, *608, *627, *669, *689, *696, *704, *739, *796, *802, *803, *846, *847, *848, *947, *994, QCHM7, QJN9L, S00484, S00486, S00499, S01304, S01306, S01315, S01320, S01354, S01357, S01362, S01373, S01409, S01413, S01482, TLLGS, UF7HM, VUMN6, YINHD, YKER9.
  Additional sample IDs are of the form LUAD.CHTN.*.Tumor, where * is:
  3090346, 3090415, 3090416, 4090680, MAD04.00674, MAD06.00490, MAD06.00668, MAD06.00678, MAD08.00104, Z4716A.
  Further sample IDs are of the form LUAD.RT.*.Tumor, where * is:
  S01477, S01487, S01699, S01700, S01702, S01703, S01709, S01711, S01721, S01769, S01770, S01771, S01774, S01777, S01808, S01810, S01813, S01818, S01831, S01832, S01840, S01852, S01856, S01866.
  Remaining sample IDs are of the form LUAD_*.Tumor, where * is:
  E00522, E00565, E00623, E00703, E00945, E01047, E01086, E01147, E01166, E01319, E01419.
• Source T14 = TCGA (see Acknowledgments (main text)). Sample IDs are of the form TCGA.*, where * is:
  05.4244, 05.4249, 05.4250, 05.4382, 05.4384, 05.4389, 05.4390, 05.4395, 05.4396, 05.4397, 05.4398, 05.4402, 05.4403, 05.4405, 05.4410, 05.4415, 05.4417, 05.4418, 05.4420, 05.4422, 05.4424, 05.4425, 05.4426, 05.4427, 05.4430, 05.4432, 05.4433, 05.4434, 05.5420, 05.5423, 05.5425, 05.5428, 05.5429, 05.5715, 17.Z000, 17.Z001, 17.Z002, 17.Z003, 17.Z004, 17.Z005, 17.Z007, 17.Z008, 17.Z009, 17.Z010, 17.Z011, 17.Z012, 17.Z013, 17.Z014, 17.Z015, 17.Z016, 17.Z017, 17.Z018, 17.Z019, 17.Z020, 17.Z021, 17.Z022, 17.Z023, 17.Z025, 17.Z026, 17.Z027, 17.Z028, 17.Z030, 17.Z031, 17.Z032, 17.Z033, 17.Z035, 17.Z036, 17.Z037, 17.Z040, 17.Z041, 17.Z042, 17.Z043, 17.Z044, 17.Z045, 17.Z046, 17.Z047, 17.Z048, 17.Z049, 17.Z050, 17.Z051, 17.Z052, 17.Z053, 17.Z054, 17.Z055, 17.Z056, 17.Z057, 17.Z058, 17.Z059, 17.Z060, 17.Z061, 17.Z062, 18.3406, 18.3407, 18.3408, 18.3409, 18.3410, 18.3411, 18.3412, 18.3414, 18.3415, 18.3416, 18.3417, 18.3419, 18.3421, 18.4083, 18.4086, 18.4721, 18.5592, 18.5595, 21.1070, 21.1071, 21.1076, 21.1077, 21.1078, 21.1081, 21.5782, 21.5784, 21.5786, 21.5787, 22.0944, 22.1002, 22.1011, 22.1012, 22.1016, 22.4591, 22.4593, 22.4595, 22.4599, 22.4601, 22.4604, 22.4607, 22.4613, 22.5471, 22.5472, 22.5473, 22.5474, 22.5477, 22.5478, 22.5480, 22.5482, 22.5485, 22.5489, 22.5491, 22.5492, 33.4532, 33.4533, 33.4538, 33.4547, 33.4566, 33.4582, 33.4583, 33.4586, 33.6737, 34.2596, 34.2600, 34.2608, 34.5231, 34.5232, 34.5234, 34.5236, 34.5239, 34.5240, 34.5927, 34.5928, 34.5929, 35.3615, 35.3621, 35.4122, 35.4123, 35.5375, 37.3783, 37.3789, 37.4133, 37.4135, 37.4141, 37.5819, 38.4625, 38.4626, 38.4627, 38.4628, 38.4629, 38.4630, 38.4631, 38.4632, 38.6178, 38.7271, 38.A44F, 39.5016, 39.5019, 39.5021, 39.5022, 39.5024, 39.5027, 39.5028, 39.5029, 39.5030, 39.5031, 39.5035, 39.5036, 39.5037, 39.5039, 43.2578, 43.3394, 43.3920, 43.5668, 43.6143, 43.6647, 43.6770, 43.6771, 44.2655, 44.2656, 44.2657, 44.2659, 44.2661, 44.2662, 44.2665, 44.2666, 44.2668, 44.3396, 44.3398, 44.3918, 44.3919, 44.4112, 44.5643, 44.5644, 44.5645, 44.6144, 44.6145, 44.6146, 44.6147, 44.6148, 44.6774, 44.6775, 44.6776, 44.6777, 44.6778, 44.6779, 44.7659, 44.7660, 44.7661, 44.7662, 44.7667, 44.7669, 44.7670, 44.7671, 44.7672, 44.8117, 44.8119, 44.8120, 44.A479, 44.A47A, 44.A47B, 44.A47F, 44.A47G, 44.A4SS, 44.A4SU, 46.3765, 46.3767, 46.3768, 46.3769, 46.6025, 46.6026, 49.4486, 49.4487, 49.4488, 49.4490, 49.4494, 49.4501, 49.4505, 49.4506, 49.4507, 49.4510, 49.4512, 49.4514, 49.6742, 49.6743, 49.6744, 49.6745, 49.6761, 49.6767, 50.5044, 50.5045, 50.5049, 50.5051, 50.5055, 50.5066, 50.5068, 50.5072, 50.5930, 50.5931, 50.5932, 50.5933, 50.5935, 50.5936, 50.5939, 50.5941, 50.5942, 50.5944, 50.5946, 50.6590, 50.6591, 50.6592, 50.6593, 50.6594, 50.6595, 50.6597, 50.6673, 50.7109, 50.8457, 50.8459, 50.8460, 51.4079, 51.4080, 51.4081, 53.7624, 53.7626, 53.7813, 53.A4EZ, 55.1592, 55.1594, 55.1595, 55.1596, 55.5899, 55.6543, 55.6642, 55.6712, 55.6968, 55.6969, 55.6970, 55.6971, 55.6972, 55.6975, 55.6978, 55.6979, 55.6980, 55.6981, 55.6982, 55.6983, 55.6984, 55.6985, 55.6986, 55.6987, 55.7227, 55.7281, 55.7283, 55.7284, 55.7570, 55.7573, 55.7574, 55.7576, 55.7724, 55.7725, 55.7726, 55.7727, 55.7728, 55.7815, 55.7816, 55.7903, 55.7907, 55.7910, 55.7911, 55.7913, 55.7914, 55.7994, 55.7995, 55.8085, 55.8087, 55.8089, 55.8090, 55.8091, 55.8092, 55.8094, 55.8096, 55.8097, 55.8203, 55.8204, 55.8205, 55.8206, 55.8207, 55.8208, 55.8299, 55.8301, 55.8302, 55.8505, 55.8506, 55.8507, 55.8508, 55.8510, 55.8511, 55.8512, 55.8513, 55.8514, 55.8614, 55.8615, 55.8616, 55.8619, 55.8620, 55.8621, 55.A48X, 55.A48Y, 55.A48Z, 55.A490, 55.A491, 55.A492, 55.A493, 55.A494, 55.A4DF, 55.A4DG, 56.1622, 56.5897, 56.5898, 56.6545, 56.6546, 60.2698, 60.2707, 60.2708, 60.2709, 60.2710, 60.2711, 60.2712, 60.2713, 60.2715, 60.2719, 60.2720, 60.2721, 60.2722, 60.2723, 60.2724, 60.2725, 60.2726, 62.8394, 62.8395, 62.8397, 62.8398, 62.8399, 62.8402, 62.A46O, 62.A46P, 62.A46R, 62.A46S, 62.A46U, 62.A46V, 62.A46Y, 62.A470, 62.A471, 62.A472, 63.5128, 63.5131, 63.6202, 64.1676, 64.1677, 64.1678, 64.1679, 64.1680, 64.1681, 64.5774, 64.5775, 64.5778, 64.5779, 64.5781, 64.5815, 66.2727, 66.2734, 66.2742, 66.2744, 66.2754, 66.2755, 67.3770, 67.3771, 67.3772, 67.3773, 67.3774, 67.4679, 67.6215, 67.6216, 67.6217, 69.7760, 69.7761, 69.7763, 69.7764, 69.7765, 69.7973, 69.7974, 69.7978, 69.7979, 69.7980, 69.8253, 69.8254, 69.8255, 69.A59K, 71.6725, 71.8520, 73.4658, 73.4659, 73.4662, 73.4666, 73.4668, 73.4670, 73.4675, 73.4676, 73.4677, 73.7498, 73.7499, 75.5122, 75.5125, 75.5126, 75.5146, 75.5147, 75.6203, 75.6205, 75.6206, 75.6207, 75.6211, 75.6212, 75.6214, 75.7025, 75.7027, 75.7030, 75.7031, 78.7143, 78.7145, 78.7146, 78.7147, 78.7148, 78.7149, 78.7150, 78.7152, 78.7153, 78.7154, 78.7155, 78.7156, 78.7158, 78.7159, 78.7160, 78.7161, 78.7162, 78.7163, 78.7166, 78.7167, 78.7220, 78.7535, 78.7536, 78.7537, 78.7539, 78.7540, 78.7542, 78.7633, 78.8640, 78.8648, 78.8655, 78.8660, 78.8662, 80.5607, 80.5608, 80.5611, 83.5908, 86.6562, 86.6851, 86.7701, 86.7711, 86.7713, 86.7714, 86.7953, 86.7954, 86.7955, 86.8054, 86.8055, 86.8056, 86.8073, 86.8074, 86.8075, 86.8076, 86.8278, 86.8279, 86.8280, 86.8281, 86.8358, 86.8359, 86.8585, 86.8668, 86.8669, 86.8671, 86.8672, 86.8673, 86.8674, 86.A456, 86.A4D0, 86.A4JF, 86.A4P7, 86.A4P8, 91.6828, 91.6829, 91.6830, 91.6831, 91.6835, 91.6836, 91.6840, 91.6847, 91.6848, 91.6849, 91.7771, 91.8496, 91.8497, 91.8499, 91.A4BC, 91.A4BD, 93.7347, 93.7348, 93.8067, 93.A4JN, 93.A4JO, 93.A4JP, 93.A4JQ, 95.7039, 95.7043, 95.7562, 95.7567, 95.7944, 95.7947, 95.7948, 95.8039, 95.8494, 95.A4VK, 95.A4VN, 95.A4VP, 97.7546, 97.7547, 97.7552, 97.7553, 97.7554, 97.7937, 97.7938, 97.7941, 97.8171, 97.8172, 97.8174, 97.8175, 97.8176, 97.8177, 97.8179, 97.8547, 97.8552, 97.A4LX, 97.A4M0, 97.A4M1, 97.A4M2, 97.A4M3, 97.A4M5, 97.A4M6, 97.A4M7, 99.7458, 99.8025, 99.8028, 99.8032, 99.8033, J2.8192, J2.8194, J2.A4AD, J2.A4AE, J2.A4AG, L4.A4E5, L4.A4E6, L9.A443, L9.A444, MN.A4N1, MN.A4N4, MN.A4N5, MP.A4SV, MP.A4SW, MP.A4SY, MP.A4T2, MP.A4T4, MP.A4T6, MP.A4T7, MP.A4T8, MP.A4T9, MP.A4TA, MP.A4TC, MP.A4TD, MP.A4TE, MP.A4TF, MP.A4TH, MP.A4TI, MP.A4TK, MP.A5C7, NJ.A4YF, NJ.A4YG, NJ.A4YI, NJ.A4YP, NJ.A4YQ, NJ.A55A, NJ.A55O, NJ.A55R, O1.A52J.
⧫ **Melanoma** (594 samples):
• Source S7 = [71]:
  A02, A06, D05, D14, D35, D36, D41, D49.
• Source D4 = [72]:
  COLO-829.
• Source B1 = [73]. Sample IDs are of the form ME*-Tumor, where * is:
  001, 002, 009, 010, 011, 012, 014, 015, 016, 017, 018, 020, 021, 024, 029, 030, 032, 033, 034, 035, 037, 041, 043, 044, 045, 048, 049, 050.
  Remaining sample IDs are:
  Mel-BRAFi-03-Tumor, Mel_BRAFi_02_PRE-Tumor.
• Source A2 = [32]. Sample IDs are of the form PD*, where * is:
  10020a, 10021a, 10022a, 9024a2, 9024b, 9025a, 9025b, 9026a, 9027a, 9027b, 9028a, 9028b, 9029a, 9030a, 9031a, 9032a, 9033a.
• Source H3 = [74]. Sample IDs are of the form SKCM-*-Tumor, where * is:
  13447, 13456, 13463, 13468, 13473, 13531, 13537, 13543, 13549, 13560, 13561, 13567, 13575, 13591, 13600.
  Additional sample IDs are of the form SKCM-JWCI-*-Tumor, where * is:
  14, 27, WGS-1, WGS-11, WGS-12, WGS-13, WGS-15, WGS-18, WGS-19, WGS-2, WGS-20, WGS-21, WGS-22, WGS-23, WGS-24, WGS-25, WGS-26, WGS-29, WGS-3, WGS-32, WGS-33, WGS-34, WGS-35, WGS-36, WGS-37, WGS-38, WGS-39, WGS-4, WGS-42, WGS-43, WGS-5, WGS-6, WGS-7, WGS-8.
  Further sample IDs are of the form SKCM-Ma-Mel-*-Tumor, where * is:
  04, 05, 08a, 102, 103b, 105, 107, 108, 114, 119, 120, 122, 123, 15, 16, 19, 27, 28, 35, 36, 37, 48, 53, 54a, 55, 59, 62, 63, 65, 67, 71, 76, 79, 85, 86, 91, 92, 94.
  Remaining sample IDs are:
  SKCM-UKRV-Mel-20-Tumor, SKCM-UKRV-Mel-24-Tumor, SKCM-UKRV-Mel-6-Tumor.
• Source T15 = TCGA (see Acknowledgments (main text)). Sample IDs are of the form TCGA-*, where * is:
  BF-A1PU, BF-A1PV, BF-A1PX, BF-A1PZ, BF-A1Q0, BF-A3DJ, BF-A3DL, BF-A3DM, BF-A3DN, D3-A1Q1, D3-A1Q3, D3-A1Q4, D3-A1Q5, D3-A1Q6, D3-A1Q7, D3-A1Q8, D3-A1Q9, D3-A1QA, D3-A1QB, D3-A2J6, D3-A2J7, D3-A2J8, D3-A2J9, D3-A2JA, D3-A2JB, D3-A2JC, D3-A2JD, D3-A2JF, D3-A2JG, D3-A2JH, D3-A2JK, D3-A2JL, D3-A2JN, D3-A2JO, D3-A2JP, D3-A3BZ, D3-A3C1, D3-A3C3, D3-A3C6, D3-A3C7, D3-A3C8, D3-A3CB, D3-A3CC, D3-A3CE, D3-A3CF, D3-A3ML, D3-A3MO, D3-A3MR, D3-A3MU, D3-A3MV, D9-A148, D9-A149, D9-A1JW, D9-A1JX, D9-A1X3, DA-A1HV, DA-A1HW, DA-A1HY, DA-A1I0, DA-A1I1, DA-A1I2, DA-A1I4, DA-A1I5, DA-A1I7, DA-A1I8, DA-A1IA, DA-A1IB, DA-A1IC, DA-A3F3, DA-A3F5, DA-A3F8, EB-A1NK, EB-A24C, EB-A24D, EB-A299, EB-A3HV, EE-A17X, EE-A17Y, EE-A17Z, EE-A180, EE-A181, EE-A182, EE-A183, EE-A184, EE-A185, EE-A20B, EE-A20C, EE-A20F, EE-A20H, EE-A20I, EE-A29A, EE-A29B, EE-A29C, EE-A29D, EE-A29E, EE-A29G, EE-A29H, EE-A29L, EE-A29M, EE-A29N, EE-A29P, EE-A29Q, EE-A29R, EE-A29S, EE-A29T, EE-A29V, EE-A29W, EE-A29X, EE-A2A0, EE-A2A1, EE-A2A2, EE-A2A5, EE-A2A6, EE-A2GB, EE-A2GC, EE-A2GD, EE-A2GE, EE-A2GH, EE-A2GI, EE-A2GJ, EE-A2GK, EE-A2GL, EE-A2GM, EE-A2GN, EE-A2GO, EE-A2GP, EE-A2GR, EE-A2GS, EE-A2GT, EE-A2GU, EE-A2M5, EE-A2M6, EE-A2M7, EE-A2M8, EE-A2MC, EE-A2MD, EE-A2ME, EE-A2MF, EE-A2MG, EE-A2MH, EE-A2MI, EE-A2MJ, EE-A2MK, EE-A2ML, EE-A2MM, EE-A2MN, EE-A2MP, EE-A2MQ, EE-A2MR, EE-A2MS, EE-A2MT, EE-A2MU, EE-A3AA, EE-A3AB, EE-A3AC, EE-A3AD, EE-A3AE, EE-A3AF, EE-A3AG, EE-A3AH, EE-A3J3, EE-A3J4, EE-A3J5, EE-A3J7, EE-A3J8, EE-A3JA, EE-A3JB, EE-A3JD, EE-A3JE, EE-A3JH, EE-A3JI, ER-A193, ER-A194, ER-A195, ER-A196, ER-A197, ER-A198, ER-A199, ER-A19A, ER-A19B, ER-A19C, ER-A19D, ER-A19E, ER-A19F, ER-A19G, ER-A19H, ER-A19J, ER-A19K, ER-A19L, ER-A19N, ER-A19O, ER-A19P, ER-A19Q, ER-A19S, ER-A19T, ER-A1A1, ER-A2NB, ER-A2NC, ER-A2ND, ER-A2NE, ER-A2NF, ER-A2NG, ER-A2NH, ER-A3ES, ER-A3ET, ER-A3EV, FR-A2OS, FS-A1YX, FS-A1YY, FS-A1Z0, FS-A1Z3, FS-A1Z4, FS-A1Z7, FS-A1ZB, FS-A1ZC, FS-A1ZD, FS-A1ZE, FS-A1ZF, FS-A1ZG, FS-A1ZH, FS-A1ZJ, FS-A1ZK, FS-A1ZM, FS-A1ZN, FS-A1ZP, FS-A1ZQ, FS-A1ZR, FS-A1ZS, FS-A1ZT, FS-A1ZU, FS-A1ZW, FS-A1ZY, FS-A1ZZ, FW-A3I3, GF-A2C7, GN-A262, GN-A263, GN-A264, GN-A265, GN-A266, GN-A267, GN-A268, GN-A269, GN-A26A, GN-A26C, GN-A26D, HR-A2OG, HR-A2OH, IH-A3EA, D3-A5GT, D9-A3Z4, D9-A4Z2, D9-A4Z3, D9-A4Z5, EB-A3XB, EB-A3XC, EB-A3XD, EB-A3XE, EB-A3Y6, EB-A3Y7, EB-A41A, EB-A41B, EB-A42Y, EB-A42Z, EB-A430, EB-A431, EB-A44N, EB-A44O, EB-A44P, EB-A4IQ, EB-A4IS, EB-A4OY, EB-A4OZ, EB-A4P0, EB-A551, EB-A553, EB-A57M, EB-A5SE, EB-A5SF, EB-A5UM, FR-A3R1, FW-A5DX, BF-A5EO, BF-A5EP, BF-A5EQ, BF-A5ER, BF-A5ES, D3-A51E, D3-A51F, D3-A51G, D3-A51H, D3-A51J, D3-A51K, D3-A51N, D3-A51R, D3-A51T, D3-A5GL, D3-A5GN, D3-A5GO, D3-A5GR, D3-A5GS, D9-A3Z1, D9-A3Z3, D9-A6E9, D9-A6EA, D9-A6EC, D9-A6EG, DA-A3F2, EB-A3XF, EB-A44Q, EB-A44R, EB-A4XL, EB-A5FP, EB-A5KH, EB-A5SG, EB-A5SH, EB-A5UL, EB-A5UN, EB-A5VU, EB-A5VV, EB-A6L9, EB-A6QY, EB-A6QZ, EB-A6R0, ER-A19M, ER-A19W, ER-A3PL, ER-A42H, ER-A42K, ER-A42L, FR-A3YN, FR-A3YO, FR-A44A, FR-A69P, FR-A726, FR-A728, FS-A1YW, FS-A1ZA, FS-A4F4, FS-A4F5, FS-A4F8, FS-A4F9, FS-A4FB, FS-A4FC, FS-A4FD, FW-A3R5, FW-A3TU, FW-A3TV, FW-A5DY, GF-A3OT, GF-A6C8, GF-A6C9, GF-A769, GN-A4U3, GN-A4U4, GN-A4U5, GN-A4U7, GN-A4U8, GN-A4U9, OD-A75X, QB-A6FS, RP-A690, RP-A693, RP-A694, RP-A695, D3-A5GU, FS-A4F0, GF-A4EO, RZ-AB0B, V3-A9ZX, V3-A9ZY, V4-A9E5, V4-A9E7, V4-A9E8, V4-A9E9, V4-A9EA, V4-A9EC, V4-A9ED, V4-A9EE, V4-A9EF, V4-A9EH, V4-A9EI, V4-A9EJ, V4-A9EK, V4-A9EL, V4-A9EM, V4-A9EO, V4-A9EQ, V4-A9ES, V4-A9ET, V4-A9EU, V4-A9EV, V4-A9EW, V4-A9EX, V4-A9EY, V4-A9EZ, V4-A9F0, V4-A9F1, V4-A9F2, V4-A9F3, V4-A9F4, V4-A9F5, V4-A9F7, V4-A9F8, VD-A8K7, VD-A8K8, VD-A8K9, VD-A8KA, VD-A8KB, VD-A8KD, VD-A8KE, VD-A8KF, VD-A8KG, VD-A8KH, VD-A8KI, VD-A8KJ, VD-A8KK, VD-A8KL, VD-A8KM, VD-A8KN, VD-A8KO, VD-AA8M, VD-AA8N, VD-AA8O, VD-AA8P, VD-AA8Q, VD-AA8R, VD-AA8S, VD-AA8T, WC-A87T, WC-A87U, WC-A87W, WC-A87Y, WC-A880, WC-A881, WC-A882, WC-A883, WC-A884, WC-A885, WC-A888, WC-A88A, WC-AA9A, WC-AA9E, YZ-A980, YZ-A982, YZ-A983, YZ-A984, YZ-A985.
⧫ **Nasopharyngeal Cancer** (11 samples):
• Source L2 = [75]:
  NPC088D, NPC105D, NPC29F, NPC31F, NPC34F, NPC3F, NPC42F, NPC4D, NPC4F, NPC5D, NPC5F.
⧫ **Oral Cancer** (106 samples):
• Source I2 = [76]. Sample IDs are of the form OSCC-GB_0*, where * is:
  001011, 002011, 003011, 004011, 005011, 006011, 007011, 008011, 011011, 012011, 013011, 014011, 015011, 016011, 017011, 018011, 019011, 020011, 021011, 022011, 023011, 024011, 025011, 026011, 027011, 028011, 029011, 030011, 031011, 032011, 033011, 034011, 035011, 036011, 037011, 038011, 039011, 040011, 041011, 042011, 043011, 044011, 045011, 046011, 047011, 048011, 049011, 050011, 051011, 052011, 053011, 054011, 055011, 056011, 057011, 058011, 059011, 060011, 061011, 062011, 063011, 064011, 065011, 066011, 067011, 068011, 069011, 070011, 073011, 074011, 075011, 076011, 077011, 080011, 081011, 082011, 083011, 084011, 085011, 086011, 087011, 088011, 089011, 090011, 091011, 092011, 093011, 094011, 095011, 096011, 097011, 098011, 099011, 100011, 101011, 102011, 103011, 104011, 105011, 106011, 107011, 108011, 109011, 110011, 111011, 112011.
⧫ **Ovarian Cancer** (471 samples):
• Source J1 = [77]:
  OCC01PT, OCC02PT, OCC03PT, OCC04PT, OCC05PT, OCC06PT, OCC07PT, OCC08PT.
• Source T16 = TCGA (see Acknowledgments (main text)). Sample IDs are of the form TCGA-*, where * is:
  04-1331, 04-1332, 04-1336, 04-1337, 04-1338, 04-1342, 04-1343, 04-1346, 04-1347, 04-1348, 04-1349, 04-1350, 04-1351, 04-1353, 04-1356, 04-1357, 04-1361, 04-1362, 04-1364, 04-1365, 04-1367, 04-1369, 04-1514, 04-1516, 04-1517, 04-1519, 04-1525, 04-1530, 04-1542, 04-1638, 04-1644, 04-1646, 04-1648, 04-1649, 04-1651, 04-1652, 04-1655, 09-0364, 09-0365, 09-0366, 09-0367, 09-0369, 09-1659, 09-1661, 09-1662, 09-1664, 09-1665, 09-1666, 09-1669, 09-1670, 09-1672, 09-1673, 09-1674, 09-1675, 09-2044, 09-2045, 09-2049, 09-2050, 09-2051, 09-2053, 09-2056, 10-0926, 10-0927, 10-0928, 10-0930, 10-0931, 10-0933, 10-0934, 10-0935, 10-0937, 10-0938, 13-0714, 13-0717, 13-0720, 13-0723, 13-0724, 13-0726, 13-0727, 13-0730, 13-0751, 13-0755, 13-0758, 13-0760, 13-0761, 13-0762, 13-0765, 13-0791, 13-0792, 13-0793, 13-0795, 13-0800, 13-0801, 13-0804, 13-0807, 13-0883, 13-0884, 13-0885, 13-0886, 13-0887, 13-0889, 13-0890, 13-0891, 13-0893, 13-0894, 13-0897, 13-0899, 13-0900, 13-0901, 13-0903, 13-0904, 13-0905, 13-0906, 13-0910, 13-0911, 13-0912, 13-0913, 13-0916, 13-0919, 13-0920, 13-0923, 13-0924, 13-1403, 13-1404, 13-1405, 13-1407, 13-1408, 13-1409, 13-1410, 13-1411, 13-1412, 13-1477, 13-1481, 13-1482, 13-1483, 13-1484, 13-1487, 13-1488, 13-1489, 13-1491, 13-1492, 13-1494, 13-1495, 13-1496, 13-1497, 13-1498, 13-1499, 13-1501, 13-1504, 13-1505, 13-1506, 13-1507, 13-1509, 13-1510, 13-1512, 13-2057, 13-2059, 13-2060, 13-2061, 13-2065, 13-2066, 13-2071, 20-0987, 20-0990, 20-0991, 20-1682, 20-1683, 20-1684, 20-1685, 20-1686, 20-1687, 23-1021, 23-1022, 23-1023, 23-1024, 23-1026, 23-1027, 23-1028, 23-1029, 23-1030, 23-1031, 23-1032, 23-1109, 23-1110, 23-1111, 23-1114, 23-1116, 23-1117, 23-1118, 23-1119, 23-1120, 23-1122, 23-1123, 23-1124, 23-1809, 23-2072, 23-2077, 23-2078, 23-2079, 23-2081, 23-2641, 23-2643, 23-2645, 23-2647, 23-2649, 24-0966, 24-0968, 24-0970, 24-0975, 24-0979, 24-0980, 24-0982, 24-1103, 24-1104, 24-1105, 24-1413, 24-1416, 24-1417, 24-1418, 24-1419, 24-1422, 24-1423, 24-1424, 24-1425, 24-1426, 24-1427, 24-1428, 24-1431, 24-1434, 24-1435, 24-1436, 24-1463, 24-1464, 24-1466, 24-1469, 24-1470, 24-1471, 24-1474, 24-1544, 24-1545, 24-1546, 24-1548, 24-1549, 24-1551, 24-1552, 24-1553, 24-1555, 24-1556, 24-1557, 24-1558, 24-1560, 24-1562, 24-1563, 24-1564, 24-1565, 24-1567, 24-1603, 24-1604, 24-1614, 24-1616, 24-1842, 24-1843, 24-1844, 24-1845, 24-1846, 24-1847, 24-1849, 24-1850, 24-2019, 24-2024, 24-2030, 24-2035, 24-2038, 24-2254, 24-2260, 24-2261, 24-2262, 24-2267, 24-2271, 24-2280, 24-2281, 24-2288, 24-2289, 24-2290, 24-2293, 24-2298, 25-1313, 25-1315, 25-1316, 25-1317, 25-1318, 25-1319, 25-1320, 25-1321, 25-1322, 25-1324, 25-1325, 25-1326, 25-1328, 25-1329, 25-1623, 25-1625, 25-1626, 25-1627, 25-1628, 25-1630, 25-1631, 25-1632, 25-1633, 25-1634, 25-1635, 25-2042, 25-2391, 25-2392, 25-2393, 25-2396, 25-2398, 25-2399, 25-2400, 25-2401, 25-2404, 25-2408, 25-2409, 29-1688, 29-1690, 29-1691, 29-1693, 29-1694, 29-1695, 29-1696, 29-1697, 29-1698, 29-1699, 29-1701, 29-1702, 29-1703, 29-1705, 29-1707, 29-1710, 29-1711, 29-1761, 29-1762, 29-1763, 29-1764, 29-1766, 29-1768, 29-1769, 29-1770, 29-1771, 29-1774, 29-1775, 29-1776, 29-1777, 29-1778, 29-1781, 29-1783, 29-1784, 29-1785, 29-2427, 29-2429, 29-2431, 29-2432, 29-2434, 29-2436, 30-1714, 30-1718, 30-1853, 30-1855, 30-1856, 30-1857, 31-1950, 36-1568, 36-1569, 36-1570, 36-1571, 36-1574, 36-1575, 36-1576, 36-1577, 36-1578, 36-1580, 36-2530, 36-2532, 36-2533, 36-2534, 36-2537, 36-2538, 36-2539, 36-2540, 36-2542, 36-2543, 36-2544, 36-2545, 36-2547, 36-2548, 36-2551, 36-2552, 42-2582, 42-2587, 42-2588, 42-2589, 42-2590, 42-2591, 57-1582, 57-1584, 57-1586, 57-1993, 59-2348, 59-2350, 59-2351, 59-2352, 59-2354, 59-2355, 59-2363, 59-2372, 61-1722, 61-1725, 61-1727, 61-1728, 61-1730, 61-1733, 61-1736, 61-1737, 61-1738, 61-1740, 61-1741, 61-1895, 61-1899, 61-1900, 61-1901, 61-1903, 61-1904, 61-1906, 61-1907, 61-1910, 61-1911, 61-1913, 61-1914, 61-1915, 61-1995, 61-1998, 61-2000, 61-2002, 61-2003, 61-2008, 61-2009, 61-2012, 61-2016, 61-2092, 61-2094, 61-2095, 61-2097, 61-2101, 61-2102, 61-2104, 61-2109, 61-2110, 61-2111, 61-2113, 61-2610, 61-2611, 61-2612, 61-2613, 61-2614.
⧫ **Pancreatic Cancer** (184 samples):
• Source W2 = [78]:
  IPMN 11, IPMN 12, IPMN 20, IPMN 21, IPMN 36, IPMN 4, IPMN 41, MCN 162, MCN 163, MCN 164, MCN 166, MCN 168, MCN 169, MCN 170, SCA 14, SCA 23, SCA 27, SCA 35, SCA 37, SCA 38, SCA 40, SPN 8.
• Source J2 = [79]. Sample IDs are of the form PanNET*, where * is:
  10PT, 21PT, 23PT, 24PT, 25PT, 31PT, 36PT, 3PT, 7PT, 93PT.
• Source T17 = TCGA (see Acknowledgments (main text)). Sample IDs are of the form TCGA-*, where * is:
  2L-AAQA, 2L-AAQE, 2L-AAQI, 2L-AAQJ, 2L-AAQL, 2L-AAQM, 3A-A9I5, 3A-A9I7, 3A-A9I9, 3A-A9IB, 3A-A9IC, 3A-A9IH, 3A-A9IJ, 3A-A9IL, 3A-A9IN, 3A-A9IO, 3A-A9IR, 3A-A9IS, 3A-A9IU, 3E-AAAY, 3E-AAAZ, F2-6879, F2-6880, F2-7273, F2-7276, F2-A44G, F2-A44H, F2-A7TX, F2-A8YN, FB-A4P5, FB-A4P6, FB-A545, FB-A5VM, FB-A78T, FB-A7DR, FB-AAPS, FQ-6551, FQ-6552, FQ-6553, FQ-6554, FQ-6555, FQ-6558, FQ-6559, FZ-5919, FZ-5920, FZ-5921, FZ-5922, FZ-5923, FZ-5924, FZ-5926, H6-8124, H6-A45N, H8-A6C1, HV-A5A3, HV-A5A4, HV-A5A5, HV-A5A6, HV-A7OL, HV-A7OP, HV-AA8X, HZ-7289, HZ-7918, HZ-7919, HZ-7920, HZ-7922, HZ-7923, HZ-7924, HZ-7925, HZ-7926, HZ-8001, HZ-8002, HZ-8003, HZ-8005, HZ-8315, HZ-8317, HZ-8636, HZ-8637, HZ-8638, HZ-A49G, HZ-A49H, HZ-A49I, HZ-A4BH, HZ-A4BK, HZ-A77O, HZ-A77P, HZ-A77Q, HZ-A8P0, HZ-A8P1, IB-7644, IB-7645, IB-7646, IB-7647, IB-7649, IB-7651, IB-7652, IB-7654, IB-7885, IB-7886, IB-7887, IB-7888, IB-7889, IB-7890, IB-7891, IB-7893, IB-7897, IB-8126, IB-8127, IB-A5SO, IB-A5SP, IB-A5SQ, IB-A5SS, IB-A5ST, IB-A6UF, IB-A6UG, IB-A7LX, IB-A7M4, IB-AAUM, IB-AAUN, IB-AAUO, IB-AAUP, IB-AAUR, IB-AAUS, IB-AAUT, IB-AAUU, IB-AAUV, IB-AAUW, LB-A7SX, LB-A8F3, LB-A9Q5, M8-A5N4, OE-A75W, PZ-A5RE, Q3-A5QY, Q3-AA2A, RB-A7B8, RB-AA9M, RL-AAAS, S4-A8RM, S4-A8RO, S4-A8RP, US-A774, US-A776, US-A779, US-A77E, US-A77G, US-A77J, XD-AAUL, XN-A8T3, XN-A8T5, YB-A89D, YH-A8SY, YY-A8LH.
⧫ **Pheochromocytoma and Paraganglioma** (178 samples):
• Source T18 = TCGA (see Acknowledgments (main text)). Sample IDs are of the form TCGA-*, where * is:
  P7-A5NX, P7-A5NY, P8-A5KC, P8-A5KD, P8-A6RX, P8-A6RY, PR-A5PF, PR-A5PG, PR-A5PH, QR-A6GO, QR-A6GR, QR-A6GS, QR-A6GT, QR-A6GU, QR-A6GW, QR-A6GX, QR-A6GY, QR-A6GZ, QR-A6H0, QR-A6H1, QR-A6H2, QR-A6H3, QR-A6H4, QR-A6H5, QR-A6H6, QR-A6ZZ, QR-A702, QR-A703, QR-A705, QR-A706, QR-A707, QR-A708, QR-A70A, QR-A70C, QR-A70D, QR-A70E, QR-A70G, QR-A70H, QR-A70I, QR-A70J, QR-A70K, QR-A70M, QR-A70N, QR-A70O, QR-A70P, QR-A70Q, QR-A70R, QR-A70T, QR-A70U, QR-A70V, QR-A70W, QR-A70X, QR-A7IN, QR-A7IP, QT-A5XJ, QT-A5XK, QT-A5XL, QT-A5XM, QT-A5XN, QT-A5XO, QT-A5XP, QT-A69Q, QT-A7U0, RM-A68T, RM-A68W, RT-A6Y9, RT-A6YA, RT-A6YC, RW-A67V, RW-A67W, RW-A67X, RW-A67Y, RW-A680, RW-A681, RW-A684, RW-A685, RW-A686, RW-A688, RW-A689, RW-A68A, RW-A68B, RW-A68C, RW-A68D, RW-A68F, RW-A68G, RW-A7CZ, RW-A7D0, RW-A8AZ, RX-A8JQ, S7-A7WL, S7-A7WM, S7-A7WN, S7-A7WO, S7-A7WP, S7-A7WQ, S7-A7WR, S7-A7WT, S7-A7WU, S7-A7WV, S7-A7WW, S7-A7WX, S7-A7X0, S7-A7X1, S7-A7X2, SA-A6C2, SP-A6QC, SP-A6QD, SP-A6QF, SP-A6QG, SP-A6QH, SP-A6QI, SP-A6QJ, SP-A6QK, SQ-A6I4, SQ-A6I6, SR-A6MP, SR-A6MQ, SR-A6MR, SR-A6MS, SR-A6MT, SR-A6MU, SR-A6MV, SR-A6MX, SR-A6MY, SR-A6MZ, SR-A6N0, TT-A6YJ, TT-A6YK, TT-A6YN, TT-A6YO, TT-A6YP, W2-A7H5, W2-A7H7, W2-A7HA, W2-A7HB, W2-A7HC, W2-A7HD, W2-A7HE, W2-A7HF, W2-A7HH, W2-A7UY, WB-A80K, WB-A80L, WB-A80M, WB-A80N, WB-A80O, WB-A80P, WB-A80Q, WB-A80V, WB-A80Y, WB-A814, WB-A815, WB-A816, WB-A817, WB-A818, WB-A819, WB-A81A, WB-A81D, WB-A81E, WB-A81F, WB-A81G, WB-A81H, WB-A81I, WB-A81J, WB-A81K, WB-A81M, WB-A81N, WB-A81P, WB-A81Q, WB-A81R, WB-A81S, WB-A81T, WB-A81V, WB-A81W, WB-A820, WB-A821, WB-A822, XG-A823.
⧫ **Prostate Cancer** (480 samples):
• Source B2 = [80]. Sample IDs are of the form P0*-Tumor, where * is:
  0-000450, 1-28, 2-1562, 2-2035, 3-1334, 3-1426, 3-1906, 3-2345, 3-2620, 3-3391, 3-595, 3-871, 4-1084, 4-1243, 4-1421, 4-1790, 4-2599, 4-2641, 4-2666, 4-2740, 4-47, 4-594, 5-2212, 5-2594, 5-3436, 5-3829, 5-3852, 5-3859, 5-620, 6-1125, 6-1696, 6-2325, 6-3676, 6-3939, 6-4428, 7-144, 7-360, 7-5036, 7-684, 7-718, 7-837, 8-2516, 8-590, 9-120, 9-1372, 9-1580, 9-2497, 9-649.
• Source B3 = [81]. Sample IDs are of the form PR-*, where * is:
  0508, 0581, 1701, 1783, 2832, 3027, 3043.
  Remaining sample IDs are of the form PR-*-Tumor, where * is:
  00-1165, 00-160, 00-1823, 0099, 01-1934, 01-2382, 01-2492, 01-2554, 02-1082, 02-169, 02-1736, 02-1899, 02-2072, 02-2480, 02-254, 03-022, 03-1026, 03-870, 04-1367, 04-194, 04-3113, 04-3222, 04-3347, 04-639, 04-903, 0415, 0427, 05-3440, 05-3595, 05-839, 06-1651, 06-1749, 06-1999, 09-2517, 09-2744, 09-2767, 09-3421, 09-3566, 09-3687, 09-5094, 09-5245, 09-5446, 09-5630, 09-5700, 09-5702, 1024, 1043, 2661, 2682, 2740, 2761, 2762, 2858, 2872, 2915, 2916, 3023, 3026, 3034, 3035, 3036, 3048, 3051, 3127.
• Source G2 = [82] (below * stands for WA, e.g., *10 = WA10):
  T12, T32, T8, T90, T91, T92, T93, T94, T95, T96, T97, *10, *11, *12, *13, *14, *15, *16, *17, *18, *19, *20, *22, *23, *24, *25, *26, *27, *28, *29, *3, *30, *31, *32, *33, *35, *37, *38, *39, *40, *41, *42, *43-27, *43-44, *43-71, *46, *47, *48, *49, *50, *51, *52, *53, *54, *55, *56, *57, *58, *59, *60, *7.
• Source T19 = TCGA (see Acknowledgments (main text)). Sample IDs are of the form TCGA-*, where * is:
  CH-5737, CH-5738, CH-5739, CH-5740, CH-5741, CH-5743, CH-5744, CH-5745, CH-5746, CH-5748, CH-5750, CH-5751, CH-5752, CH-5753, CH-5754, CH-5761, CH-5762, CH-5763, CH-5764, CH-5765, CH-5766, CH-5767, CH-5768, CH-5769, CH-5771, CH-5772, CH-5788, CH-5789, CH-5790, CH-5791, CH-5792, CH-5794, EJ-5494, EJ-5495, EJ-5496, EJ-5497, EJ-5498, EJ-5499, EJ-5501, EJ-5502, EJ-5503, EJ-5504, EJ-5505, EJ-5506, EJ-5507, EJ-5508, EJ-5509, EJ-5510, EJ-5511, EJ-5512, EJ-5514, EJ-5515, EJ-5516, EJ-5517, EJ-5518, EJ-5519, EJ-5521, EJ-5522, EJ-5524, EJ-5525, EJ-5526, EJ-5527, EJ-5530, EJ-5531, EJ-5532, EJ-5542, EJ-7115, EJ-7123, EJ-7125, EJ-7218, EJ-7312, EJ-7314, EJ-7315, EJ-7317, EJ-7318, EJ-7321, EJ-7325, EJ-7327, EJ-7328, EJ-7330, EJ-7331, EJ-7781, EJ-7782, EJ-7783, EJ-7784, EJ-7785, EJ-7786, EJ-7788, EJ-7789, EJ-7791, EJ-7792, EJ-7793, EJ-7794, EJ-7797, EJ-8468, EJ-8469, EJ-8470, EJ-8472, EJ-8474, EJ-A46B, EJ-A46D, EJ-A46E, EJ-A46F, EJ-A46G, EJ-A46H, EJ-A46I, EJ-A65B, EJ-A65D, EJ-A65E, EJ-A65F, EJ-A65G, EJ-A65J, EJ-A65M, EJ-A6RA, EJ-A6RC, EJ-A7NF, EJ-A7NG, EJ-A7NH, EJ-A7NM, EJ-A7NN, FC-7708, FC-7961, FC-A4JI, FC-A5OB, FC-A66V, FC-A6HD, G9-6329, G9-6332, G9-6333, G9-6336, G9-6338, G9-6339, G9-6342, G9-6343, G9-6347, G9-6348, G9-6351, G9-6353, G9-6354, G9-6356, G9-6361, G9-6362, G9-6363, G9-6364, G9-6365, G9-6366, G9-6367, G9-6369, G9-6370, G9-6371, G9-6373, G9-6377, G9-6378, G9-6379, G9-6384, G9-6385, G9-6494, G9-6496, G9-6498, G9-6499, G9-7510, G9-7519, G9-7521, G9-7522, G9-7523, G9-7525, H9-7775, H9-A6BX, H9-A6BY, HC-7075, HC-7077, HC-7078, HC-7079, HC-7080, HC-7081, HC-7209, HC-7210, HC-7211, HC-7212, HC-7213, HC-7230, HC-7231, HC-7232, HC-7233, HC-7736, HC-7737, HC-7738, HC-7740, HC-7742, HC-7744, HC-7745, HC-7747, HC-7748, HC-7749, HC-7750, HC-7752, HC-7817, HC-7818, HC-7819, HC-7820, HC-7821, HC-8213, HC-8216, HC-8256, HC-8257, HC-8258, HC-8259, HC-8260, HC-8261, HC-8262, HC-8264, HC-8265, HC-8266, HC-A48F, HC-A4ZV, HC-A631, HC-A632, HC-A6AL, HC-A6AN, HC-A6AO, HC-A6AP, HC-A6AQ, HC-A6AS, HC-A6HX, HC-A6HY, HC-A76W, HC-A76X, HI-7168, HI-7169, HI-7170, HI-7171, J4-8198, J4-8200, J4-A67K, J4-A67L, J4-A67M, J4-A67N, J4-A67O, J4-A67Q, J4-A67R, J4-A67S, J4-A67T, J4-A6G1, J4-A6G3, J4-A6M7, J9-A52B, J9-A52C, J9-A52D, J9-A52E, KC-A4BL, KC-A4BN, KC-A4BO, KC-A4BR, KC-A4BV, KC-A7F3, KC-A7F5, KC-A7F6, KC-A7FA, KC-A7FD, KC-A7FE, KK-A59V, KK-A59X, KK-A59Y, KK-A59Z, KK-A5A1, KK-A6DY, KK-A6E0, KK-A6E1, KK-A6E2, KK-A6E3, KK-A6E4, KK-A6E5, KK-A6E6, KK-A6E7, KK-A6E8, KK-A7AP, KK-A7AQ, KK-A7AU, KK-A7AV, KK-A7AW, KK-A7AY, KK-A7AZ, KK-A7B0, KK-A7B1, KK-A7B2, KK-A7B3, KK-A7B4, M7-A71Y, M7-A71Z, M7-A720, M7-A721, M7-A723, M7-A724, M7-A725, QU-A6IL, QU-A6IM, QU-A6IN, QU-A6IO, QU-A6IP, SU-A7E7.
⧫ **Rectum Adenocarcinoma** (115 samples):
• Source T20 = TCGA (see Acknowledgments (main text)). Sample IDs are of the form TCGA-*, where * is:
  AF-2687, AF-2689, AF-2690, AF-2691, AF-2692, AF-2693, AF-3400, AF-3911, AF-4110, AF-5654, AF-6136, AF-6655, AF-6672, AG-3574, AG-3575, AG-3578, AG-3580, AG-3581, AG-3582, AG-3583, AG-3584, AG-3586, AG-3587, AG-3591, AG-3592, AG-3593, AG-3594, AG-3598, AG-3599, AG-3600, AG-3601, AG-3602, AG-3605, AG-3608, AG-3609, AG-3611, AG-3612, AG-3725, AG-3731, AG-3732, AG-3742, AG-4021, AG-4022, AG-A002, AG-A008, AG-A00C, AG-A00Y, AG-A011, AG-A014, AG-A015, AG-A016, AG-A01L, AH-6544, AH-6643, AH-6644, AH-6897, AH-6903, BM-6198, CI-6619, CI-6620, CI-6621, CI-6622, CI-6624, CL-4957, CL-5917, CL-5918, DC-4749, DC-5337, DC-5869, DC-6154, DC-6155, DC-6157, DC-6158, DC-6681, DC-6682, DC-6683, DT-5265, DY-A0XA, DY-A1DC, DY-A1DD, DY-A1DF, DY-A1DG, DY-A1H8, EF-5830, EI-6506, EI-6507, EI-6508, EI-6509, EI-6510, EI-6511, EI-6512, EI-6513, EI-6514, EI-6881, EI-6882, EI-6883, EI-6884, EI-6885, EI-6917, EI-7002, EI-7004, F5-6464, F5-6465, F5-6571, F5-6702, F5-6812, F5-6813, F5-6814, F5-6861, F5-6863, F5-6864, G5-6233, G5-6235, G5-6572, G5-6641.
⧫ **Renal Cell Carcinoma** (709 samples):
• Source G3 = [83]:
  K1, K20, K27, K29, K3, K31, K32, K38, K44, K48, T127, T142, T144, T163, T164, T166, T183M.
• Source T21 = TCGA (see Acknowledgments (main text)). Sample IDs are of the form TCGA-*, where * is:
  A3-3308, A3-3311, A3-3313, A3-3316, A3-3317, A3-3319, A3-3320, A3-3322, A3-3323, A3-3324, A3-3326, A3-3331, A3-3346, A3-3347, A3-3349, A3-3351, A3-3357, A3-3358, A3-3362, A3-3363, A3-3365, A3-3367, A3-3370, A3-3372, A3-3373, A3-3374, A3-3376, A3-3378, A3-3380, A3-3382, A3-3383, A3-3385, A3-3387, A4-7286, A4-7287, A4-7288, A4-7583, A4-7584, A4-7585, A4-7732, A4-7734, A4-7828, A4-7915, A4-7996, A4-7997, A4-8098, A4-8310, A4-8311, A4-8312, A4-8515, A4-8516, A4-8517, A4-8518, A4-8630, A4-A48D, A4-A4ZT, A4-A57E, A4-A5DU, A4-A5XZ, A4-A5Y0, A4-A5Y1, A4-A6HP, AK-3425, AK-3427, AK-3428, AK-3429, AK-3430, AK-3431, AK-3434, AK-3436, AK-3440, AK-3443, AK-3444, AK-3445, AK-3447, AK-3450, AK-3451, AK-3453, AK-3454, AK-3455, AK-3456, AK-3458, AK-3460, AK-3461, AK-3465, AL-3466, AL-3467, AL-3468, AL-3472, AL-3473, AL-7173, AL-A5DJ, AS-3777, AS-3778, AT-A5NU, B0-4690, B0-4691, B0-4693, B0-4694, B0-4697, B0-4700, B0-4703, B0-4706, B0-4707, B0-4710, B0-4712, B0-4713, B0-4714, B0-4718, B0-4810, B0-4811, B0-4813, B0-4814, B0-4815, B0-4816, B0-4817, B0-4818, B0-4819, B0-4822, B0-4823, B0-4824, B0-4827, B0-4828, B0-4833, B0-4836, B0-4837, B0-4838, B0-4839, B0-4841, B0-4842, B0-4843, B0-4844, B0-4845, B0-4846, B0-4847, B0-4848, B0-4849, B0-4852, B0-4945, B0-5075, B0-5077, B0-5080, B0-5081, B0-5083, B0-5084, B0-5085, B0-5088, B0-5092, B0-5094, B0-5095, B0-5096, B0-5097, B0-5098, B0-5099, B0-5100, B0-5102, B0-5104, B0-5106, B0-5107, B0-5108, B0-5109, B0-5110, B0-5113, B0-5115, B0-5116, B0-5117, B0-5119, B0-5120, B0-5121, B0-5399, B0-5400, B0-5402, B0-5691, B0-5692, B0-5693, B0-5694, B0-5695, B0-5696, B0-5697, B0-5698, B0-5699, B0-5701, B0-5702, B0-5703, B0-5705, B0-5706, B0-5707, B0-5709, B0-5710, B0-5711, B0-5712, B0-5713, B0-5812, B1-5398, B1-A47M, B1-A47N, B1-A47O, B1-A654, B1-A655, B1-A656, B1-A657, B2-3923, B2-3924, B2-4098, B2-4099, B2-4101, B2-4102, B2-5633, B2-5635, B2-5641, B3-3925, B3-3926, B3-4103, B3-4104, B3-8121, B4-5377, B4-5832, B4-5834, B4-5835, B4-5836, B4-5838, B4-5843, B4-5844, B8-4143, B8-4146, B8-4148, B8-4151, B8-4153, B8-4154, B8-4619, B8-4620, B8-4621, B8-4622, B8-5158, B8-5159, B8-5162, B8-5163, B8-5164, B8-5165, B8-5545, B8-5546, B8-5549, B8-5550, B8-5551, B8-5552, B8-5553, B9-4113, B9-4114, B9-4115, B9-4116, B9-4117, B9-4617, B9-5155, B9-5156, B9-7268, B9-A44B, B9-A5W7, B9-A5W8, B9-A5W9, B9-A69E, BP-4158, BP-4159, BP-4160, BP-4161, BP-4162, BP-4163, BP-4164, BP-4165, BP-4166, BP-4167, BP-4169, BP-4170, BP-4173, BP-4174, BP-4176, BP-4177, BP-4326, BP-4329, BP-4330, BP-4331, BP-4337, BP-4338, BP-4340, BP-4341, BP-4342, BP-4343, BP-4345, BP-4346, BP-4347, BP-4349, BP-4351, BP-4352, BP-4354, BP-4355, BP-4756, BP-4758, BP-4759, BP-4760, BP-4761, BP-4762, BP-4763, BP-4765, BP-4766, BP-4768, BP-4770, BP-4771, BP-4774, BP-4775, BP-4777, BP-4781, BP-4782, BP-4787, BP-4789, BP-4790, BP-4795, BP-4797, BP-4798, BP-4799, BP-4801, BP-4803, BP-4804, BP-4807, BP-4960, BP-4961, BP-4962, BP-4963, BP-4964, BP-4965, BP-4967, BP-4968, BP-4969, BP-4970, BP-4971, BP-4972, BP-4973, BP-4974, BP-4975, BP-4976, BP-4977, BP-4981, BP-4982, BP-4983, BP-4985, BP-4986, BP-4987, BP-4988, BP-4989, BP-4991, BP-4992, BP-4993, BP-4994, BP-4995, BP-4998, BP-4999, BP-5000, BP-5001, BP-5004, BP-5006, BP-5007, BP-5008, BP-5009, BP-5010, BP-5168, BP-5169, BP-5170, BP-5173, BP-5174, BP-5175, BP-5176, BP-5177, BP-5178, BP-5180, BP-5181, BP-5182, BP-5183, BP-5184, BP-5185, BP-5186, BP-5187, BP-5189, BP-5190, BP-5191, BP-5192, BP-5194, BP-5195, BP-5196, BP-5198, BP-5199, BP-5200, BP-5201, BP-5202, BQ-5875, BQ-5876, BQ-5877, BQ-5878, BQ-5879, BQ-5880, BQ-5881, BQ-5882, BQ-5883, BQ-5884, BQ-5885, BQ-5886, BQ-5887, BQ-5888, BQ-5889, BQ-5890, BQ-5891, BQ-5892, BQ-5893, BQ-5894, BQ-7044, BQ-7045, BQ-7046, BQ-7048, BQ-7049, BQ-7050, BQ-7051, BQ-7053, BQ-7055, BQ-7056, BQ-7058, BQ-7059, BQ-7060, BQ-7061, BQ-7062, CJ-4634, CJ-4635, CJ-4636, CJ-4637, CJ-4638, CJ-4639, CJ-4640, CJ-4641, CJ-4643, CJ-4644, CJ-4868, CJ-4869, CJ-4870, CJ-4871, CJ-4872, CJ-4873, CJ-4874, CJ-4875, CJ-4876, CJ-4878, CJ-4881, CJ-4882, CJ-4884, CJ-4885, CJ-4886, CJ-4887, CJ-4888, CJ-4889, CJ-4890, CJ-4891, CJ-4892, CJ-4893, CJ-4894, CJ-4895, CJ-4897, CJ-4899, CJ-4900, CJ-4901, CJ-4902, CJ-4903, CJ-4904, CJ-4905, CJ-4907, CJ-4908, CJ-4912, CJ-4913, CJ-4916, CJ-4918, CJ-4920, CJ-4923, CJ-5671, CJ-5672, CJ-5675, CJ-5676, CJ-5677, CJ-5678, CJ-5679, CJ-5680, CJ-5681, CJ-5682, CJ-5683, CJ-5684, CJ-5686, CJ-6027, CJ-6028, CJ-6030, CJ-6031, CJ-6032, CJ-6033, CW-5580, CW-5581, CW-5583, CW-5584, CW-5585, CW-5588, CW-5589, CW-5591, CW-6087, CW-6090, CW-6093, CW-6097, CZ-4853, CZ-4854, CZ-4856, CZ-4857, CZ-4858, CZ-4859, CZ-4861, CZ-4862, CZ-4863, CZ-4865, CZ-4866, CZ-5451, CZ-5452, CZ-5453, CZ-5454, CZ-5455, CZ-5456, CZ-5457, CZ-5458, CZ-5459, CZ-5460, CZ-5461, CZ-5462, CZ-5463, CZ-5464, CZ-5465, CZ-5466, CZ-5467, CZ-5468, CZ-5469, CZ-5470, CZ-5982, CZ-5984, CZ-5985, CZ-5986, CZ-5987, CZ-5988, CZ-5989, DV-5565, DV-5566, DV-5568, DV-5569, DV-5574, DV-5575, DV-5576, DW-5560, DW-5561, DW-7834, DW-7837, DW-7838, DW-7839, DW-7840, DW-7841, DW-7842, DW-7963, DZ-6131, DZ-6132, DZ-6133, DZ-6134, DZ-6135, EU-5904, EU-5905, EU-5906, EU-5907, EV-5901, EV-5902, EV-5903, F9-A4JJ, G7-6789, G7-6790, G7-6792, G7-6793, G7-6795, G7-6796, G7-6797, G7-7501, G7-7502, G7-A4TM, GL-6846, GL-7773, GL-7966, GL-8500, GL-A4EM, GL-A59R, GL-A59T, HE-7128, HE-7129, HE-7130, HE-A5NF, HE-A5NH, HE-A5NI, HE-A5NJ, HE-A5NK, HE-A5NL, IA-A40U, IA-A40X, IA-A40Y, IZ-8195, IZ-8196, IZ-A6M8, IZ-A6M9, J7-6720, J7-8537, KL-8323, KL-8324, KL-8325, KL-8326, KL-8327, KL-8328, KL-8329, KL-8330, KL-8331, KL-8332, KL-8333, KL-8334, KL-8335, KL-8336, KL-8337, KL-8338, KL-8339, KL-8340, KL-8341, KL-8342, KL-8343, KL-8344, KL-8345, KL-8346, KM-8438, KM-8439, KM-8440, KM-8441, KM-8442, KM-8443, KM-8476, KM-8477, KM-8639, KN-8418, KN-8419, KN-8421, KN-8422, KN-8423, KN-8424, KN-8425, KN-8426, KN-8427, KN-8428, KN-8429, KN-8430, KN-8431, KN-8432, KN-8433, KN-8434, KN-8435, KN-8436, KN-8437, KO-8403, KO-8404, KO-8405, KO-8406, KO-8407, KO-8408, KO-8409, KO-8410, KO-8411, KO-8413, KO-8414, KO-8415, KO-8416, KO-8417, KV-A6GD, KV-A6GE, MH-A55W, MH-A55Z, MH-A560, MH-A561, MH-A562, P4-A5E6, P4-A5E7, P4-A5E8, P4-A5EA, P4-A5EB, P4-A5ED, PJ-A5Z8, PJ-A5Z9, Q2-A5QZ.
⧫ **Sarcoma** (255 samples):
• Source T22 = TCGA (see Acknowledgments (main text)). Sample IDs are of the form TCGA-*, where * is:
  3B-A9HI, 3B-A9HJ, 3B-A9HL, 3B-A9HO, 3B-A9HP, 3B-A9HQ, 3B-A9HR, 3B-A9HS, 3B-A9HT, 3B-A9HU, 3B-A9HV, 3B-A9HX, 3B-A9HY, 3B-A9HZ, 3B-A9I0, 3B-A9I1, 3B-A9I3, 3R-A8YX, DX-A1KU, DX-A1KW, DX-A1KX, DX-A1KY, DX-A1KZ, DX-A1L0, DX-A1L1, DX-A1L2, DX-A1L3, DX-A1L4, DX-A23R, DX-A23T, DX-A23U, DX-A23V, DX-A23Y, DX-A240, DX-A2IZ, DX-A2J0, DX-A2J1, DX-A2J4, DX-A3LS, DX-A3LT, DX-A3LU, DX-A3LW, DX-A3LY, DX-A3M1, DX-A3M2, DX-A3U5, DX-A3U6, DX-A3U7, DX-A3U8, DX-A3U9, DX-A3UA, DX-A3UB, DX-A3UC, DX-A3UD, DX-A3UE, DX-A3UF, DX-A48J, DX-A48K, DX-A48L, DX-A48N, DX-A48O, DX-A48P, DX-A48R, DX-A48U, DX-A48V, DX-A6B7, DX-A6B8, DX-A6B9, DX-A6BA, DX-A6BB, DX-A6BE, DX-A6BF, DX-A6BG, DX-A6BH, DX-A6BK, DX-A6YQ, DX-A6YR, DX-A6YS, DX-A6YT, DX-A6YU, DX-A6YV, DX-A6YX, DX-A6YZ, DX-A6Z0, DX-A6Z2, DX-A7EF, DX-A7EI, DX-A7EL, DX-A7EM, DX-A7EN, DX-A7EO, DX-A7EQ, DX-A7ER, DX-A7ES, DX-A7ET, DX-A7EU, DX-A8BG, DX-A8BH, DX-A8BJ, DX-A8BK, DX-A8BL, DX-A8BM, DX-A8BN, DX-A8BO, DX-A8BP, DX-A8BR, DX-A8BT, DX-A8BU, DX-A8BV, DX-A8BX, DX-A8BZ, DX-AATS, DX-AB2E, DX-AB2F, DX-AB2G, DX-AB2H, DX-AB2J, DX-AB2L, DX-AB2O, DX-AB2P, DX-AB2Q, DX-AB2S, DX-AB2T, DX-AB2V, DX-AB2W, DX-AB2X, DX-AB2Z, DX-AB30, DX-AB32, DX-AB35, DX-AB36, DX-AB37, DX-AB3A, DX-AB3B, DX-AB3C, FX-A2QS, FX-A3NJ, FX-A3NK, FX-A3RE, FX-A3TO, FX-A48G, FX-A76Y, FX-A8OO, HB-A2OT, HB-A3L4, HB-A3YV, HB-A43Z, HB-A5W3, HS-A5N7, HS-A5N8, HS-A5N9, IE-A3OV, IE-A4EH, IE-A4EI, IE-A4EJ, IE-A4EK, IE-A6BZ, IF-A4AJ, IF-A4AK, IS-A3K6, IS-A3K7, IS-A3K8, IS-A3KA, IW-A3M4, IW-A3M5, IW-A3M6, JV-A5VE, JV-A5VF, JV-A75J, K1-A3PN, K1-A3PO, K1-A42W, K1-A42X, K1-A6RT, K1-A6RU, K1-A6RV, KD-A5QS, KD-A5QT, KD-A5QU, KF-A41W, LI-A67I, LI-A9QH, MB-A5Y8, MB-A5Y9, MB-A5YA, MB-A8JK, MB-A8JL, MJ-A68H, MJ-A68J, MJ-A850, MO-A47P, MO-A47R, N1-A6IA, PC-A5DK, PC-A5DL, PC-A5DM, PC-A5DN, PC-A5DO, PC-A5DP, QC-A6FX, QC-A7B5, QC-AA9N, QQ-A5V2, QQ-A5V9, QQ-A5VA, QQ-A5VB, QQ-A5VC, QQ-A5VD, QQ-A8VB, QQ-A8VD, QQ-A8VF, QQ-A8VG, QQ-A8VH, RN-A68Q, RN-AAAQ, SG-A6Z4, SG-A6Z7, SG-A849, SI-A71O, SI-A71P, SI-A71Q, SI-AA8B, SI-AA8C, UE-A6QT, UE-A6QU, VT-A80G, VT-A80J, VT-AB3D, WK-A8XO, WK-A8XQ, WK-A8XS, WK-A8XT, WK-A8XX, WK-A8XY, WK-A8XZ, WK-A8Y0, WP-A9GB, X2-A95T, X6-A7W8, X6-A7WA, X6-A7WB, X6-A7WC, X6-A7WD, X6-A8C2, X6-A8C3, X6-A8C4, X6-A8C5, X6-A8C6, X6-A8C7, X9-A971, X9-A973, Z4-A8JB, Z4-A9VC, Z4-AAPF, Z4-AAPG.
⧫ **Testicular Germ Cell Tumors** (150 samples):
• Source T23 = TCGA (see Acknowledgments (main text)). Sample IDs are of the form TCGA-*, where * is:
  2G-AAEW, 2G-AAEX, 2G-AAF1, 2G-AAF4, 2G-AAF6, 2G-AAF8, 2G-AAFE, 2G-AAFG, 2G-AAFH, 2G-AAFI, 2G-AAFJ, 2G-AAFL, 2G-AAFM, 2G-AAFN, 2G-AAFO, 2G-AAFV, 2G-AAFY, 2G-AAFZ, 2G-AAG0, 2G-AAG3, 2G-AAG5, 2G-AAG6, 2G-AAG7, 2G-AAG8, 2G-AAG9, 2G-AAGA, 2G-AAGC, 2G-AAGE, 2G-AAGF, 2G-AAGG, 2G-AAGI, 2G-AAGJ, 2G-AAGK, 2G-AAGM, 2G-AAGN, 2G-AAGO, 2G-AAGP, 2G-AAGS, 2G-AAGT, 2G-AAGV, 2G-AAGW, 2G-AAGX, 2G-AAGY, 2G-AAGZ, 2G-AAH0, 2G-AAH2, 2G-AAH3, 2G-AAH4, 2G-AAH8, 2G-AAHA, 2G-AAHC, 2G-AAHG, 2G-AAHL, 2G-AAHN, 2G-AAHP, 2G-AAHT, 2G-AAKD, 2G-AAKG, 2G-AAKH, 2G-AAKL, 2G-AAKM, 2G-AAKO, 2G-AAL5, 2G-AAL7, 2G-AALF, 2G-AALG, 2G-AALN, 2G-AALO, 2G-AALP, 2G-AALQ, 2G-AALR, 2G-AALS, 2G-AALT, 2G-AALW, 2G-AALX, 2G-AALY, 2G-AALZ, 2G-AAM2, 2G-AAM3, 2G-AAM4, 2X-A9D5, 2X-A9D6, 4K-AA1G, 4K-AA1H, 4K-AA1I, 4K-AAAL, S6-A8JW, S6-A8JX, S6-A8JY, SB-A6J6, SB-A76C, SN-A6IS, SN-A84W, SN-A84X, SN-A84Y, SO-A8JP, VF-A8A8, VF-A8A9, VF-A8AA, VF-A8AB, VF-A8AC, VF-A8AD, VF-A8AE, W4-A7U2, W4-A7U3, W4-A7U4, WZ-A7V3, WZ-A7V4, WZ-A7V5, WZ-A8D5, X3-A8G4, XE-A8H1, XE-A8H4, XE-A8H5, XE-A9SE, XE-AANI, XE-AANJ, XE-AANR, XE-AANV, XE-AAO3, XE-AAO4, XE-AAO6, XE-AAOB, XE-AAOC, XE-AAOD, XE-AAOF, XE-AAOJ, XE-AAOL, XY-A89B, XY-A8S2, XY-A8S3, XY-A9T9, YU-A90P, YU-A90Q, YU-A90S, YU-A90W, YU-A90Y, YU-A912, YU-A94D, YU-A94I, YU-AA4L, YU-AA61, ZM-AA05, ZM-AA06, ZM-AA0B, ZM-AA0D, ZM-AA0E, ZM-AA0F, ZM-AA0H, ZM-AA0N.
⧫ **Thymoma** (123 samples):
• Source T24 = TCGA (see Acknowledgments (main text)). Sample IDs are of the form TCGA-*, where * is:
  3G-AB0O, 3G-AB0Q, 3G-AB0T, 3G-AB14, 3G-AB19, 3Q-A9WF, 3S-A8YW, 3S-AAYX, 3T-AA9L, 4V-A9QI, 4V-A9QJ, 4V-A9QL, 4V-A9QM, 4V-A9QN, 4V-A9QQ, 4V-A9QR, 4V-A9QS, 4V-A9QT, 4V-A9QU, 4V-A9QW, 4V-A9QX, 4X-A9F9, 4X-A9FA, 4X-A9FB, 4X-A9FC, 4X-A9FD, 5G-A9ZZ, 5K-AAAP, 5U-AB0D, 5U-AB0E, 5U-AB0F, 5V-A9RR, X7-A8D6, X7-A8D7, X7-A8D8, X7-A8D9, X7-A8DB, X7-A8DC, X7-A8DD, X7-A8DE, X7-A8DF, X7-A8DG, X7-A8DI, X7-A8DJ, X7-A8M0, X7-A8M1, X7-A8M3, X7-A8M4, X7-A8M5, X7-A8M6, X7-A8M7, X7-A8M8, XH-A853, XM-A8R8, XM-A8R9, XM-A8RB, XM-A8RC, XM-A8RD, XM-A8RE, XM-A8RF, XM-A8RG, XM-A8RH, XM-A8RI, XM-A8RL, XM-AAZ1, XM-AAZ2, XM-AAZ3, XU-A92O, XU-A92Q, XU-A92R, XU-A92T, XU-A92U, XU-A92V, XU-A92W, XU-A92X, XU-A92Y, XU-A92Z, XU-A930, XU-A931, XU-A932, XU-A933, XU-A936, XU-AAXW, XU-AAXX, XU-AAXY, XU-AAXZ, XU-AAY0, XU-AAY1, YT-A95D, YT-A95E, YT-A95F, YT-A95G, YT-A95H, ZB-A961, ZB-A962, ZB-A963, ZB-A964, ZB-A965, ZB-A966, ZB-A969, ZB-A96A, ZB-A96B, ZB-A96C, ZB-A96D, ZB-A96E, ZB-A96F, ZB-A96G, ZB-A96H, ZB-A96I, ZB-A96K, ZB-A96L, ZB-A96M, ZB-A96O, ZB-A96P, ZB-A96Q, ZB-A96R, ZB-A96V, ZC-AAA7, ZC-AAAA, ZC-AAAF, ZC-AAAH, ZL-A9V6, ZT-A8OM.
⧫ **Thyroid Cancer** (409 samples):
• Source T25 = TCGA (see Acknowledgments (main text)). Sample IDs are of the form TCGA-*, where * is:
  BJ-A0YZ, BJ-A0Z0, BJ-A0Z2, BJ-A0Z3, BJ-A0Z5, BJ-A0Z9, BJ-A0ZA, BJ-A0ZB, BJ-A0ZC, BJ-A0ZE, BJ-A0ZF, BJ-A0ZG, BJ-A0ZH, BJ-A0ZJ, BJ-A18Y, BJ-A18Z, BJ-A190, BJ-A191, BJ-A192, BJ-A28R, BJ-A28S, BJ-A28T, BJ-A28V, BJ-A28X, BJ-A28Z, BJ-A290, BJ-A2N7, BJ-A2N8, BJ-A2N9, BJ-A2NA, BJ-A2P4, BJ-A3EZ, BJ-A3F0, BJ-A3PR, BJ-A3PT, BJ-A3PU, BJ-A45D, BJ-A45E, BJ-A45F, BJ-A45G, BJ-A45I, BJ-A45J, BJ-A45K, BJ-A4O8, BJ-A4O9, CE-A13K, CE-A27D, CE-A3MD, CE-A3ME, CE-A482, CE-A484, CE-A485, DE-A0XZ, DE-A0Y2, DE-A0Y3, DE-A2OL, DE-A3KN, DE-A4M8, DE-A4M9, DJ-A13L, DJ-A13M, DJ-A13O, DJ-A13P, DJ-A13R, DJ-A13S, DJ-A13T, DJ-A13U, DJ-A13V, DJ-A13W, DJ-A13X, DJ-A1QD, DJ-A1QE, DJ-A1QF, DJ-A1QG, DJ-A1QH, DJ-A1QI, DJ-A1QL, DJ-A1QM, DJ-A1QN, DJ-A1QO, DJ-A1QQ, DJ-A2PN, DJ-A2PO, DJ-A2PP, DJ-A2PQ, DJ-A2PR, DJ-A2PS, DJ-A2PT, DJ-A2PU, DJ-A2PV, DJ-A2PW, DJ-A2PX, DJ-A2PY, DJ-A2PZ, DJ-A2Q0, DJ-A2Q1, DJ-A2Q2, DJ-A2Q3, DJ-A2Q4, DJ-A2Q5, DJ-A2Q6, DJ-A2Q7, DJ-A2Q8, DJ-A2Q9, DJ-A2QA, DJ-A2QB, DJ-A2QC, DJ-A3UK, DJ-A3UM, DJ-A3UN, DJ-A3UO, DJ-A3UP, DJ-A3UQ, DJ-A3UR, DJ-A3US, DJ-A3UT, DJ-A3UU, DJ-A3UV, DJ-A3UW, DJ-A3UX, DJ-A3UY, DJ-A3V7, DJ-A3VA, DJ-A3VB, DJ-A3VE, DJ-A3VF, DJ-A3VJ, DJ-A3VK, DJ-A3VL, DJ-A3VM, DJ-A4UL, DJ-A4UP, DJ-A4UT, DJ-A4UW, DJ-A4V0, DJ-A4V2, DJ-A4V4, DJ-A4V5, DO-A1JZ, DO-A1K0, DO-A2HM, E3-A3DY, E3-A3DZ, E3-A3E0, E3-A3E1, E3-A3E2, E3-A3E3, E3-A3E5, E8-A242, E8-A2EA, E8-A413, E8-A415, E8-A418, E8-A419, E8-A433, E8-A436, E8-A437, E8-A44K, E8-A44M, EL-A3CL, EL-A3CM, EL-A3CN, EL-A3CO, EL-A3CP, EL-A3CR, EL-A3CS, EL-A3CT, EL-A3CU, EL-A3CV, EL-A3CW, EL-A3CX, EL-A3CY, EL-A3CZ, EL-A3D0, EL-A3D1, EL-A3D4, EL-A3D5, EL-A3D6, EL-A3GO, EL-A3GP, EL-A3GQ, EL-A3GR, EL-A3GS, EL-A3GU, EL-A3GV, EL-A3GW, EL-A3GX, EL-A3GY, EL-A3GZ, EL-A3H1, EL-A3H2, EL-A3H3, EL-A3H4, EL-A3H5, EL-A3H7, EL-A3H8, EL-A3MW, EL-A3MX, EL-A3MY, EL-A3MZ, EL-A3N2, EL-A3N3, EL-A3T0, EL-A3T1, EL-A3T2, EL-A3T3, EL-A3T6, EL-A3T7, EL-A3T8, EL-A3T9, EL-A3TA, EL-A3TB, EL-A3ZH, EL-A3ZK, EL-A3ZN, EL-A3ZQ, EL-A3ZR, EL-A3ZT, EL-A4JV, EL-A4JW, EL-A4JX, EL-A4JZ, EL-A4K0, EL-A4K2, EL-A4K4, EL-A4K6, EL-A4KD, EL-A4KG, EL-A4KH, EL-A4KI, EM-A1CS, EM-A1CT, EM-A1CU, EM-A1CV, EM-A1CW, EM-A1YA, EM-A1YB, EM-A1YC, EM-A1YD, EM-A1YE, EM-A22I, EM-A22J, EM-A22K, EM-A22L, EM-A22M, EM-A22N, EM-A22O, EM-A22P, EM-A22Q, EM-A2CJ, EM-A2CK, EM-A2CL, EM-A2CM, EM-A2CN, EM-A2CO, EM-A2CP, EM-A2CQ, EM-A2CR, EM-A2CT, EM-A2CU, EM-A2OV, EM-A2OW, EM-A2OX, EM-A2OY, EM-A2OZ, EM-A2P0, EM-A2P1, EM-A2P2, EM-A2P3, EM-A3AI, EM-A3AJ, EM-A3AK, EM-A3AL, EM-A3AN, EM-A3AO, EM-A3AP, EM-A3AQ, EM-A3AR, EM-A3FJ, EM-A3FK, EM-A3FL, EM-A3FM, EM-A3FN, EM-A3FO, EM-A3FP, EM-A3FQ, EM-A3FR, EM-A3O3, EM-A3O6, EM-A3O7, EM-A3O8, EM-A3O9, EM-A3OA, EM-A3OB, EM-A4FK, EM-A4FM, EM-A4FO, EM-A4FQ, EM-A4FR, EM-A4FV, EM-A4G1, ET-A25G, ET-A25I, ET-A25J, ET-A25K, ET-A25O, ET-A25R, ET-A2MY, ET-A2MZ, ET-A2N0, ET-A2N1, ET-A2N4, ET-A2N5, ET-A39I, ET-A39J, ET-A39K, ET-A39L, ET-A39M, ET-A39N, ET-A39O, ET-A39P, ET-A39R, ET-A39S, ET-A39T, ET-A3BN, ET-A3BO, ET-A3BP, ET-A3BQ, ET-A3BS, ET-A3BT, ET-A3BU, ET-A3BV, ET-A3BW, ET-A3BX, ET-A3DO, ET-A3DP, ET-A3DQ, ET-A3DR, ET-A3DS, ET-A3DT, ET-A3DU, ET-A3DV, ET-A3DW, ET-A40S, ET-A4KN, FE-A22Z, FE-A230, FE-A231, FE-A232, FE-A233, FE-A234, FE-A235, FE-A236, FE-A237, FE-A238, FE-A23A, FE-A3PA, FE-A3PB, FE-A3PC, FE-A3PD, FK-A3S3, FK-A3SB, FK-A3SD, FK-A3SE, FK-A3SG, FK-A3SH, FY-A2QD, FY-A3BL, FY-A3I4, FY-A3I5, FY-A3NM, FY-A3NN, FY-A3NP, FY-A3ON, FY-A3R6, FY-A3R7, FY-A3R8, FY-A3R9, FY-A3RA, FY-A3W9, FY-A3WA, FY-A40K, FY-A4B3, GE-A2C6, H2-A26U, H2-A2K9, H2-A3RH, H2-A3RI, H2-A421, IM-A3EB, IM-A3ED, IM-A3U2, IM-A3U3, J8-A3NZ, J8-A3O0, J8-A3O1, J8-A3YE, J8-A3YH, J8-A4HW, KS-A41J, KS-A4I5, KS-A4I9, KS-A4IB, L6-A4EP, L6-A4ET, L6-A4EU, MK-A4N6, MK-A4N7, MK-A4N9.
⧫ **Uterine Cancer** (305 samples):
  Source T26 = TCGA (see Acknowledgments (main text)). Sample IDs are of the form TCGA-*, where * is:
  A5-A0G3, A5-A0G5, A5-A0G9, A5-A0GA, A5-A0GB, A5-A0GD, A5-A0GE, A5-A0GH, A5-A0GI, A5-A0GJ, A5-A0GM, A5-A0GN, A5-A0GP, A5-A0GQ, A5-A0GU, A5-A0GV, A5-A0GW, A5-A0GX, A5-A0R6, A5-A0R7, A5-A0R8, A5-A0R9, A5-A0RA, A5-A0VO, A5-A0VP, A5-A0VQ, AJ-A23M, AP-A051, AP-A052, AP-A053, AP-A054, AP-A056, AP-A059, AP-A05A, AP-A05D, AP-A05H, AP-A05J, AP-A05N, AP-A05P, AP-A0L8, AP-A0L9, AP-A0LD, AP-A0LE, AP-A0LF, AP-A0LG, AP-A0LH, AP-A0LI, AP-A0LJ, AP-A0LL, AP-A0LM, AP-A0LN, AP-A0LO, AP-A0LP, AP-A0LQ, AP-A0LT, AP-A0LV, AP-A1DQ, AX-A05S, AX-A05T, AX-A05U, AX-A05W, AX-A05Y, AX-A05Z, AX-A060, AX-A062, AX-A063, AX-A064, AX-A06B, AX-A06H, AX-A06L, AX-A0IS, AX-A0IU, AX-A0IW, AX-A0J0, AX-A0J1, AX-A1C7, AX-A1C8, AX-A1CP, AX-A2H5, AX-A2HF, B5-A0JN, B5-A0JR, B5-A0JS, B5-A0JT, B5-A0JV, B5-A0JY, B5-A0JZ, B5-A0K0, B5-A0K1, B5-A0K2, B5-A0K3, B5-A0K4, B5-A0K6, B5-A0K7, B5-A0K8, B5-A0K9, B5-A11E, B5-A11F, B5-A11G, B5-A11H, B5-A11I, B5-A11J, B5-A11M, B5-A11N, B5-A11O, B5-A11Q, B5-A11R, B5-A11S, B5-A11U, B5-A11V, B5-A11W, B5-A11X, B5-A11Y, B5-A11Z, B5-A121, B5-A1MU, B5-A1MY, BG-A0LW, BG-A0LX, BG-A0M0, BG-A0M2, BG-A0M3, BG-A0M4, BG-A0M6, BG-A0M7, BG-A0M8, BG-A0M9, BG-A0MC, BG-A0MG, BG-A0MI, BG-A0MO, BG-A0MQ, BG-A0MS, BG-A0MT, BG-A0MU, BG-A0RY, BG-A0VT, BG-A0VV, BG-A0VW, BG-A0VX, BG-A0VZ, BG-A0W1, BG-A0W2, BG-A0YU, BG-A0YV, BG-A186, BG-A187, BG-A18A, BG-A18B, BG-A18C, BG-A2AE, BK-A0C9, BK-A0CA, BK-A0CB, BK-A0CC, BK-A139, BK-A13C, BS-A0T9, BS-A0TA, BS-A0TC, BS-A0TD, BS-A0TE, BS-A0TG, BS-A0TI, BS-A0TJ, BS-A0U5, BS-A0U7, BS-A0U8, BS-A0U9, BS-A0UA, BS-A0UF, BS-A0UJ, BS-A0UL, BS-A0UM, BS-A0UT, BS-A0UV, BS-A0V6, BS-A0V7, BS-A0V8, BS-A0WQ, D1-A0ZN, D1-A0ZO, D1-A0ZP, D1-A0ZQ, D1-A0ZR, D1-A0ZS, D1-A0ZU, D1-A0ZV, D1-A0ZZ, D1-A101, D1-A102, D1-A103, D1-A15V, D1-A15W, D1-A15X, D1-A15Z, D1-A160, D1-A161, D1-A163, D1-A165, D1-A167, D1-A168, D1-A169, D1-A16B, D1-A16D, D1-A16E, D1-A16F, D1-A16G, D1-A16I, D1-A16J, D1-A16N, D1-A16O, D1-A16Q, D1-A16R, D1-A16S, D1-A16X, D1-A16Y, D1-A174, D1-A176, D1-A177, D1-A17A, D1-A17B, D1-A17C, D1-A17D, D1-A17F, D1-A17H, D1-A17K, D1-A17L, D1-A17M, D1-A17N, D1-A17Q, D1-A17R, D1-A17S, D1-A17T, D1-A17U, D1-A1NU, D1-A1NX, DI-A0WH, DI-A1NN, E6-A1LZ, EO-A1Y5, EO-A1Y8, EY-A1GS, EY-A212, FI-A2D2, FI-A2EW, FI-A2EX, FI-A2F8, N5-A4R8, N5-A4RA, N5-A4RD, N5-A4RF, N5-A4RJ, N5-A4RM, N5-A4RN, N5-A4RO, N5-A4RS, N5-A4RT, N5-A4RU, N5-A4RV, N5-A59E, N5-A59F, N6-A4V9, N6-A4VC, N6-A4VD, N6-A4VE, N6-A4VF, N6-A4VG, N7-A4Y0, N7-A4Y5, N7-A4Y8, N7-A59B, N8-A4PI, N8-A4PL, N8-A4PM, N8-A4PN, N8-A4PO, N8-A4PP, N8-A4PQ, N8-A56S, N9-A4PZ, N9-A4Q1, N9-A4Q3, N9-A4Q4, N9-A4Q7, N9-A4Q8, NA-A4QV, NA-A4QW, NA-A4QX, NA-A4QY, NA-A4R0, NA-A4R1, NA-A5I1, ND-A4W6, ND-A4WA, ND-A4WC, ND-A4WF, NF-A4WU, NF-A4WX, NF-A4X2, NF-A5CP, NG-A4VU, NG-A4VW, QM-A5NM, QN-A5NN.

**Table A1:** Occurrence counts for cancer types X1–X16 for the first 48 mutation categories for the exome data summarized in Table 1 aggregated by cancer types. Here and in tables below, the mutations (abbreviated as “Mut.”) are encoded as follows: XYZW = Y > W: XYZ.

Mut.	X1	X2	X3	X4	X5	X6	X7	X8	X9	X10	X11	X12	X13	X14	X15	X16
ACAA	9	11	156	8	8	345	195	1823	190	136	9	681	513	493	178	892
ACCA	7	17	148	6	13	371	248	1775	177	112	11	726	465	561	214	712
ACGA	0	5	100	1	4	252	58	431	88	55	2	321	203	147	54	446
ACTA	6	6	105	6	6	259	436	1411	164	51	10	981	277	438	132	514
CCAA	13	13	333	5	18	520	258	1750	322	132	9	2019	823	1145	171	1426
CCCA	12	9	279	2	10	388	273	1506	222	134	6	2246	570	1681	172	1099
CCGA	3	10	180	3	9	373	127	541	175	102	7	1427	424	882	87	836
CCTA	10	14	292	2	9	403	1771	1804	298	121	11	5562	844	4206	191	1153
GCAA	12	12	278	11	15	356	127	1437	245	134	10	1258	824	816	118	1146
GCCA	7	8	270	5	13	382	218	1281	245	190	9	1265	486	772	138	842
GCGA	6	7	163	4	17	262	99	372	95	142	1	698	331	357	60	656
GCTA	18	1	224	7	20	332	571	1216	233	111	6	2222	611	1447	112	700
TCAA	16	5	273	7	12	1800	147	4102	867	143	7	3007	718	688	160	1450
TCCA	18	17	282	9	20	1247	305	2813	601	159	10	1634	709	796	180	1490
TCGA	1	5	93	2	3	549	84	640	273	73	3	821	397	268	69	632
TCTA	25	13	281	13	8	2214	1292	4889	1150	126	13	15896	830	2202	157	1359
ACAG	5	13	50	5	7	272	147	1287	138	40	9	374	239	250	121	423
ACCG	3	3	50	3	4	190	115	968	84	51	3	289	221	350	95	381
ACGG	3	8	33	2	4	169	43	516	71	37	2	139	110	143	66	229
ACTG	3	6	55	3	3	275	169	1363	88	52	6	446	268	374	129	440
CCAG	4	12	71	1	3	406	89	1186	162	63	6	272	216	270	109	517
CCCG	1	2	66	5	8	277	89	876	102	63	4	249	212	202	86	426
CCGG	0	11	48	1	8	290	40	569	93	52	2	230	140	148	55	397
CCTG	2	3	97	2	2	519	110	1594	190	73	11	335	329	341	129	636
GCAG	3	7	52	8	3	256	94	720	85	32	3	251	170	227	87	310
GCCG	3	5	83	4	4	219	136	791	97	46	3	493	220	470	92	387
GCGG	7	7	42	2	0	183	39	335	34	40	3	144	109	100	29	260
GCTG	3	1	64	9	8	273	133	1106	118	52	4	383	248	314	93	371
TCAG	5	5	151	8	6	6944	164	13309	4554	96	7	478	1155	539	179	4640
TCCG	4	5	135	5	8	2539	134	4348	1400	74	4	415	546	453	159	1864
TCGG	4	8	43	0	1	995	71	997	649	46	2	159	225	138	46	706
TCTG	8	6	216	6	6	8468	332	17536	4883	126	14	766	1446	900	250	5340
ACAT	13	43	114	10	10	663	739	2293	292	132	21	2578	665	1964	339	1017
ACCT	20	28	99	10	15	626	582	1511	235	154	15	2141	589	1775	438	1081
ACGT	77	155	650	27	26	2163	3790	4880	1264	442	61	12877	3611	15083	3208	3132
ACTT	4	20	69	4	9	519	594	1625	190	97	15	1672	505	1204	294	765
CCAT	28	28	158	15	19	1363	451	2613	597	198	12	2612	910	1442	428	2033
CCCT	24	21	154	6	18	930	651	1735	340	188	17	2422	941	1508	729	2167
CCGT	71	158	586	32	36	2541	2669	4215	1538	502	45	13132	3976	14824	1933	4047
CCTT	26	54	174	9	28	1196	582	2496	444	220	19	2210	956	1604	497	2161
GCAT	10	19	213	22	23	902	1019	2266	445	173	20	4317	784	3857	316	1392
GCCT	19	34	231	16	23	1197	1447	2181	470	232	15	7695	1094	6834	985	1864
GCGT	80	131	662	29	40	2440	4477	4580	1665	452	44	19823	4183	21712	2596	3885
GCTT	8	37	234	16	12	907	1128	2095	432	194	18	5329	888	4192	406	1269
TCAT	29	30	252	13	7	12157	353	21889	7214	226	19	2277	1942	1756	340	7315
TCCT	43	33	227	16	14	4126	741	6715	2397	204	25	3327	1302	1988	594	4506
TCGT	58	73	356	19	21	6568	1826	7512	3602	293	27	17135	2630	9312	1178	4328
TCTT	25	19	193	8	17	6716	611	12470	3850	166	18	3770	1474	1666	413	4591

**Table A2:** Table A1, continued: occurrence counts (aggregated by cancer types) for the next 48 mutation categories for cancer types X1–X16.

Mut.	X1	X2	X3	X4	X5	X6	X7	X8	X9	X10	X11	X12	X13	X14	X15	X16
ATAA	1	0	44	9	4	125	48	729	34	85	4	279	123	261	50	293
ATCA	3	8	56	10	5	162	90	861	64	70	7	564	224	791	74	407
ATGA	0	3	48	8	6	234	78	893	71	202	14	280	206	296	83	424
ATTA	8	2	21	4	3	151	55	1087	49	44	8	603	171	529	58	295
CTAA	2	2	29	5	0	132	34	525	29	192	2	242	82	131	41	209
CTCA	2	7	67	6	6	221	139	866	70	200	5	530	328	529	87	481
CTGA	8	10	128	5	11	480	132	888	93	691	14	606	310	430	154	845
CTTA	3	2	50	5	6	221	110	981	80	179	6	560	535	714	103	507
GTAA	0	2	41	5	0	129	45	412	34	119	6	315	79	173	40	232
GTCA	3	3	43	7	2	121	75	544	40	62	4	443	134	575	45	263
GTGA	2	3	42	4	4	166	44	537	44	194	10	302	117	239	60	379
GTTA	1	5	20	0	3	117	65	666	37	44	6	326	184	337	55	219
TTAA	0	1	20	3	2	105	36	905	35	75	7	281	83	187	32	199
TTCA	5	4	33	3	4	161	65	660	53	107	8	363	155	281	65	278
TTGA	4	3	30	2	5	163	48	502	31	188	5	231	99	140	53	218
TTTA	2	3	26	3	3	141	93	1054	45	67	9	459	194	261	58	258
ATAC	5	17	104	4	14	632	350	1677	138	174	17	1585	541	1300	160	1137
ATCC	11	12	63	6	4	319	349	964	110	93	15	2728	330	1306	143	450
ATGC	10	21	104	7	21	731	493	1781	188	234	14	3410	521	2601	208	876
ATTC	3	19	90	7	11	535	459	1762	153	118	18	1670	484	977	236	834
CTAC	5	6	48	4	7	267	175	808	88	100	5	1110	273	1092	117	403
CTCC	3	14	68	9	13	459	406	1316	154	125	11	4194	724	1625	252	526
CTGC	15	15	130	9	19	692	450	1464	249	245	14	4936	842	3653	324	863
CTTC	3	10	75	8	9	427	337	1148	162	142	12	3577	1673	2424	222	780
GTAC	10	4	112	8	10	426	354	1164	163	113	10	2598	434	3655	126	808
GTCC	8	8	73	14	9	373	443	780	227	90	15	4544	483	2647	169	575
GTGC	8	7	83	7	13	413	331	1004	204	143	9	3583	456	3093	140	776
GTTC	4	8	66	3	11	391	379	1134	194	124	16	3277	459	2338	179	773
TTAC	3	0	54	5	6	288	150	846	77	82	9	941	196	1012	78	353
TTCC	11	9	63	11	11	290	339	1048	165	93	6	2964	393	1362	155	428
TTGC	3	4	77	4	8	352	342	721	141	113	7	2088	283	1444	99	456
TTTC	5	15	52	7	11	267	318	1037	152	82	10	1910	282	899	142	423
ATAG	2	1	12	6	4	63	43	423	33	25	4	208	62	104	24	110
ATCG	2	2	14	6	0	93	85	320	43	26	4	446	86	258	41	115
ATGG	0	2	26	6	4	128	77	588	50	44	2	276	130	205	55	156
ATTG	1	1	19	5	2	132	136	564	112	43	2	1193	322	588	38	123
CTAG	1	1	14	2	1	74	29	318	19	36	6	238	70	162	21	77
CTCG	2	5	28	5	4	129	99	519	49	74	6	624	271	737	50	149
CTGG	3	10	60	6	9	271	93	826	87	86	8	863	339	934	95	317
CTTG	2	4	19	32	6	266	176	994	123	129	14	2194	3027	3903	59	313
GTAG	0	3	25	4	1	138	31	544	31	24	3	161	75	125	23	102
GTCG	2	2	28	2	4	159	67	464	50	19	4	362	138	334	49	93
GTGG	5	4	133	2	10	656	116	5379	58	54	2	368	155	314	118	243
GTTG	2	1	27	13	7	152	84	683	85	31	4	714	727	1161	48	109
TTAG	1	3	9	3	1	102	35	503	59	21	6	466	84	137	35	96
TTCG	2	4	29	4	2	159	86	474	98	65	1	778	179	367	30	172
TTGG	3	1	20	3	6	151	82	635	59	63	3	351	141	247	61	226
TTTG	3	5	22	8	4	415	196	1189	317	89	5	4569	555	1361	63	214

**Table A3:** Occurrence counts for cancer types X17–X32 for the first 48 mutation categories for the exome data summarized in Table 1 aggregated by cancer types.

Mut.	X17	X18	X19	X20	X21	X22	X23	X24	X25	X26	X27	X28	X29	X30	X31	X32
ACAA	2813	6149	562	63	323	308	147	36	264	104	555	273	58	64	56	457
ACCA	2218	6108	394	8	340	352	214	28	216	131	511	256	111	55	51	554
ACGA	1014	3979	157	2	193	67	68	21	64	41	202	127	67	36	34	185
ACTA	1623	4089	350	11	187	217	351	10	129	212	360	189	52	36	43	1594
CCAA	2593	13874	2172	40	379	326	291	38	259	165	673	304	109	75	68	1094
CCCA	2454	12582	851	18	312	317	257	33	189	125	645	261	95	62	42	1238
CCGA	1644	8004	1897	16	225	82	296	22	145	132	338	198	128	66	53	548
CCTA	2054	10717	1243	14	350	287	1368	17	233	469	704	323	114	83	52	6273
GCAA	2244	6380	358	30	662	260	147	28	273	162	517	959	104	44	55	586
GCCA	2318	7537	333	22	756	292	322	22	234	149	531	943	195	37	32	899
GCGA	1534	5020	153	14	375	80	124	15	135	85	261	571	146	49	30	239
GCTA	1597	4815	286	23	610	245	1352	18	159	297	482	2182	136	44	36	3015
TCAA	2141	6882	941	26	309	259	399	21	214	782	696	242	67	48	57	2984
TCCA	2380	10492	1213	16	319	373	404	30	297	262	727	248	83	45	52	1575
TCGA	926	3791	429	4	204	88	240	16	120	141	255	137	69	36	40	409
TCTA	2464	7464	864	14	256	306	1254	32	261	5224	703	278	82	40	41	22736
ACAG	1852	1947	301	8	44	276	83	23	130	55	284	111	39	15	20	168
ACCG	1075	1377	284	3	33	166	39	30	95	42	309	98	30	21	28	181
ACGG	824	901	214	16	22	110	51	10	48	21	148	70	51	16	21	140
ACTG	1621	1467	319	5	31	247	65	14	118	50	324	92	39	13	33	213
CCAG	1139	2373	735	12	51	257	83	26	109	49	279	104	24	11	38	93
CCCG	1417	2025	684	11	69	238	54	22	91	35	355	92	19	13	43	97
CCGG	857	1762	823	26	41	120	44	8	83	53	223	73	28	18	29	128
CCTG	1715	2383	496	6	88	315	82	28	158	38	506	154	51	23	45	154
GCAG	958	1595	172	31	28	176	61	18	76	50	222	84	36	13	21	122
GCCG	1091	1993	337	88	29	165	66	27	131	62	241	104	32	20	43	215
GCGG	873	1350	181	90	12	49	30	12	37	35	135	51	25	12	15	62
GCTG	1143	1498	226	31	41	293	101	19	136	55	279	124	66	23	43	201
TCAG	1485	7479	480	18	396	396	175	37	189	87	429	191	43	37	129	548
TCCG	1511	4330	676	12	217	371	108	36	173	72	496	197	37	23	82	253
TCGG	745	1559	264	13	64	82	62	13	45	33	173	52	30	12	23	118
TCTG	2700	8876	790	17	437	598	196	39	304	141	714	341	58	24	173	653
ACAT	6483	3019	839	12	91	333	446	52	321	173	667	294	85	66	109	2148
ACCT	4063	2437	9149	14	108	247	704	61	243	222	616	398	65	42	139	1921
ACGT	8931	4557	1387	58	454	659	2672	222	1955	2009	1758	1318	199	310	360	7951
ACTT	4600	1931	3358	14	54	253	609	55	240	207	545	256	76	44	93	1766
CCAT	4593	6129	23356	16	236	400	367	70	341	201	910	734	98	49	136	937
CCCT	5514	4925	35156	31	245	346	575	62	341	217	860	1061	94	52	132	1405
CCGT	9037	6230	9358	61	616	690	2479	167	1900	1747	1671	1382	194	275	368	7385
CCTT	6055	5475	26855	22	178	366	752	82	390	259	913	960	138	83	138	1599
GCAT	3913	3263	895	18	126	454	962	79	430	244	917	304	147	89	125	4051
GCCT	5014	3961	8614	38	183	380	2130	173	742	693	1161	553	197	125	242	6013
GCGT	7796	5744	1491	64	558	610	5591	312	2235	3018	1719	1415	329	410	365	12763
GCTT	4278	3066	6132	16	116	350	1770	112	453	652	890	388	151	100	175	5056
TCAT	3855	11556	34755	48	595	534	499	53	405	268	1142	800	68	56	299	1758
TCCT	4397	7323	84453	28	466	449	866	95	447	571	1017	1728	67	88	212	3178
TCGT	5045	5329	33171	40	595	456	2073	111	1022	6497	1025	1086	96	138	235	18507
TCTT	4336	7487	27330	69	326	376	932	64	355	789	752	842	58	62	155	4029

**Table A4:** Table A3, continued: occurrence counts (aggregated by cancer types) for the next 48 mutation categories for cancer types X17–X32.

Mut.	X17	X18	X19	X20	X21	X22	X23	X24	X25	X26	X27	X28	X29	X30	X31	X32
ATAA	2099	1060	431	31	9	73	52	9	76	34	390	54	9	4	17	119
ATCA	1201	1112	418	4	22	151	75	29	97	54	238	93	24	16	41	256
ATGA	1558	2176	451	6	26	148	55	19	77	40	300	108	23	14	40	119
ATTA	1501	873	1059	2	17	116	98	12	91	62	265	99	12	18	21	424
CTAA	1106	1366	219	9	16	60	33	11	43	22	914	49	5	8	9	62
CTCA	1580	2157	567	3	26	188	99	25	90	56	443	126	16	21	25	252
CTGA	3163	6266	518	2	30	191	122	29	132	100	540	140	44	31	42	212
CTTA	1253	2176	762	2	17	194	80	10	104	80	377	110	23	70	19	271
GTAA	737	1419	211	24	22	73	31	11	65	20	377	60	8	5	10	103
GTCA	808	1125	248	5	9	111	72	12	65	36	204	91	22	16	12	218
GTGA	1232	2391	395	6	31	126	38	12	72	35	243	82	15	17	255	131
GTTA	888	1029	356	3	11	102	64	12	52	38	143	67	9	3	17	185
TTAA	1735	827	424	6	17	49	23	6	71	39	264	57	18	2	8	90
TTCA	897	1062	577	1	26	145	50	7	66	38	327	96	22	8	16	217
TTGA	1148	1623	379	1	16	82	21	4	49	36	251	61	12	6	17	113
TTTA	1985	940	1538	4	12	131	47	6	67	80	329	94	11	7	20	299
ATAC	9096	2438	512	9	39	189	243	66	251	135	530	168	43	54	101	922
ATCC	2835	1074	467	24	40	183	266	43	171	125	498	137	62	40	68	1163
ATGC	8618	2982	746	10	45	248	363	93	252	122	761	213	49	56	92	1716
ATTC	5928	1716	776	10	39	261	298	84	312	165	566	213	35	57	133	788
CTAC	2951	1387	613	4	16	87	94	26	108	85	432	93	34	23	54	557
CTCC	3360	1350	880	92	32	220	290	30	202	127	727	280	180	66	96	957
CTGC	6754	3236	1008	28	64	209	414	90	284	117	910	257	89	78	126	2141
CTTC	4047	1913	1913	9	33	224	391	50	231	127	730	347	255	117	159	812
GTAC	3048	1831	513	2	44	123	390	38	229	204	422	157	38	47	56	1717
GTCC	2708	1127	591	28	39	131	445	29	237	311	404	151	54	52	35	1844
GTGC	4987	1824	626	13	48	140	451	39	204	166	515	198	45	49	53	1871
GTTC	3124	1410	3319	6	40	163	400	50	255	267	494	236	51	77	76	1833
TTAC	3220	921	934	6	19	91	106	22	138	108	272	101	25	21	24	782
TTCC	2872	962	930	22	36	176	207	32	166	163	396	180	46	43	40	1411
TTGC	3895	1271	903	7	18	114	254	54	159	110	426	143	51	36	59	1429
TTTC	3660	994	986	3	22	148	245	58	168	175	417	140	37	37	45	1159
ATAG	1333	325	200	1	6	42	39	7	52	45	122	21	13	10	4	138
ATCG	705	295	272	1	14	58	97	6	55	62	230	35	13	18	5	422
ATGG	1122	522	549	4	13	87	76	12	49	40	229	78	21	14	8	215
ATTG	1101	348	334	0	7	55	182	7	95	239	272	30	18	8	19	1241
CTAG	503	238	119	0	11	43	13	3	36	37	79	22	3	4	8	184
CTCG	1030	504	443	6	9	84	100	11	92	42	210	70	11	16	24	372
CTGG	1519	1384	606	30	22	164	128	21	112	64	291	101	15	14	33	577
CTTG	1240	1100	1090	4	29	141	280	19	154	271	336	88	15	17	31	1741
GTAG	643	319	128	61	9	44	39	6	48	24	102	24	21	9	6	78
GTCG	658	311	202	69	5	52	100	10	65	55	127	34	58	19	8	259
GTGG	1314	835	904	384	33	121	190	15	128	33	350	103	124	24	15	181
GTTG	1097	478	467	35	12	67	178	9	92	67	171	62	36	19	7	408
TTAG	960	272	237	1	10	54	31	7	48	120	117	26	9	2	5	390
TTCG	1040	462	454	0	12	93	116	9	86	148	315	82	11	52	19	754
TTGG	1331	733	419	4	14	119	62	18	82	64	267	65	29	6	18	264
TTTG	2232	677	882	2	25	118	379	12	95	1083	472	66	14	10	26	4615

**Table A5:** Top-10 clusterings (Clustering-E1–Clustering-E10) by occurrence counts (second column) in 30,000 runs (performed as 3 consecutive batches of 10,000 runs in each batch). Each run is based on 1000 samplings (i.e., num.try = 1000 in the R function qrm.stat.ind.class() in Appendix A of [16]); also, the target number of clusters is k = 13, which is based on the effective rank, also known as eRank (and is computed using the R function bio.erank.pc() in Appendix B of [13]). The columns “Cl-1”–“Cl-13” give the numbers of mutations in each cluster (the total number of mutations in each clustering is 96). The entries “–” correspond to clusterings with fewer than 13 (the target number) of clusters (note that top-10 clusterings have either 11 or 12 clusters; however, there are other, less frequently occurring clusterings with 13 clusters). While there was variability in the placement (by occurrence counts) of the top-10 clusterings within the aforesaid 3 batches of 10,000 runs, in each batch, Clustering-E1 invariably had the highest count by a large margin: Batch1, Clustering-E1 count = 95, second place count = 47; Batch2, Clustering-E1 count = 124, second place count = 46; Batch3, Clustering-E1 count = 115, second place count = 49.

Name	Count	Cl-1	Cl-2	Cl-3	Cl-4	Cl-5	Cl-6	Cl-7	Cl-8	Cl-9	Cl-10	Cl-11	Cl-12	Cl-13
Clustering-E1	334	3	4	6	6	6	7	7	9	16	16	16	–	–
Clustering-E2	134	3	4	6	6	6	7	7	7	8	9	16	17	–
Clustering-E3	126	2	4	6	6	6	7	7	9	16	16	17	–	–
Clustering-E4	120	2	4	6	6	6	7	7	8	9	9	16	16	–
Clustering-E5	112	3	4	6	6	6	7	7	9	15	16	17	–	–
Clustering-E6	109	1	3	3	6	6	6	7	7	9	16	16	16	–
Clustering-E7	109	3	3	6	6	6	7	7	8	9	10	15	16	–
Clustering-E8	105	3	4	6	6	6	7	7	7	9	10	15	16	–
Clustering-E9	78	2	4	6	6	6	6	7	7	9	11	16	16	–
Clustering-E10	76	3	3	6	6	6	7	7	8	8	9	16	17	–

**Table A6:** Weights (in the units of 1%, rounded to 2 digits) for the first 48 mutation categories for the 11 clusters in Clustering-E1 (see Table A5) based on unnormalized regressions (see Section 3.2 for details). Each cluster is defined as containing the mutations with nonzero weights. For instance, cluster Cl-1 contains 3 mutations GCGA, TCGA, CTGA (also see Table A7). In each cluster, the weights are normalized to add up to 100% (up to 2 digits due to the aforesaid rounding).

Mutation	Cl-1	Cl-2	Cl-3	Cl-4	Cl-5	Cl-6	Cl-7	Cl-8	Cl-9	Cl-10	Cl-11
ACAA	0.00	0.00	0.00	0.00	0.00	0.00	0.00	0.00	3.51	0.00	0.00
ACCA	0.00	0.00	0.00	0.00	0.00	0.00	0.00	0.00	3.45	0.00	0.00
ACGA	0.00	0.00	0.00	0.00	0.00	0.00	14.21	0.00	0.00	0.00	0.00
ACTA	0.00	0.00	0.00	0.00	0.00	0.00	0.00	0.00	2.78	0.00	0.00
CCAA	0.00	0.00	0.00	0.00	0.00	0.00	0.00	0.00	5.01	0.00	0.00
CCCA	0.00	0.00	0.00	0.00	0.00	0.00	0.00	0.00	4.19	0.00	0.00
CCGA	0.00	0.00	0.00	0.00	0.00	0.00	0.00	0.00	3.05	0.00	0.00
CCTA	0.00	0.00	0.00	0.00	0.00	0.00	0.00	0.00	6.91	0.00	0.00
GCAA	0.00	0.00	0.00	0.00	0.00	0.00	0.00	0.00	5.05	0.00	0.00
GCCA	0.00	0.00	0.00	0.00	0.00	0.00	0.00	0.00	5.39	0.00	0.00
GCGA	39.73	0.00	0.00	0.00	0.00	0.00	0.00	0.00	0.00	0.00	0.00
GCTA	0.00	0.00	0.00	0.00	0.00	0.00	0.00	0.00	6.39	0.00	0.00
TCAA	0.00	0.00	0.00	0.00	0.00	0.00	0.00	0.00	6.05	0.00	0.00
TCCA	0.00	0.00	0.00	0.00	0.00	0.00	0.00	0.00	5.20	0.00	0.00
TCGA	25.16	0.00	0.00	0.00	0.00	0.00	0.00	0.00	0.00	0.00	0.00
TCTA	0.00	0.00	0.00	0.00	0.00	0.00	0.00	0.00	15.88	0.00	0.00
ACAG	0.00	0.00	0.00	0.00	0.00	0.00	14.55	0.00	0.00	0.00	0.00
ACCG	0.00	0.00	13.07	0.00	0.00	0.00	0.00	0.00	0.00	0.00	0.00
ACGG	0.00	0.00	0.00	0.00	0.00	9.41	0.00	0.00	0.00	0.00	0.00
ACTG	0.00	0.00	15.05	0.00	0.00	0.00	0.00	0.00	0.00	0.00	0.00
CCAG	0.00	0.00	0.00	0.00	0.00	0.00	14.65	0.00	0.00	0.00	0.00
CCCG	0.00	0.00	0.00	0.00	0.00	0.00	12.89	0.00	0.00	0.00	0.00
CCGG	0.00	0.00	0.00	0.00	0.00	0.00	11.72	0.00	0.00	0.00	0.00
CCTG	0.00	0.00	0.00	0.00	0.00	0.00	17.13	0.00	0.00	0.00	0.00
GCAG	0.00	0.00	0.00	0.00	0.00	14.48	0.00	0.00	0.00	0.00	0.00
GCCG	0.00	0.00	0.00	14.07	0.00	0.00	0.00	0.00	0.00	0.00	0.00
GCGG	0.00	0.00	0.00	0.00	0.00	25.13	0.00	0.00	0.00	0.00	0.00
GCTG	0.00	0.00	0.00	7.35	0.00	0.00	0.00	0.00	0.00	0.00	0.00
TCAG	0.00	0.00	0.00	0.00	0.00	0.00	0.00	0.00	10.41	0.00	0.00
TCCG	0.00	0.00	0.00	0.00	0.00	0.00	0.00	0.00	4.51	0.00	0.00
TCGG	0.00	0.00	0.00	0.00	0.00	0.00	14.86	0.00	0.00	0.00	0.00
TCTG	0.00	0.00	0.00	0.00	0.00	0.00	0.00	0.00	12.23	0.00	0.00
ACAT	0.00	0.00	0.00	0.00	0.00	0.00	0.00	0.00	0.00	1.63	0.00
ACCT	0.00	0.00	0.00	0.00	0.00	0.00	0.00	0.00	0.00	2.62	0.00
ACGT	0.00	0.00	0.00	0.00	0.00	0.00	0.00	0.00	0.00	7.61	0.00
ACTT	0.00	0.00	0.00	0.00	0.00	0.00	0.00	0.00	0.00	0.00	6.83
CCAT	0.00	0.00	0.00	0.00	0.00	0.00	0.00	0.00	0.00	4.65	0.00
CCCT	0.00	0.00	0.00	0.00	0.00	0.00	0.00	0.00	0.00	6.18	0.00
CCGT	0.00	0.00	0.00	0.00	0.00	0.00	0.00	0.00	0.00	8.07	0.00
CCTT	0.00	0.00	0.00	0.00	0.00	0.00	0.00	0.00	0.00	5.36	0.00
GCAT	0.00	0.00	0.00	0.00	0.00	0.00	0.00	0.00	0.00	2.17	0.00
GCCT	0.00	0.00	0.00	0.00	0.00	0.00	0.00	0.00	0.00	4.48	0.00
GCGT	0.00	0.00	0.00	0.00	0.00	0.00	0.00	0.00	0.00	9.57	0.00
GCTT	0.00	0.00	0.00	0.00	0.00	0.00	0.00	0.00	0.00	3.32	0.00
TCAT	0.00	0.00	0.00	0.00	0.00	0.00	0.00	0.00	0.00	9.26	0.00
TCCT	0.00	0.00	0.00	0.00	0.00	0.00	0.00	0.00	0.00	13.86	0.00
TCGT	0.00	0.00	0.00	0.00	0.00	0.00	0.00	0.00	0.00	12.42	0.00
TCTT	0.00	0.00	0.00	0.00	0.00	0.00	0.00	0.00	0.00	7.13	0.00

**Table A7:** Table A6, continued: weights for the next 48 mutation categories.

Mutation	Cl-1	Cl-2	Cl-3	Cl-4	Cl-5	Cl-6	Cl-7	Cl-8	Cl-9	Cl-10	Cl-11
ATAA	0.00	0.00	0.00	0.00	0.00	13.50	0.00	0.00	0.00	0.00	0.00
ATCA	0.00	0.00	15.56	0.00	0.00	0.00	0.00	0.00	0.00	0.00	0.00
ATGA	0.00	0.00	16.90	0.00	0.00	0.00	0.00	0.00	0.00	0.00	0.00
ATTA	0.00	0.00	0.00	0.00	14.10	0.00	0.00	0.00	0.00	0.00	0.00
CTAA	0.00	0.00	0.00	0.00	0.00	9.12	0.00	0.00	0.00	0.00	0.00
CTCA	0.00	0.00	17.42	0.00	0.00	0.00	0.00	0.00	0.00	0.00	0.00
CTGA	35.11	0.00	0.00	0.00	0.00	0.00	0.00	0.00	0.00	0.00	0.00
CTTA	0.00	0.00	0.00	0.00	19.22	0.00	0.00	0.00	0.00	0.00	0.00
GTAA	0.00	0.00	0.00	0.00	0.00	11.14	0.00	0.00	0.00	0.00	0.00
GTCA	0.00	0.00	0.00	0.00	0.00	0.00	0.00	13.54	0.00	0.00	0.00
GTGA	0.00	0.00	22.00	0.00	0.00	0.00	0.00	0.00	0.00	0.00	0.00
GTTA	0.00	0.00	0.00	0.00	0.00	0.00	0.00	10.69	0.00	0.00	0.00
TTAA	0.00	19.89	0.00	0.00	0.00	0.00	0.00	0.00	0.00	0.00	0.00
TTCA	0.00	26.53	0.00	0.00	0.00	0.00	0.00	0.00	0.00	0.00	0.00
TTGA	0.00	24.44	0.00	0.00	0.00	0.00	0.00	0.00	0.00	0.00	0.00
TTTA	0.00	29.14	0.00	0.00	0.00	0.00	0.00	0.00	0.00	0.00	0.00
ATAC	0.00	0.00	0.00	0.00	0.00	0.00	0.00	0.00	0.00	0.00	6.02
ATCC	0.00	0.00	0.00	0.00	0.00	0.00	0.00	0.00	0.00	0.00	5.08
ATGC	0.00	0.00	0.00	0.00	0.00	0.00	0.00	0.00	0.00	0.00	7.83
ATTC	0.00	0.00	0.00	0.00	0.00	0.00	0.00	0.00	0.00	0.00	6.08
CTAC	0.00	0.00	0.00	0.00	19.38	0.00	0.00	0.00	0.00	0.00	0.00
CTCC	0.00	0.00	0.00	0.00	0.00	0.00	0.00	0.00	0.00	0.00	7.15
CTGC	0.00	0.00	0.00	0.00	0.00	0.00	0.00	0.00	0.00	1.67	0.00
CTTC	0.00	0.00	0.00	0.00	0.00	0.00	0.00	0.00	0.00	0.00	7.81
GTAC	0.00	0.00	0.00	0.00	0.00	0.00	0.00	0.00	0.00	0.00	6.00
GTCC	0.00	0.00	0.00	0.00	0.00	0.00	0.00	0.00	0.00	0.00	6.68
GTGC	0.00	0.00	0.00	0.00	0.00	0.00	0.00	0.00	0.00	0.00	6.45
GTTC	0.00	0.00	0.00	0.00	0.00	0.00	0.00	0.00	0.00	0.00	6.71
TTAC	0.00	0.00	0.00	0.00	18.54	0.00	0.00	0.00	0.00	0.00	0.00
TTCC	0.00	0.00	0.00	0.00	0.00	0.00	0.00	0.00	0.00	0.00	5.16
TTGC	0.00	0.00	0.00	0.00	0.00	0.00	0.00	0.00	0.00	0.00	4.64
TTTC	0.00	0.00	0.00	0.00	0.00	0.00	0.00	0.00	0.00	0.00	4.63
ATAG	0.00	0.00	0.00	0.00	0.00	0.00	0.00	9.17	0.00	0.00	0.00
ATCG	0.00	0.00	0.00	0.00	0.00	0.00	0.00	10.65	0.00	0.00	0.00
ATGG	0.00	0.00	0.00	0.00	0.00	0.00	0.00	11.82	0.00	0.00	0.00
ATTG	0.00	0.00	0.00	0.00	14.65	0.00	0.00	0.00	0.00	0.00	0.00
CTAG	0.00	0.00	0.00	0.00	0.00	0.00	0.00	6.72	0.00	0.00	0.00
CTCG	0.00	0.00	0.00	0.00	0.00	0.00	0.00	14.76	0.00	0.00	0.00
CTGG	0.00	0.00	0.00	7.09	0.00	0.00	0.00	0.00	0.00	0.00	0.00
CTTG	0.00	0.00	0.00	0.00	0.00	0.00	0.00	0.00	0.00	0.00	6.31
GTAG	0.00	0.00	0.00	0.00	0.00	17.22	0.00	0.00	0.00	0.00	0.00
GTCG	0.00	0.00	0.00	10.70	0.00	0.00	0.00	0.00	0.00	0.00	0.00
GTGG	0.00	0.00	0.00	53.39	0.00	0.00	0.00	0.00	0.00	0.00	0.00
GTTG	0.00	0.00	0.00	7.38	0.00	0.00	0.00	0.00	0.00	0.00	0.00
TTAG	0.00	0.00	0.00	0.00	0.00	0.00	0.00	9.41	0.00	0.00	0.00
TTCG	0.00	0.00	0.00	0.00	14.11	0.00	0.00	0.00	0.00	0.00	0.00
TTGG	0.00	0.00	0.00	0.00	0.00	0.00	0.00	13.24	0.00	0.00	0.00
TTTG	0.00	0.00	0.00	0.00	0.00	0.00	0.00	0.00	0.00	0.00	6.63

**Table A8:** Cross-sectional correlations between 30 COSMICsignatures and cancer types X1–X16 for the exome data summarized in Table 1 aggregated by cancer types. The weights for COSMIC signatures are available from [36]. The values above 80% are given in bold font. The values above 70% are underlined.

Signature	X1	X2	X3	X4	X5	X6	X7	X8	X9	X10	X11	X12	X13	X14	X15	X16
COSMIC1	**89.45**	**94.29**	**80.44**	70.24	67.36	27.61	**90.83**	23.73	27.58	63.3	**90.12**	78.51	**81.18**	**89.33**	**97.03**	49.55
COSMIC2	24.57	9.98	18.61	14.15	6.23	**82.29**	4.8	**81.07**	**81.74**	14.12	19.06	7.69	27.65	2.91	7.96	71.12
COSMIC3	−12.79	−15.52	3.13	−8.38	2.4	17	−12.56	28.33	15.5	−6.34	−2.18	−15.44	−6.58	−22.57	−15.97	15.47
COSMIC4	8.78	−2.48	40.12	−7.33	26.25	−5.15	2.42	−1.54	−5.65	22.44	−1.49	4.36	−0.21	−7.05	−5.54	6.11
COSMIC5	31.29	30.66	27.6	36.36	52.7	7.3	33.06	9.53	3.63	38.61	51.32	34.3	28.43	27.87	27.64	23.26
COSMIC6	75.67	**82.76**	77.34	69.83	72.74	16.2	**92.47**	13.84	16.8	59.95	77.34	78.66	74.2	**92.46**	**84.35**	40.69
COSMIC7	44.18	22.95	22.21	25.98	26.29	51.13	15.5	43.93	49.06	22.89	31.67	18.79	28.88	10.83	21.58	59.96
COSMIC8	12.82	8.08	29.72	1.54	16.56	−4.54	14.91	2.71	−4.65	18.14	18.4	15.45	7.78	3.28	6.33	2.89
COSMIC9	−3.53	−3.98	−10.19	22.79	−2.13	−10.04	1.52	−7.89	−9.83	−3.79	8	15.48	14.23	4.86	−3.85	−12.77
COSMIC10	34.34	17.64	28.23	26.03	14.07	27.2	30.53	20.53	25.27	13.36	19.77	64.86	25.88	23.06	16.43	22.68
COSMIC11	35.2	23.69	19.25	23.9	36.72	27.19	21.53	24.41	24.5	22.07	32.83	15.31	21.76	13.07	26.44	42.54
COSMIC12	−1.61	−2.34	−5.85	4.67	23.61	−5.74	0.37	−5.46	−7.78	9.77	14.57	9.17	−0.39	6.51	−4.08	−1.68
COSMIC13	2.87	−3.44	13.74	1.98	−4.91	71.24	−2.55	76.35	72.85	0.63	3.61	−4.22	18.47	−4.44	0.2	60.08
COSMIC14	52.79	48.45	61.44	45.38	54.47	12.36	74.05	13.53	13.19	35.41	51.75	61.67	47.65	60.64	51.76	31.07
COSMIC15	51.28	50.19	53.9	54.12	57.75	10.52	70.48	9.53	11.22	39.95	49.65	62.24	49.85	71.45	58.85	28.77
COSMIC16	−2.28	−2.35	−0.5	7.28	22.17	8.9	−1.97	11.41	5.71	16.17	18.76	4.3	6.41	−3.4	−6.17	11.89
COSMIC17	−1.47	−0.75	−9.35	44.18	0.18	−0.04	−0.7	−0.65	0.14	3.85	8.05	7.81	39.42	11.2	−0.52	−2.05
COSMIC18	19.89	3.83	43.34	10.48	24.38	6.55	12.58	9.73	6.77	8.44	5.53	25.92	12.85	4.01	1.93	12.79
COSMIC19	42.96	45.14	38.47	37.84	56.04	19.22	41.38	18.9	16.2	36.72	48.65	31.6	34.49	33.11	42.37	39.03
COSMIC20	31.7	35.8	41.02	30.54	48.6	5.94	47.07	5.42	4.3	34.21	40.46	42.75	30.67	41.04	32.11	20.18
COSMIC21	9.79	9.96	4.77	16.56	20.54	−3.68	14.23	−5.29	−4.34	11.95	22.03	19.9	8.42	23.88	11.67	2.69
COSMIC22	−10.52	−9.55	−11.9	−10.11	−11.87	−11.33	−12.09	−12.28	−12.25	52.49	−2.78	−14.12	−14.12	−11.19	−8.46	−11.76
COSMIC23	27.16	25.81	19.44	25.44	42.9	9.57	24.3	7.91	6.39	23.54	27.9	17.59	17.94	17.64	27.65	26.29
COSMIC24	10.03	−0.84	43.35	2.42	27.86	7.29	3.83	9.45	8.17	10.73	−0.15	5.55	6.34	−1.74	−1.97	14.26
COSMIC25	12.26	11.02	11.7	10.08	13.83	6.66	11.16	8.12	4.93	57.53	24.31	13.54	8.95	9.18	9.94	10.21
COSMIC26	13.37	13.38	8.26	17.37	29.37	0.77	20.94	0.15	−0.69	18.19	28.82	25.28	14.23	28.18	14.85	7.4
COSMIC27	−4.19	−4.22	−5.91	1.02	−11.83	−2.66	−4.31	−2.91	−2.63	8.24	−7.21	−4.96	−6.82	−4.21	−3.09	−4.06
COSMIC28	−10.61	−8.93	−19.23	17.31	−11.5	−8.21	−7.57	−8.29	−6.85	−5.6	−7.6	4.11	9.38	0.1	−8.41	−14.27
COSMIC29	26.94	17.44	53.8	14.87	37.87	3.04	23.71	6.25	4.06	19.4	19.72	24.28	21.17	16.51	16.68	14.59
COSMIC30	44.21	32.58	25.34	39.34	37.09	44.06	26.27	41.01	41.95	30.01	41.72	19.63	31.65	19.97	31.06	55.25

**Table A9:** Cross-sectional correlations between 30 COSMIC signatures and cancer types X17–X32 for the exome data summarized in Table 1 aggregated by cancer types. The weights for COSMIC signatures are available from [36]. The values above 80% are given in bold font. The values above 70% are underlined.

Signature	X17	X18	X19	X20	X21	X22	X23	X24	X25	X26	X27	X28	X29	X30	X31	X32
COSMIC1	66.66	22.83	19.86	14.47	48.08	66.58	**80.24**	**83.31**	**94.16**	58.88	78.37	59.4	58.17	**88.27**	76.26	59.17
COSMIC2	13.37	40.04	50.81	10.46	36.67	35.99	8.13	10.26	10.38	9.99	28.05	27.39	2.04	5.91	38.8	10.5
COSMIC3	5.83	31.25	0.86	4.42	9.65	41.57	−16.05	−8.32	−13.9	−12.48	9.73	−2.39	1.36	−14.16	−0.95	−7.39
COSMIC4	1.81	75.05	−2.14	−4.6	49.97	27.1	2.51	−5.36	−1.11	5.96	17.97	21.39	27.4	5.24	−4.28	12.78
COSMIC5	79.6	15.5	26.99	−7.31	10.73	42.45	29.35	49.26	30.41	15.31	53.95	31.44	31.66	34.09	42.47	27.19
COSMIC6	63.89	25.94	8.82	12.01	41.32	62.45	**91.06**	**87.74**	**88.24**	46.22	77.96	50.76	70.16	**88.86**	72.53	58.1
COSMIC7	27.65	31.87	**99.66**	7	36.37	38.52	18.54	25.81	19.5	22.17	38.17	56.58	11.02	16.39	42.31	20.47
COSMIC8	14.72	43.44	−9.3	−5.21	29.34	25.78	10.47	2.31	8.37	18	21.19	14.7	14.47	13.53	0.94	23.61
COSMIC9	15.26	−17.36	−6.58	−14.5	−14.65	−13.15	1.24	−0.21	−1.92	14.47	0.55	−8.02	−8.43	−2.77	−6.73	18.46
COSMIC10	11.48	19.21	18.58	0.86	24.12	19.65	31.75	14.87	20.33	**89.16**	22.14	17.66	10.06	15.29	14.56	**87.49**
COSMIC11	37.02	21.94	77.36	5.72	21.56	36.81	26.72	35.61	20.25	6.45	40.26	48.93	23.07	19.11	41.85	14.48
COSMIC12	50.27	−7.26	1.58	−9.28	−16.64	−2.1	−2.52	16.72	−0.01	−9.43	16.39	−2.97	8.68	8.56	9.4	−2.31
COSMIC13	−2.04	35.65	6.66	−0.38	31.29	38.21	−2.83	1.34	2.46	−0.11	11.23	4.34	−2.61	−3.21	22.91	−0.89
COSMIC14	38.55	36.5	14.94	5.86	43.08	49.41	76.65	56.58	54.4	38.22	57.5	54.34	59.64	59.99	46.35	58.74
COSMIC15	42.05	14.95	7.14	9.89	27.61	41.95	**82.98**	76	63.73	32.73	56.75	40.14	62.71	68.42	53.87	46.38
COSMIC16	56.26	10.38	9.23	−13.64	−4.46	13.69	−4.71	15.75	−1.59	−3.7	20.56	2.25	4.06	3.41	15.09	1.88
COSMIC17	1.26	−11.27	4.05	−2.77	−8.56	−3.3	0.91	−0.68	0.94	3.56	3.22	−0.83	4.77	3.02	3.12	3.93
COSMIC18	1.08	55.29	−1.04	6.43	53.97	23.12	15.21	0.28	8.82	32.61	18.54	33.36	24.02	10.97	1.16	38.89
COSMIC19	55.06	26.57	48.76	5.01	23.54	50.79	44.61	54.38	39.57	14.42	57.89	47.64	45.4	40.3	52.16	25.02
COSMIC20	55.9	31.1	9.54	2.85	19.92	37.82	40.79	43.95	34.15	17.31	52.43	24.8	42.43	40.32	39.66	35.22
COSMIC21	33.38	−12.86	0.05	−6.89	−9.66	0.23	12.35	21.42	15.34	2.9	15.33	2.96	6.55	18.55	14.91	8.78
COSMIC22	−7.62	−1.77	−7.78	−12.32	−18.87	−15.29	−12.86	−12.47	−11.18	−8.83	−2.13	−13.97	−20.14	−11.76	−10.2	−12.77
COSMIC23	37.74	14.67	47.26	2.88	12.21	33.83	30.51	39.51	22.27	4.53	40.81	36.52	33.83	21.75	38.16	14.34
COSMIC24	−3.48	64.69	−1.2	1.23	67.23	26.96	9.63	−0.1	5.17	6.47	15.79	36.14	40.4	8.82	0.64	11.09
COSMIC25	26.48	14.87	−0.68	−6.78	−0.38	12.12	6.88	11.3	9.67	13.23	23.94	4.69	−3.35	9.51	18.18	14.51
COSMIC26	54.08	−8.65	0.08	−6.4	−9.68	6.33	17.87	33.03	18.87	3.97	25.39	4	12.13	24.86	22.75	11.68
COSMIC27	−5.21	−5.53	−0.83	−1.13	−5.61	−10.71	−3.71	−4.84	−4.08	−3.2	15.48	−4.27	−10.11	−6.96	−6.57	−4.13
COSMIC28	−5	−21.81	−5.79	−8.13	−18.66	−17.36	−5.6	−10.39	−8.43	3.09	−5.96	−13.41	−15.94	−10.54	−11.47	3.99
COSMIC29	14.75	60.17	−4.79	5.76	71.38	34.28	23.68	12.79	22.55	21.94	27.84	45.59	43.15	26.32	10.28	27.23
COSMIC30	41.46	26.37	76.08	5.97	30.05	45.82	27.99	38.08	27.75	8.55	48.65	51.88	23.83	23.84	51.58	15.89

**Table A10:** Cross-sectional correlations between 30 COSMIC signatures and cancer types G.X1–G.X14 for the genome data summarized in Table 1 of [16] aggregated by cancer types. G.X1 = B-cell lymphoma, G.X2 = bone cancer, G.X3 = brain lower grade glioma, G.X4 = breast cancer, G.X5 = chronic lymphocytic leukemia, G.X6 = esophageal cancer, G.X7 = gastric cancer, G.X8 = liver cancer, G.X9 = lung cancer, G.X10 = medulloblastoma, G.X11 = ovarian cancer, G.X12 = pancreatic cancer, G.X13 = prostate cancer, G.X14 = renal cell carcinoma. The weights for COSMIC signatures are available from [36]. The values above 80% are given in bold font. The values above 70% are underlined.

Signature	G.X1	G.X2	G.X3	G.X4	G.X5	G.X6	G.X7	G.X8	G.X9	G.X10	G.X11	G.X12	G.X13	G.X14
COSMIC1	39.38	**86.27**	**91.05**	15.44	69.73	74.43	48.71	−2.93	6.59	**94.86**	46.94	**95.31**	**83.27**	19.48
COSMIC2	47.64	22	18.39	79.91	11.54	54.23	7.52	−10.44	32.48	14.39	32.28	12.87	27.25	22.72
COSMIC3	11.51	10.77	1.15	36	10.73	6.03	4.29	1.37	49.07	1.04	60.73	−16.4	13.8	36.19
COSMIC4	−10.31	16.21	0.21	1.07	1.06	1.39	−2.62	8.44	**82.53**	7.79	26.95	−3.57	17.3	28.1
COSMIC5	55.4	57.76	55.72	13.06	75.02	22.07	46.35	1.32	29.05	48.62	63.51	24.5	51.15	54.23
COSMIC6	33.24	66.83	71.52	6.37	58.41	59.46	50.19	−4.93	8.23	76.42	37.65	**86.72**	66.87	11.58
COSMIC7	42.62	26.5	30.74	39.25	22.51	36.55	7.21	0.22	18.84	23.95	22.82	19.38	25.4	21.84
COSMIC8	15.64	46.16	22.68	7.68	37.63	9.85	20.6	−4.54	68.63	31.67	57.31	7.37	44.86	54.24
COSMIC9	62.32	14.33	12.05	−3.79	51.08	−6.83	60.78	−13.48	−0.57	9.82	16.12	−1.57	18.48	31.51
COSMIC10	27.68	28.48	22.43	11.9	23.97	30.68	25.05	1.74	20.12	30.22	18.54	21.41	35.38	13.93
COSMIC11	36.78	28.03	31.95	22.49	29.37	22.68	8.2	6.52	13.79	25.95	22.77	17.48	21.58	20.6
COSMIC12	23.88	14.26	16.17	−1.85	34.02	−6.63	23.78	−3.79	3.57	7.7	23.78	−4.48	9.78	18.13
COSMIC13	13.66	4.28	2.33	**80.9**	−4.65	47.36	−1.17	−14.68	28.78	0.59	43.56	3.99	16.71	10.38
COSMIC14	30.05	52.91	44.83	9.22	44.04	39.25	39.49	−2.03	30.51	53.35	36.4	53.72	52.42	16.22
COSMIC15	25.67	42.18	41.37	4.53	39.78	35.89	34.97	−0.46	2.31	48.67	24.49	58.99	46.22	5.51
COSMIC16	45.02	27.55	25.39	18.48	50.43	4.96	32.15	−1.05	27.99	14.62	49.19	−6.52	24.2	44.42
COSMIC17	54.33	−2.69	2.27	−0.59	19	0.43	73.87	−3.68	−11.64	−2.52	−3	2.2	−2.89	−2.4
COSMIC18	8.56	27.17	8.01	9.55	14.24	15.81	11.6	4.2	66.55	18.73	24.89	6.48	31.09	18.59
COSMIC19	37.46	48.08	56.92	17.83	49.29	30.71	20.98	3.49	20.9	45.85	40.02	34.23	39.33	28.58
COSMIC20	22.09	35.41	36.98	2.45	36.13	23.82	32.67	−7.1	25.78	34.99	28.96	32.35	28.42	12.46
COSMIC21	12.23	15.22	18.41	−6.01	23.79	3.96	16.11	1.44	−12.08	14.86	8.33	11.16	12.87	−1.38
COSMIC22	−15.62	−7.33	−10.46	−10.43	−7	−8.75	−13.39	−16.17	2.97	−11.57	−5.9	−9.23	−8.08	47.87
COSMIC23	23.12	24.08	34.44	7.06	29.31	12.74	8.41	5.5	7.78	24.45	17.62	16.66	16.08	15.21
COSMIC24	−6.8	12.77	−1.61	10.16	−3.47	10.01	−3.98	10.27	62.16	6.07	16.1	1.86	14.9	6.58
COSMIC25	15.8	28.11	20.81	10.07	29.92	16.63	16.12	−21.2	25.42	19.51	33.86	10.23	27.09	61.26
COSMIC26	25	24.23	25.82	0.87	37.31	7.71	27.11	0.51	−4.25	21.13	24.3	14.95	20.76	8.6
COSMIC27	−4.17	−0.03	−4.34	−2.16	−2.63	−4.3	−7.7	−8.59	2.35	−3.32	−4.12	−3.36	0.47	35.18
COSMIC28	42.33	−9.94	−3.87	−6.62	16.24	−11.59	52.44	3.29	−18.66	−8.75	−7.25	−5.64	−5.71	12.33
COSMIC29	7.41	38.85	21.13	5.93	23.52	20.41	14.98	4.24	68.09	31.18	33.47	19.68	38.55	16.98
COSMIC30	49.59	37.46	41.49	38.84	35.91	39.02	16.17	0.96	15.15	34.52	27.39	26.86	32.02	24.89
